# A midbrain-thalamus-cortex circuit reorganizes cortical dynamics to initiate movement

**DOI:** 10.1016/j.cell.2022.02.006

**Published:** 2022-03-03

**Authors:** Hidehiko K. Inagaki, Susu Chen, Margreet C. Ridder, Pankaj Sah, Nuo Li, Zidan Yang, Hana Hasanbegovic, Zhenyu Gao, Charles R. Gerfen, Karel Svoboda

**Affiliations:** 1 Janelia Research Campus, HHMI, Ashburn, VA, 20147, USA.; 2 Max Planck Florida Institute for Neuroscience, Jupiter, FL, 33458, USA.; 3 Department of Neuroscience, Physiology, and Pharmacology, University College London, London WC1E 6BT, UK.; 4 Queensland Brain Institute, The University of Queensland, Brisbane, QLD, 4072, Australia.; 5 Joint Center for Neuroscience and Neural Engineering, and Department of Biology, Southern University of Science and Technology, Shenzhen, Guangdong Province, 518055, P. R. China.; 6 Department of Neuroscience, Baylor College of Medicine, Houston, TX, 77030, USA.; 7 Department of Neuroscience, Erasmus MC, Rotterdam, 3015GE, The Netherlands.; 8 National Institute of Mental Health, Bethesda, MD, 20892, USA.; 9 Allen Institute for Neural Dynamics, Seattle, WA, 98109, USA.; 10 These authors contributed equally.; 11 Lead contact.

**Keywords:** State-space, dimensionality reduction, midbrain locomotor region, silicon probe, Neuropixels, spikes, optogenetics, short-term memory, licking, motor control

## Abstract

Motor behaviors are often planned long before execution, but only released after specific sensory events. Planning and execution are each associated with distinct patterns of motor cortex activity. Key questions are how these dynamic activity patterns are generated and how they relate to behavior. Here we investigate the multi-regional neural circuits that link an auditory ‘go cue’ and the transition from planning to execution of directional licking. Ascending glutamatergic neurons in the midbrain reticular and pedunculopontine nuclei show short-latency and phasic changes in spike rate that are selective for the go cue. This signal is transmitted via the thalamus to the motor cortex, where it triggers a rapid reorganization of motor cortex state from planning-related activity to a motor command, which in turn drives appropriate movement. Our studies show how midbrain can control cortical dynamics via the thalamus for rapid and precise motor behavior.

## ETOC

Ascending midbrain neurons trigger rapid reorganization of motor cortex activity via thalamus to switch from planning-related activity to a motor command that initiates movement in mice

## Graphical Abstract

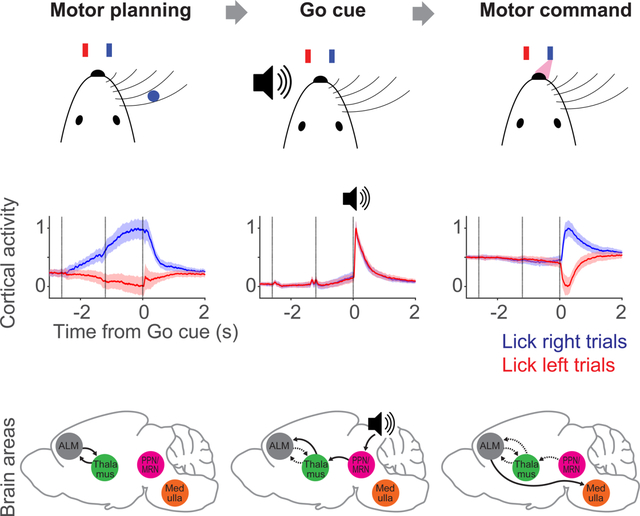

## Introduction

Many behaviors, including purposeful movements, are composed of sequential phases that require different computations. For example, while waiting at a red light to make a turn, we plan to rotate the steering wheel while pressing the gas pedal. After the signal turns green we achieve our goal by executing a program of skilled movements effortlessly. The planning and execution phases are produced by distinct patterns of neuronal activity (Svoboda and Li, 2018; Vyas et al., 2020). In laboratory decision-making tasks, behavior-related neural activity rapidly switches from one pattern to another at the appropriate time, often guided by contextual cues (Funahashi et al., 1989; Sommer and Wurtz, 2002; Kaufman et al., 2016, 2014; Vyas et al., 2020).

Planned movements that are released by a contextual “Go cue” are faster and more precise than unplanned movements (Hanes and Schall, 1996; Rosenbaum, 1980; Shenoy et al., 2013; Duan et al., 2021). Planned movements are anticipated by slowly varying neuronal activity in multiple connected brain areas, including the motor cortex (MCx), non-sensory thalamus, and others (Tanji and Evarts, 1976; Tanaka, 2007; Shenoy et al., 2013; Guo et al., 2017; Gao et al., 2018; Svoboda and Li, 2018). This ‘preparatory activity’ encodes specific upcoming movements, often seconds before movement onset (Tanji and Evarts, 1976). Cortical activity then changes rapidly and profoundly just before movement onset (Guo et al., 2014b; Kaufman et al., 2014, 2016).

Recordings from large populations of neurons have enabled state-space analysis of neural activity (Laurent, 2002; Stopfer et al., 2003). With *n* recorded neurons, population activity can be represented as a trajectory in *n*-dimensional activity space. These trajectories are typically confined to a low-dimensional manifold, defined by several ‘activity modes’ that explain a significant proportion of the population activity. Activity modes can be obtained by projecting neural activity along specific directions in neural state space, or similar dimensionality-reduction methods (Cunningham and Yu, 2014; Kaufman et al., 2014; Kobak et al., 2016; Li et al., 2016). A successful decomposition of neural activity provides activity modes that are interpretable, by predicting specific aspects of behavior and revealing related neural computations (Mante et al., 2013; Kobak et al., 2016; Li et al., 2016; Inagaki et al., 2019; Finkelstein et al., 2019; Vyas et al., 2020; Lee and Sabatini, 2021).

For example, during motor planning, preparatory activity in MCx occupies an activity mode that discriminates future movement types. This activity mode follows attractor dynamics and funnels preparatory activity to an initial condition (a fixed point) appropriate to trigger accurate and rapid movements (Churchland et al., 2010; Shenoy et al., 2013; Inagaki et al., 2019). After the Go cue, the dynamics in MCx shows large changes. The motor planning mode collapses (Funahashi et al., 1989; Shadlen and Newsome, 2001; Kaufman et al., 2016, 2014) and a new activity mode with multi-phasic dynamics emerges (Churchland et al., 2012). This movement-type specific mode is preferentially represented in the descending MCx neurons that project to premotor neurons in the brainstem and spinal cord (Li et al., 2015; Economo et al., 2018; Duan et al., 2021) and presumably serves as part of a motor command to initiate a specific movement. Another activity mode after the Go cue consists of changes that are invariant to the movement type (condition-invariant signal (Kaufman et al., 2016)), referred to here as ‘Go cue direction’ (**D**_**go**_) mode. Altogether, when an animal releases a planned action following a Go cue, neuronal activity in MCx transforms from a motor planning mode (i.e. preparatory activity) to a motor-command mode and a **D**_**go**_ mode. These modes occupy near-orthogonal subspaces, which may explain in part why movements are not triggered during planning (Kaufman et al., 2014; Elsayed et al., 2016).

Neuronal dynamics underlying motor planning and execution have been studied in non-human primates and rodents trained in delayed-response tasks (Funahashi et al., 1989; Riehle and Requin, 1989; Erlich et al., 2011; Shenoy et al., 2013; Guo et al., 2014b). An instruction informs movement type (e.g. movement direction or target; eye, tongue, arm or orienting movements) and a Go cue after a delay releases planned actions and thereby movement onset. The anterior lateral motor cortex (ALM), a part of MCx, is necessary for motor planning and execution of directional licking in mice (Komiyama et al., 2010; Guo et al., 2014b; Gao et al., 2018; Xu et al., 2019; Esmaeili et al., 2021; Bollu et al., 2021). Stimulation of ALM triggers rhythmic licking (Komiyama et al., 2010; Li et al., 2015). ALM forms reciprocal connections with parts of the thalamus (referred to as ALM-projecting thalamus, or thal_ALM_) to maintain the motor plan (Guo et al., 2017). Thal_ALM_ receives input from the basal ganglia, cerebellum, and the midbrain, which directly or indirectly receive input from ALM (Guo et al., 2017; Gao et al., 2018). Thus, thal_ALM_ is a hub linking subcortical structures and ALM, forming multi-regional loops essential for orofacial movements.

Here we mapped the mechanisms underlying cue-triggered switching of activity modes and the resulting movement initiation, in the context of a delayed directional licking task (Guo et al., 2014b). By combining anatomy and large-scale electrophysiological recordings we established that Go cue-related information flows from the midbrain to ALM via thal_ALM_, where it initiates **D**_**go**_ signals and motor command-like dynamics in ALM, followed by appropriate movements. Altogether, we have identified a multi-regional pathway mediating cue-triggered mode switching for the release of planned movements.

## Results

### A mode switch before movement initiation

**Figure 1.**
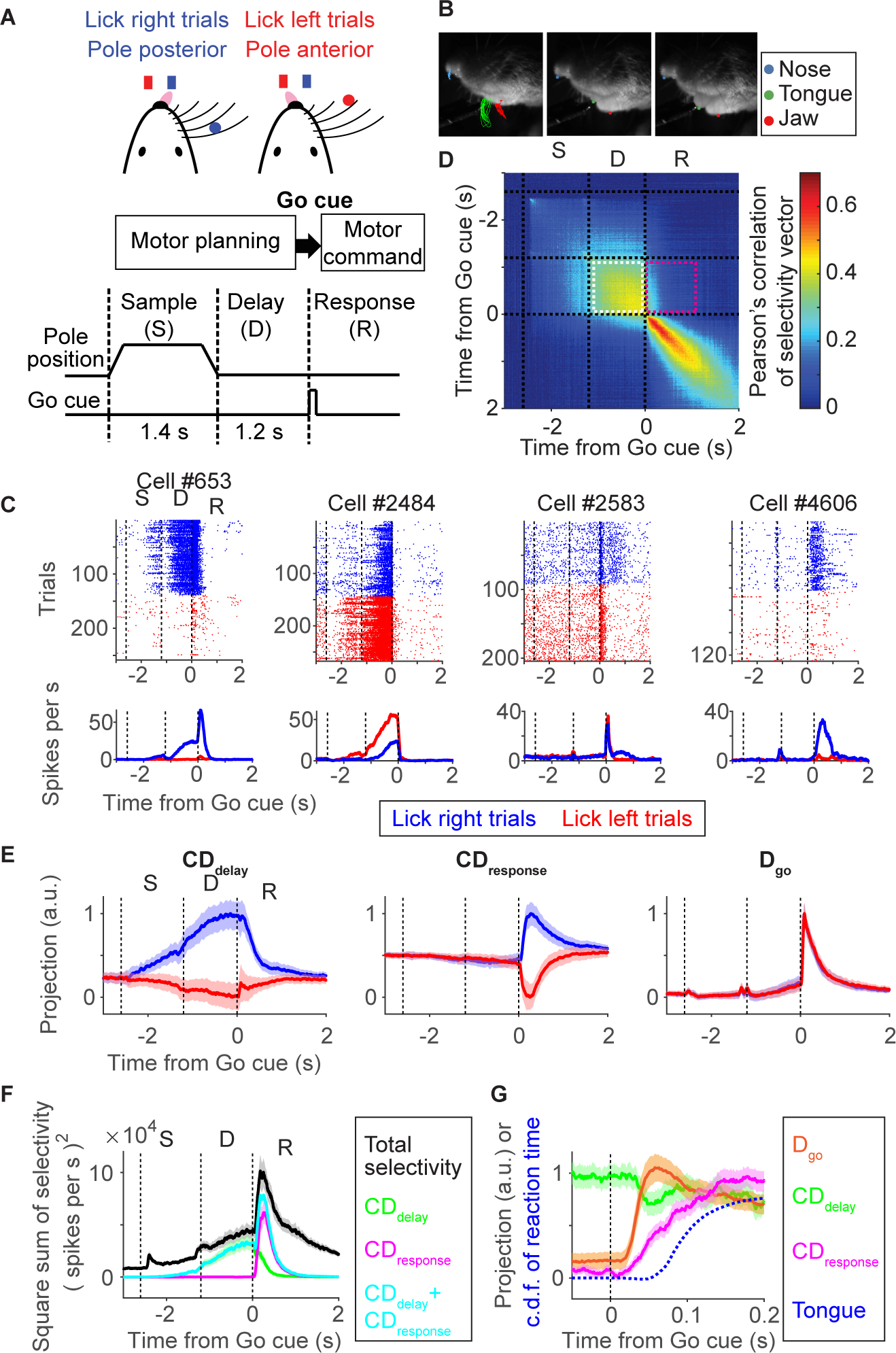
Activity modes for motor planning and movement initiation in anterior lateral motor cortex A. Tactile delayed-response task. Red / blue circles, tactile stimulus. B. Side view of a behaving mouse recorded with high-speed videography. Left, trajectories of nose (blue), tongue (green) and jaw (red) movement overlaid on an image at onset of the Go cue (time, 0). Middle, the first frame at which the tongue appears. C. Example neurons in ALM. Top, spike raster. Bottom, mean spike rate. Blue, correct lick right trials; red, correct lick left trials. Dashed lines separate behavioral epoch. D. Pearson’s Correlation of the population activity vector in ALM (bin: 10 ms; *n* = 5136 neurons). E. Projections of activity along **CD**_**delay**_, **CD**_**response,**_ and **D**_**go**_. Line, median. Shading, S.E.M. F. Selectivity explained by each direction in activity space. Square sum of selectivity of all recorded ALM neurons (black) or along each mode (colors). G. Onset of each mode. Orange, activity along **D**_**go**_ (mean of activity in lick right and left trials). Green and magenta, activity along **CD**_**delay**_ and **CD**_**response**_ (difference in activity between lick right and left trials). Dashed blue line, cumulative distribution of the first tongue detection (after the timing of the Go cue) by the videography. See also Figure S1.

We studied head-restrained mice performing a delayed-response task (Guo et al., 2014b) (Figures 1A and 1B, Movie S1). A tactile stimulus, an object presented to the right whiskers at one of two locations during the sample epoch, instructed lick direction (left or right). Mice were trained to withhold licking during the following delay epoch (1.2 s). After an auditory Go cue (3 or 3.4 kHz, 0.1s), licking in the correct direction was rewarded. In this task mice plan upcoming movements during the delay epoch and release planned movements following the Go cue.

We performed extracellular recordings in left ALM (5136 putative pyramidal neurons). Consistent with previous reports (Guo et al., 2014b; Inagaki et al., 2018), ALM neurons showed spike rates selective for lick direction (selectivity; *p* < 0.05, ranksum test) during the delay (1926/5136 neurons) and the response epochs (2641/5136 neurons). An early hypothesis suggested that preparatory activity is a subthreshold version of the activity that later causes the movement (Tanji and Evarts, 1976). This would imply that the Go cue enhances each neuron’s preparatory activity to trigger movement. Some neurons have activity consistent with this view. For example, cell #653 shows delay selectivity, and consistent selectivity peaks after the Go cue (Figure 1C). More generally, activity patterns changed qualitatively after the Go cue (Figures 1C, S1A, and S1D) (Kaufman et al., 2014). For example, cell #2484 shows lick-left selectivity during the delay epoch, but the selectivity collapses during the response epoch. In addition, a subset of cells (177/5136 cells) switched selectivity. The simple notion that preparatory activity is a subthreshold motor command therefore does not explain ALM activity around the time of movement initiation.

To quantify how movement-related selectivity in ALM evolves at a population level we analyzed neural dynamics in activity space (Cunningham and Yu, 2014). We defined a population selectivity vector: ***w***_*t*_ = ***r***_lick-right, *t*_ – ***r***_lick-left, *t*_, where ***r***_lick-right, *t*_ and ***r***_lick-left, *t*_ are vectors of spike rate of individual neurons for each time *t*, averaged over lick right and left trials, respectively (the number of elements in the vector equals the number of recorded neurons). Pearson’s correlation of this population selectivity vector is high across time within the delay epoch (Figure 1D, a box with white dotted outline), implying that a similar combination of ALM neurons maintains selectivity during motor planning (Li et al., 2016; Economo et al., 2018; Inagaki et al., 2018). In contrast, population selectivity has a low correlation between time points before and after the Go cue (Figure 1D, magenta box), implying that different combinations of neurons show selectivity. Similarly, the population activity of ALM neurons within each trial type (***r***_lick-right, *t,*_ or ***r***_lick-left, *t*_) shows low correlation before versus after the Go cue (Figure S1B). Moreover, intracellular recordings of ALM neurons show that membrane conductances increase rapidly after the Go cue, driven by reorganization of synaptic input after the Go cue, which presumably supports the switch in population activity patterns (Figures S1E and S1F). Altogether, these results imply that the population activity patterns in ALM change rapidly before and during movement initiation. Similar changes in activity have been observed across species and behavioral tasks (Funahashi et al., 1989; Shadlen and Newsome, 2001; Maimon and Assad, 2006; Vyas et al., 2020).

The stable preparatory activity during the delay epoch (Figures 1C, 1D, S1A and S1B) suggests a low-dimensional representation of ALM population activity. We defined a delay coding direction $CD_{de\mathrm{lay}}=\bar{w_{t}}$ (−0.6 s < t < 0 s; time to Go cue) as the direction in activity space that discriminates future lick directions (lick left or right) during the delay epoch. Consistent with previous studies, this direction contains almost all movement-direction selective activity before the Go cue and allows decoding of lick direction one second before movement (Figures 1E and S1I) (Li et al., 2016; Inagaki et al., 2019). Similarly, we defined $CD_{\mathrm{response}}=\bar{w_{t}}$ (0 s < t < 0.4 s; time to Go cue) as a direction that discriminates lick directions after the onset of the Go cue. We orthogonalized **CD**_**response**_ to **CD**_**delay**_ to isolate activity patterns that emerge after the Go cue (Economo et al., 2018) (Figure S1G and S1H). Activity along **CD**_**response**_ contains a large proportion of direction-selective activity and allows decoding of movement (Figures 1E and S1I–K). These two modes together explain 71.2 (65.3–76.0) % (mean, 2.5 – 97.5 % confidence interval) of selectivity in ALM around the movement initiation (±200 ms from the Go cue; Figure 1F, cyan line).

Activity projected onto **CD**_**delay**_ and **CD**_**response**_ is correlated at the level of single trials (Figure S1L). This implies that information carried along **CD**_**delay**_ is transferred to **CD**_**response**_ following the Go cue (i.e., trials with strong activity along **CD**_**delay**_ have strong activity along **CD**_**response**_, and vice versa). This finding is consistent with the observation that fine scale movement parameters and reaction times are coded in preparatory activity (unpublished observations) (Li et al., 2016; Even-Chen et al., 2019) and implies that ALM preparatory activity (activity along **CD**_**delay**_) contributes to control of future movements (activity along **CD**_**response**_).

We also find phasic non-selective activity after the Go cue in ALM (e.g., cell #2583 in Figure 1C). At the population level we defined a direction that discriminates activity before and after the Go cue, **D**_**go**_ ***= r***_*after Go cue*_ – ***r***_*before Go cue*_, averaged over all correct trials (100 ms time window). Activity along **D**_**go**_ explains a large proportion of ALM activity after the Go cue (Figure S1I), similar to the ‘condition-invariant signal’ described in a primate reaching task (Kaufman et al., 2016). Activity along **D**_**go**_ is non-selective (Figure 1E) and cannot decode lick direction (Figure S1J), because activity changes around the Go cue are largely similar across trial types (Figure S1C). The trial-type differences that do exist contribute to **CD**_**response**_. These three directions in activity space, together with a fourth direction that captures non-selective ramping activity during the delay epoch (Li et al., 2016; Inagaki et al., 2018), account for nearly all (87.4, 84.8–89.6 %; mean, 2.5–97.5% confidence interval) of population activity around the Go cue (±200 ms from the Go cue; Figure S1I, cyan line).

Consistent with single neuron dynamics, activity along **D**_**go**_ and **CD**_**response**_ changed rapidly after the Go cue (latencies, 20.0 (16–24) ms, 30.4 (18–44) ms, respectively; mean (2.5–97.5% confidence interval); Methods; Figure 1G). These changes precede movement onset (64.3 (56–75) ms; mean (2.5–97.5% confidence interval); blue dashed line in Figure 1G). Because activity along **D**_**go**_ and **CD**_**response**_ precede movement (Figure S1K), and because silencing of ALM results in loss of cued licking (Komiyama et al., 2010; Gao et al., 2018; Xu et al., 2019; Esmaeili et al., 2021; Bollu et al., 2021), we hypothesized that the mode switch is essential to initiate planned movement. We tested the hypothesis that the Go cue triggers non-selective **D**_**go**_ signals in ALM, which then reorganizes movement-type selective activity from **CD**_**delay**_ to **CD**_**response**_ to initiate movement.

### Mode switches without licking

**Figure 2.**
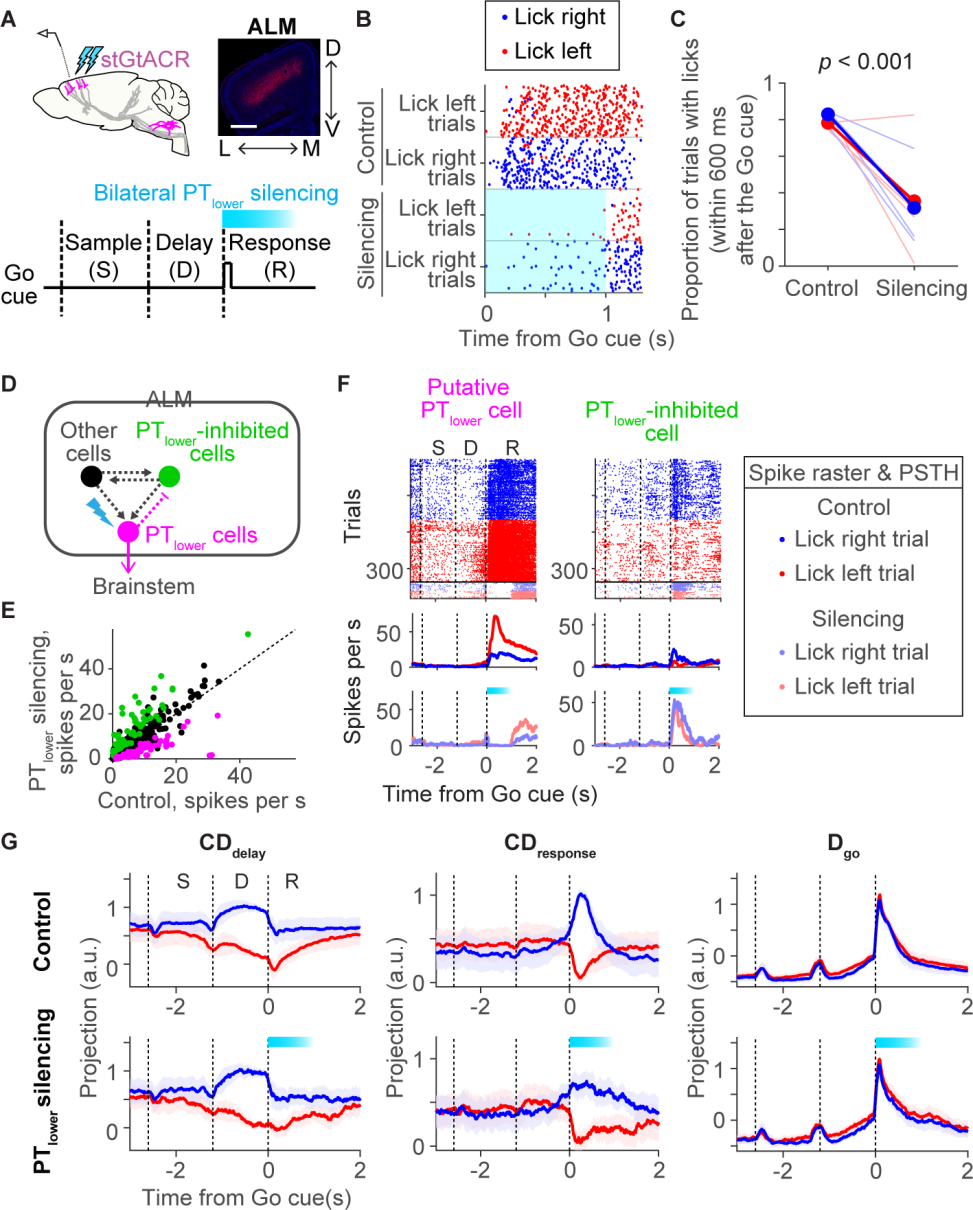
Attenuated CD_response_ but intact D_go_ with silencing of ALM output A. Silencing PT_lower_ cells in ALM. Inset, coronal section showing PT_lower_ cells in ALM expressing GtACR1 fused with FusionRed; Blue, DAPI. D, dorsal; V, ventral; M, medial; L, lateral. B. Lick timing in an example animal (75 trials per trial type). Cyan, laser on. C. Proportion of licks within 600 ms after the Go cue. Blue, lick right trials; red, lick left trials. Circle, mean; lines, each animal (n = 4). *P* < 0.001 in both lick right and left trials (hierarchical bootstrap with a null hypothesis that proportion of trials with licks in silencing trials are the same or higher than that in control). D. Schema showing cell types analyzed in Figure 2 and S2. PT_lower_ cells (magenta) indirectly inhibit PT_lower_-inhibited cells (green). E. Spike rate of individual neurons with or without PT_lower_ silencing. Circles, individual neurons; magenta; significantly decreased neurons (putative PT_lower_ cells); green, significantly increased neurons (PT_lower_ inhibited cells). F. Example putative PT_lower_ and PT_lower_-inhibited cells. Top, spike raster. Bottom, PSTH in control and PT_lower_ silencing trials. Blue, all lick right trials; red, all lick left trials: cyan bar, laser on. G. Projection of activity along **CD**_**delay**_, **CD**_**response,**_ and **D**_**go**_ with and without PT_lower_ silencing. Line, grand median across sessions (n = 24 sessions; 4 mice); shading, S.E.M. (*hierarchical bootstrap*); cyan bar, laser on. See also Figure S2.

Our hypothesis predicts that although both **D**_**go**_ and **CD**_**response**_ appear after the Go cue, they may be dissociable with manipulation of the **CD**_**response**_. We silenced ALM neurons projecting to the medulla (pyramidal tract neurons in lower Layer 5b, ‘PT_lower_’) which contribute disproportionately to the **CD**_**response**_ (Economo et al., 2018). Because the medulla contains the motor centers for orofacial movement (Travers et al., 1997; Stanek et al., 2014), descending signals from ALM to the medulla may be necessary for movement initiation. We injected AAV_retro_ (Tervo et al., 2016) encoding soma-targeted (st) GtACR1 (Govorunova et al., 2015; Mahn et al., 2018) in the medulla (Figures 2A and S2A; Table S1 and S3). Bilateral optogenetic silencing of PT_lower_ cells in ALM (centered at AP 2.5 mm L 1.5 mm from Bregma; 1 second of photostimulation starting at the onset of Go cue) resulted in a loss of cue-triggered licking (Figures 2B, 2C, S2B and S2C). Similar bilateral silencing in posterior cortical regions (centered around AP 0 mm L 1.5 mm from Bregma) had a weaker behavioral effect (Figure S2C). Following the end of the photostimulus, mice licked in the correct direction (Figures 2B and S2D), implying that activity of ALM PT_lower_ cells is required to initiate movements, but not to maintain motor plans and not for a memory of the Go cue.

We next performed extracellular recordings in ALM during this manipulation, using photostimulation powers that produced significant behavioral effects (0.5 mW; Figure S2C). Spike rates were altered in 248/899 cells (*p* < 0.05, ranksum test; Figures 2D–2F, S2E and S2F). Cells silenced by the photostimulus (150/899) could include PT_lower_ cells and neurons excited by them directly or indirectly (Figures 2D–F; putative PT_lower_ cells), whereas excited cells (129/899) are neurons indirectly inhibited by PT_lower_ cells (Figures 2D–2F; PT_lower_-inhibited cells). Strongly silenced cells (*p* < 0.001, ranksum test) were in deep cortical layers (821 ± 185μm; mean ± std.; 56 cells), consistent with the depths of PT_lower_ cells (Economo et al., 2018).

The PT_lower_ silencing attenuated activity along **CD**_**response**_ in lick-right trials (when recording in the left hemisphere), without affecting activity along **D**_**go**_ (Figures 2G and S2J). The contralateral reduction in **CD**_**response**_ does not simply reflect silencing of PT_lower_ cells, but is a network effect. First, PT_lower_ cells are a small proportion of ALM neurons and thus make a correspondingly small contribution to **CD**_**response**_ (Figure S2A) (Economo et al., 2018). Second, putative PT_lower_ cells do not have strong contralateral selectivity on average, and the extent of silencing is equal between trial types (i.e. no contralateral bias in putative PT_lower_ cells; Figures S2F and S2G). Third, **CD**_**response**_ based on non-PT_lower_ cells alone shows a contralateral reduction in **CD**_**response**_ activity during PT_lower_ silencing (Figure S2G). Although PT_lower_ cells have only weak connections with other pyramidal cells (Brown and Hestrin, 2009; Kiritani et al., 2012), they may influence the network via their connections to local GABAergic interneurons or through multi-regional loops (Svoboda and Li, 2018). These experiments imply that **D**_**go**_ develops independent of **CD**_**response**_.

Additional support for distinct roles of **D**_**go**_ and **CD**_**response**_ comes from analysis of trials in which mice failed to lick after the Go cue (no response trials; mostly near the end of a session when they are satiated). Activity along **CD**_**delay**_ is attenuated during the delay epoch in these trials likely modulated by the motivational state of the animal (Allen et al., 2019) (Figures S2I and S2J). Although activity along **D**_**go**_ increases, activity along **CD**_**response**_ does not develop after the Go cue (Figures S2I and S2J). Thus, even when activity along **D**_**go**_ appears after the Go cue, without properly developed motor planning (**CD**_**delay**_), activity along **CD**_**response**_ does not emerge.

These experiments together show that descending output from PT_lower_ cells is required for movement initiation. Furthermore, activity along **CD**_**response**_, which is encoded by PT_lower_ and other cells (Economo et al., 2018), instructs lick direction and develops before movement execution. These results are consistent with a view that activity along **CD**_**response**_ is part of the motor command for directional licking.

In contrast, activity along **D**_**go**_ precedes movement but it is not instructive on movement type or sufficient to trigger movement by itself (without a change in activity along **CD**_**response**_). Thus, activity along **D**_**go**_ is not a motor command. Instead, **D**_**go**_ may trigger the activity along **CD**_**response**_ following the Go cue. Testing this hypothesis requires manipulations of activity along **D**_**go**_ by activating or inhibiting neurons that carry this signal. This requires mapping the pathways that transmit Go cue related signals to ALM.

### Thalamus conveys the Go cue signal to ALM

To explore the causal chain of events leading from an auditory Go cue to movement initiation, we analyzed rapid changes in activity after the Go cue and compared latencies across brain areas. ALM forms strong reciprocal connections with thal_ALM_, including parts of the ventral medial (VM), ventral anterolateral (VAL), mediodorsal (MD), paracentral (PCN), central lateral (CL), central medial (CM), and parafascicular (PF) nuclei of the thalamus (Guo et al., 2017). The PCN, CL, CM, and PF comprise the so-called intralaminar (IL) nuclei of the thalamus.

**Figure 3.**
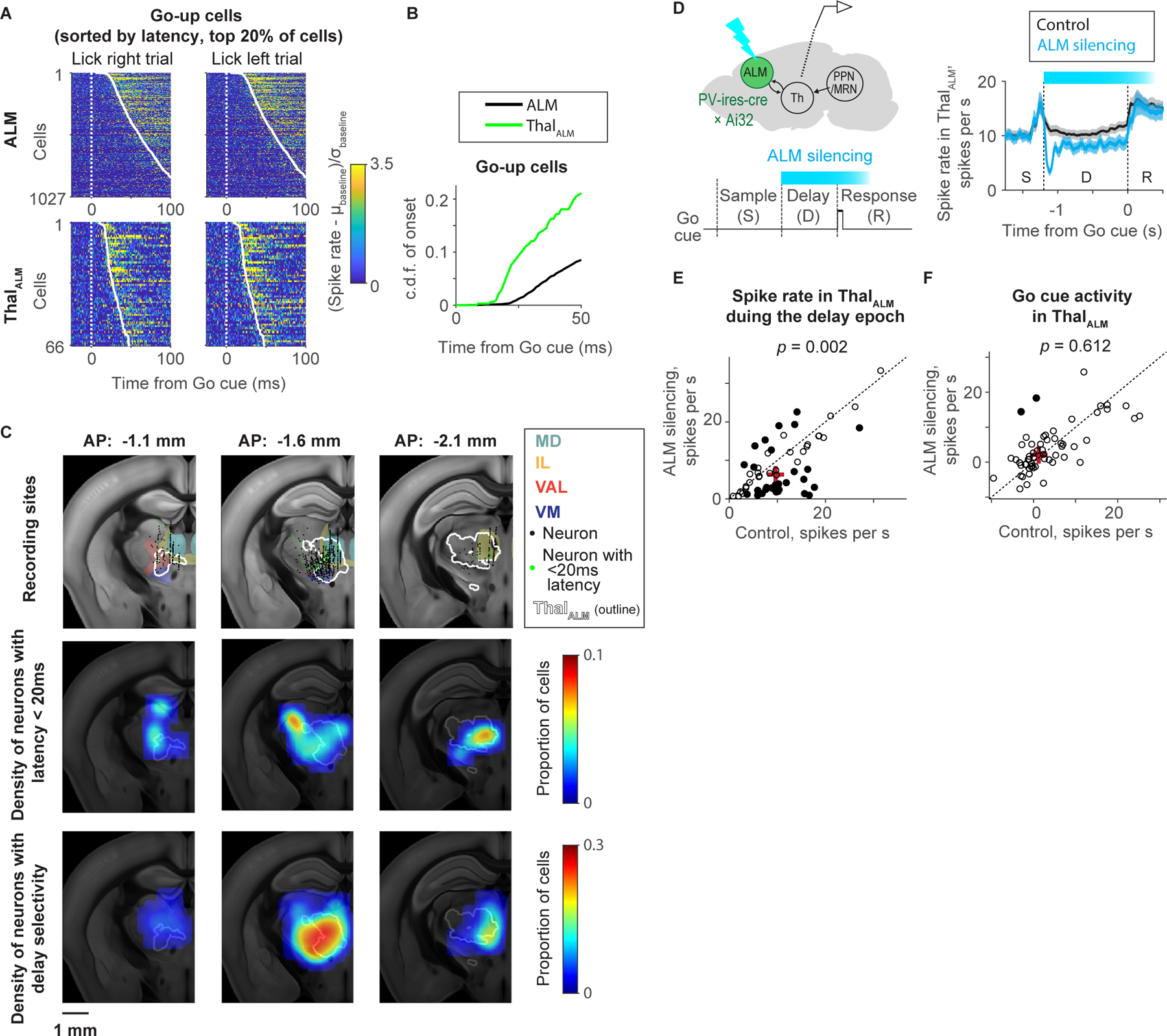
Short latency Go cue signals in ALM-projecting thalamus A. Go cue responses of ALM (top) and thal_ALM_ (bottom) neurons sorted by latency. Cells with increases in spike rate (go-up cells) are shown. Spike rates are z-scored (by the baseline before the Go cue, 100 ms window) for each neuron. μ, mean; σ, standard deviation. B. Cumulative distribution function (c.d.f.) of latency to the Go cue across neurons in ALM and thal_ALM_. C. Top, recording sites in Allen Common Coordinate Framework (CCF). Colored regions, thalamic nuclei. White contour, thal_ALM_. Black, individual neurons. Green, neurons with < 20 ms latency. Middle, the density of neurons with latencies < 20 ms. Bottom, the density of neurons with delay selectivity. AP, posterior to Bregma. D. Recording in thal_ALM_ during ALM silencing. Left, schema. Right, mean activity of thal_ALM_ with or without ALM silencing. Cyan bar, photoinhibition of ALM. E. Spike rate during the delay epoch in thal_ALM_ with or without ALM silencing. Circle, individual neuron in thal_ALM_ (n = 58 cells). Filled circle, significantly modulated cells (*p* < 0.01, ranksum test). Cross, median activity. *P-value*, signed rank test comparing spike rate across neurons with or without silencing. F. The amplitude of Go cue activity (change in spike rate after the go cue; 100 ms window) in thal_ALM_ with or without ALM photoinhibition. The same format as in **E**. See also Figure S3.

We performed extracellular recordings in thal_ALM_ and compared responses to the Go cue (i.e. changes in spike rate after the Go cue) to those in ALM (Figures 3A, 3B, S3 and S5). Neurons in ALM and thal_ALM_ responded with increases or decreases in spike rate (go-up and go-down cells, respectively). The latency was shorter in thalamus (16.5 ± 1.5 ms; mean ± S.E.M.; time when 1% of cells show increase in spike rate) compared to ALM (25.0 ± 0.8 ms; mean ± S.E.M.) (*p* < 0.001; bootstrap; Figures 3A and 3B). The latency difference between thal_ALM_ and ALM is consistent with the action potential propagation speed in thalamocortical axons (Guo et al., 2017). Neurons with short latencies (< 20 ms) were widespread in thal_ALM_, and appeared to be partially spatially segregated from delay-selective neurons (Figure 3C; 5.4% of thal_ALM_ cells with delay selectivity had short latency) (Gaidica et al., 2018). We observed similar short latency activity in thal_ALM_ of mice performing an auditory delayed-response task (Figure S3) (Inagaki et al., 2018).

Photoinhibition of ALM reduced the activity of thal_ALM_ during the delay epoch (Guo et al., 2017) (Figures 3D, 3E, and S3E), but did not change the amplitude of the Go cue response in thal_ALM_, although the photoinhibition lasts until the response epoch (Figure 3F). Thus, ALM is not necessary for the Go cue response in thal_ALM_. Together with the latency analysis, these results indicate that the Go cue activity first arrives in thal_ALM_, then drives ALM (Dacre et al., 2021; Takahashi et al., 2021).

Auditory cortex neurons can respond to sounds with short latencies (12 ms; (Williamson and Polley, 2019)) but do not directly project to ALM (Guo et al., 2017; Oh et al., 2014). M1 (AP 0.15 mm, ML 1.7 mm from Bregma) and ALM are bidirectionally connected (Guo et al., 2017). M1 showed latencies similar to ALM (Figure S3; 20.6± 5.1 ms; mean ± S.E.M.; *p* = 0.41, Bootstrap). Because M1 is not necessary for initiation of directional licking (Xu et al., 2019), and because of slow propagation of intercortical signals between M1 and ALM (Guo et al., 2017), a parsimonious explanation is that the Go cue response in ALM does not rely on M1. Instead, thal_ALM_ is likely a source for the Go cue to ALM.

### Inputs to ALM-projecting thalamus

We injected retrograde tracers in thal_ALM_ (retrobeads and AAV_retro_, Figures S4A–S4C), which revealed inputs from ipsilateral frontal cortex and multiple subcortical areas, including the substantia nigra pars reticulata (SNr), superior colliculus (SC), deep cerebellar nuclei (DCN), and PPN/MRN (S4A-S4C) (Saper and Loewy, 1982; Krout et al., 2002; Martinez-Gonzalez et al., 2011; Guo et al., 2017; Gao et al., 2018).

**Figure 4.**
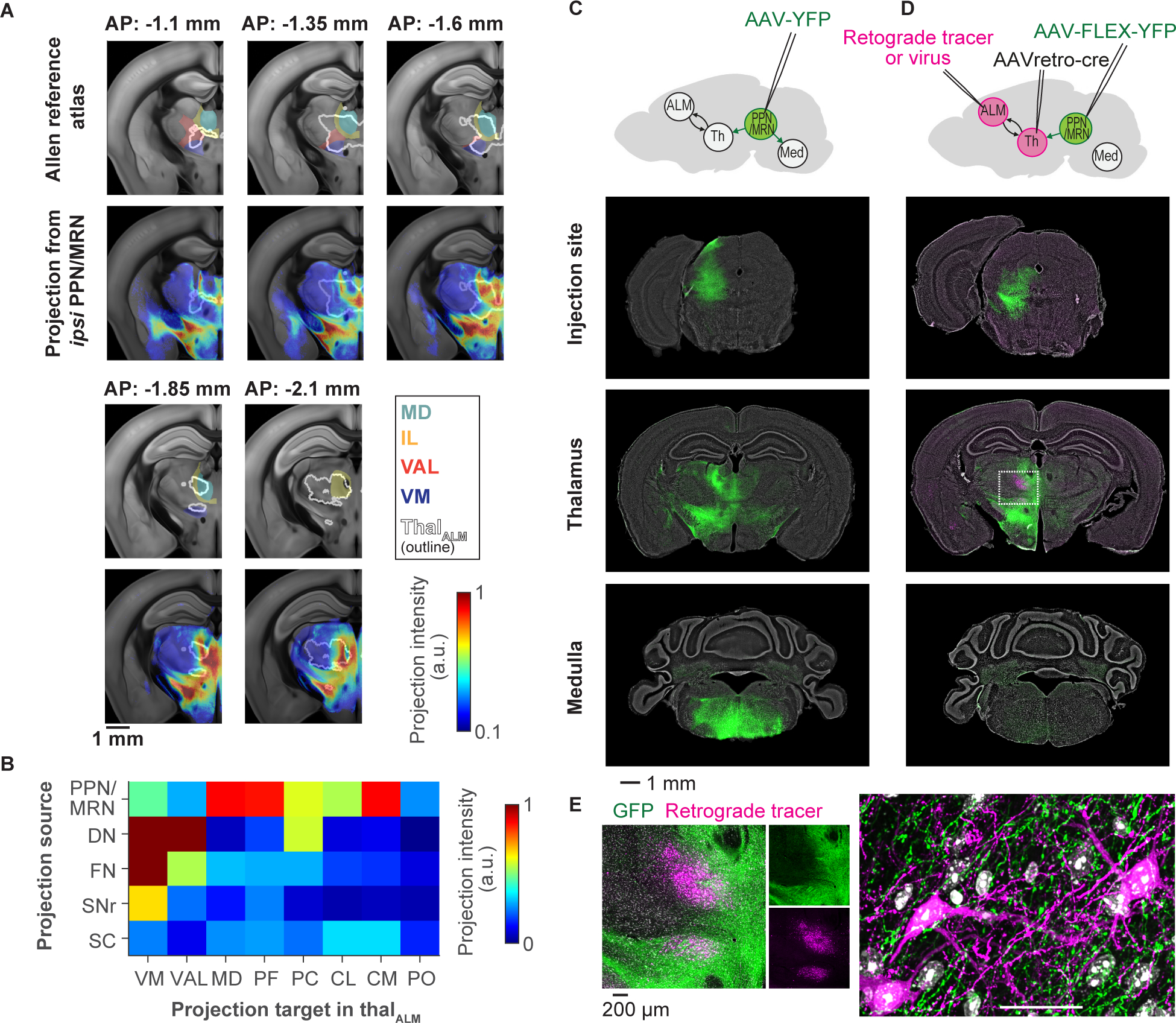
PPN/MRN projects to ALM-projecting thalamus A. Projections from ipsilateral PPN/MRN to thal_ALM_ (coronal view). Top, Allen Reference Atlas. Each colored region, a different thalamic nucleus. White contour, thal_ALM_. Bottom, the intensity of projection from PPN/MRN, registered to the Allen CCF (mean of 4 mice). AP, posterior to Bregma. B. Quantification of anterograde labeling in thal_ALM_ from subcortical structures (Methods). C. Top, labeling all PPN/MRN neurons. Med, medulla. Bottom, signal at the injection site, in the thalamus, and in the medulla. The image gains and contrasts are identical between the images of the thalamus and the medulla. Green, YFP; white, Nissl staining. D. Top, selective labeling of thalamus-projecting PPN/MRN neurons. Bottom, the same format as in **C**. Magenta, retrograde tracing from ALM. E. Enlarged image of the thalamus in **D**. F. Putative PPN/MRN connections onto thal_ALM_ neurons in VM. Red, retrograde labeling from ALM. Green, axonal terminals of PPN/MRN neurons. White, DAPI. Maximum intensity projection of a volume with 17.3 μm thickness. See also Figure S4.

To map the projections from these subcortical areas within thal_ALM_ and beyond we injected AAVs expressing fluorescent proteins in each area (Figures 4A and S4D; Table S2). PPN/MRN neurons have widespread projections to thal_ALM_, whereas other structures have relatively localized projections (Figure 4B). We focused on PPN/MRN because their output overlaps with the short latency Go cue responses in thal_ALM_ (Figure S4F).

Thalamus-projecting PPN/MRN (PPN/MRN_Th_) neurons are distributed across a region referred to as the ‘mesencephalic locomotor region’ (MLR) (Shik et al., 1966). Stimulation of the MLR produces locomotion, mediated by glutamatergic neurons that descend into the medulla (Shik et al., 1966; Roseberry et al., 2016; Josset et al., 2018; Caggiano et al., 2018). PPN/MRN neurons in mice project to both thalamus and the medulla (Figure 4C). To label PPN/MRN_Th_ neurons specifically, we injected AAV_retro_-Cre in thal_ALM_, and AAV-FLEX-YFP in PPN/MRN (Figure 4D). By injecting a retrograde tracer in ALM of the same animal, we confirmed that projections from PPN/MRN partially overlap with thal_ALM_ (Figure 4E and 4F). These experiments revealed that PPN/MRN_Th_ neurons lack a projection to the medulla, and thus constitute a distinct population, intermingled with neurons that project to the medulla (Figure 4D). PPN contains a high density of cholinergic (*chat*+) cells (Sofroniew et al., 1985; Mena-Segovia and Bolam, 2017; Huerta-Ocampo et al., 2020). However, the majority (75%) of PPN/MRN_Th_ neurons are glutamatergic (*vglut2*+), not cholinergic or GABAergic (*gad1*+), as confirmed by fluorescent *in situ* hybridization and immunohistochemical staining (Figures S4G–S4J; Table S4). Whole-cell patch-clamp recordings in acute brain slices confirmed direct glutamatergic input from PPN/MRN to thal_ALM_ neurons (Figures S4K–S4Q).

### Latency after the Go cue in thalamus-projecting brain areas

**Figure 5:**
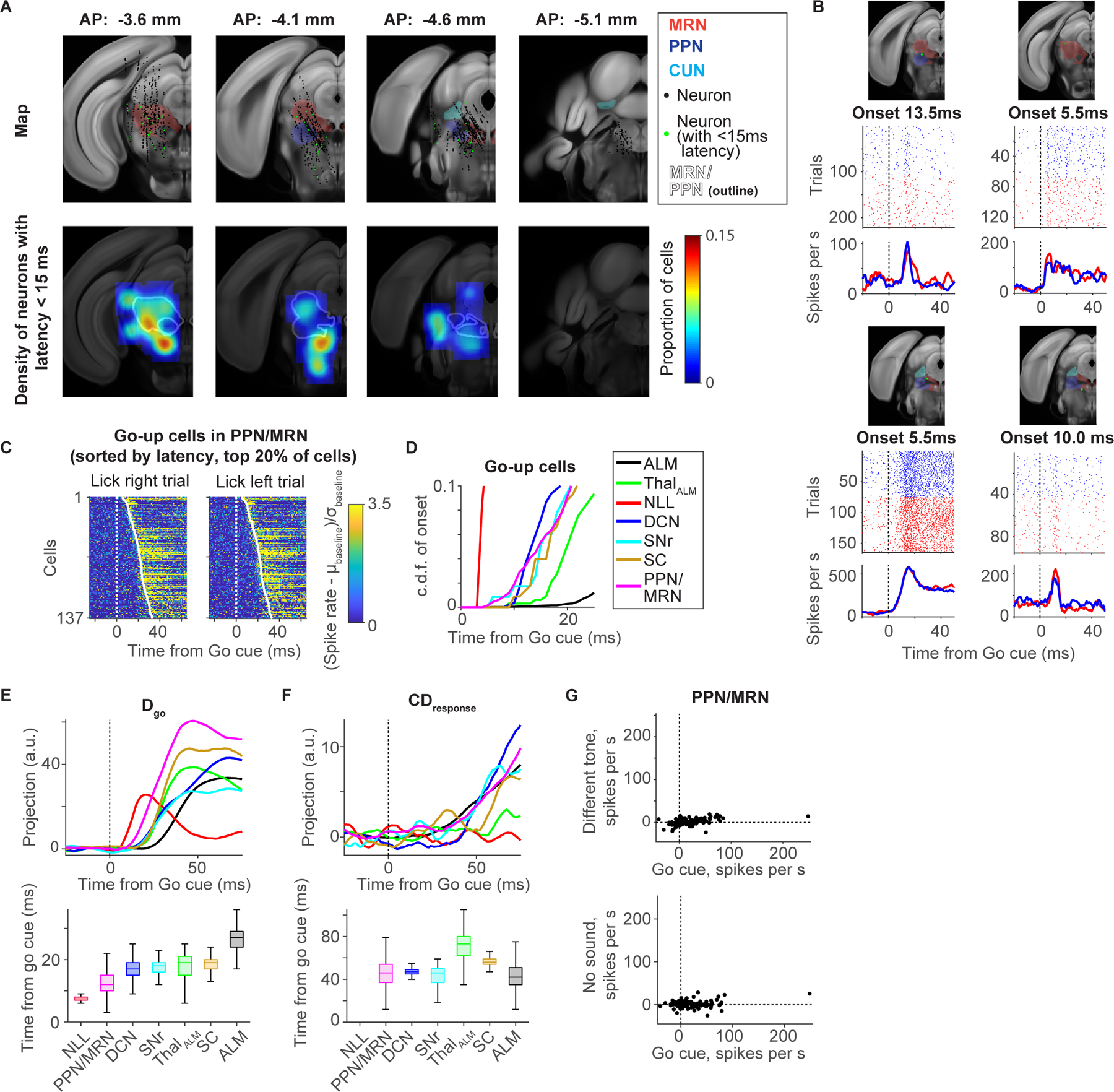
Short latency Go cue signals in PPN/MRN A. Top, recording sites in Allen CCF. Colored regions, different midbrain nuclei. Black dots, individual neurons. Green, neuron with <15 ms latency. Bottom, the density of neurons with < 15 ms latency. White contour, MRN and PPN. CUN, cuneiform nucleus. B. Example PPN/MRN neurons. Top, location of recorded neuron (green circle) in the Allen CCF. Middle, spike raster. Bottom, mean spike rate. C. Go cue response of PPN/MRN sorted by their latency. Spike rates are normalized by the baseline (spike rate before the Go cue, 100 ms window) for each neuron. D. C.d.f. of latency to the Go cue across neurons in each brain area (Methods). E. Top, projection of activity along **D**_**go**_ in each brain area. Bottom, the latency of post-Go cue increases in activity along **D**_**go**_ (Methods). Central line in the box plot, median. Top and bottom edges, 75% and 25% points. Whiskers, the lowest/highest datum within 1.5 interquartile range of the lower/upper quartile. The color indicates a different brain area as in **D**. F. Same as **E** for activity along **CD**_**response**_. G. Increase in spike rate of PPN/MRN neurons in response to the Go cue and different tone (top) or no sound at the expected timing of the Go cue (bottom). Circles, neurons (n = 178). See also Figure S5.

Next, we compared the latencies of Go cue responses across the subcortical areas projecting to thal_ALM_, including DCN (Gao et al., 2018), SNr (Guo et al., 2017), SC, and PPN/MRN (Figure 5A). We found neurons with fast Go cue responses (<15 ms) in multiple areas: the nucleus of lateral lemniscus (NLL), an auditory center that receives direct input from the cochlear nucleus (Davis et al., 1982); the pontine reticular nucleus (PRN), part of the acoustic startle pathway (Davis et al., 1982); the auditory thalamus (medial geniculate body, MGB) (Figure 5A; arrowheads; these brain areas do not project to thal_ALM_ directly). In addition, we found cells with short latency Go cue responses in PPN/MRN (white outline; Figures 5A–5C) (Reese et al., 1995; Dormont et al., 1998; Pan et al., 2005).

We compared the latencies and spike rates after the Go cue across brain areas (Figures 5D and S5). All thal_ALM_-projecting subcortical areas contain cells with Go cue latencies shorter than thal_ALM_. To analyze the latency of the non-selective component of the Go cue response, we projected activity in each area to its **D**_**go**_. Among the thal_ALM_-projecting areas, PPN/MRN is one of the first where **D**_**go**_ emerges after the Go cue (Figure 5E; *p* = 0.07, the probability that PPN/MRN has the shortest latency, hierarchical bootstrap). Unlike activity along **D**_**go**_, the selective component of the Go cue response, i.e. activity along **CD**_**response**_ in each brain area, emerged almost simultaneously across brain areas, and later than **D**_**go**_ (Figures 5F). Thus, following the Go cue, non-selective activity rapidly spreads across brain areas, followed by emergence of selective activity.

The fast Go cue responses in PPN/MRN are not simple auditory responses. Rather, they constitute a learned response that is specific to the sound used as the Go cue. We tested this by recording in mice trained with either 3 or 12kHz Go cue and trained to ignore the other tone (Figures 5G and S5G). The response does not reflect timing of the task because there was no response without the Go cue (Figure 5G). Responses in thal_ALM_ were also specific to the Go cue, with no response to the other tone, consistent with the view that PPN/MRN conveys Go cue signal to thal_ALM_ (Figure S5G–J).

Since PPN/MRN contains cells with short latencies (Figures 5D and 5E), and since their projections overlap with a subregion of thal_ALM_ containing fast Go cue responses (Figure S4F), we next tested whether activity in thalamus-projecting PPN/MRN is causal for the cue-triggered activity in ALM and movement initiation.

### Phasic stimulation of thalamus-projecting PPN/MRN neurons mimics the Go cue

**Figure 6:**
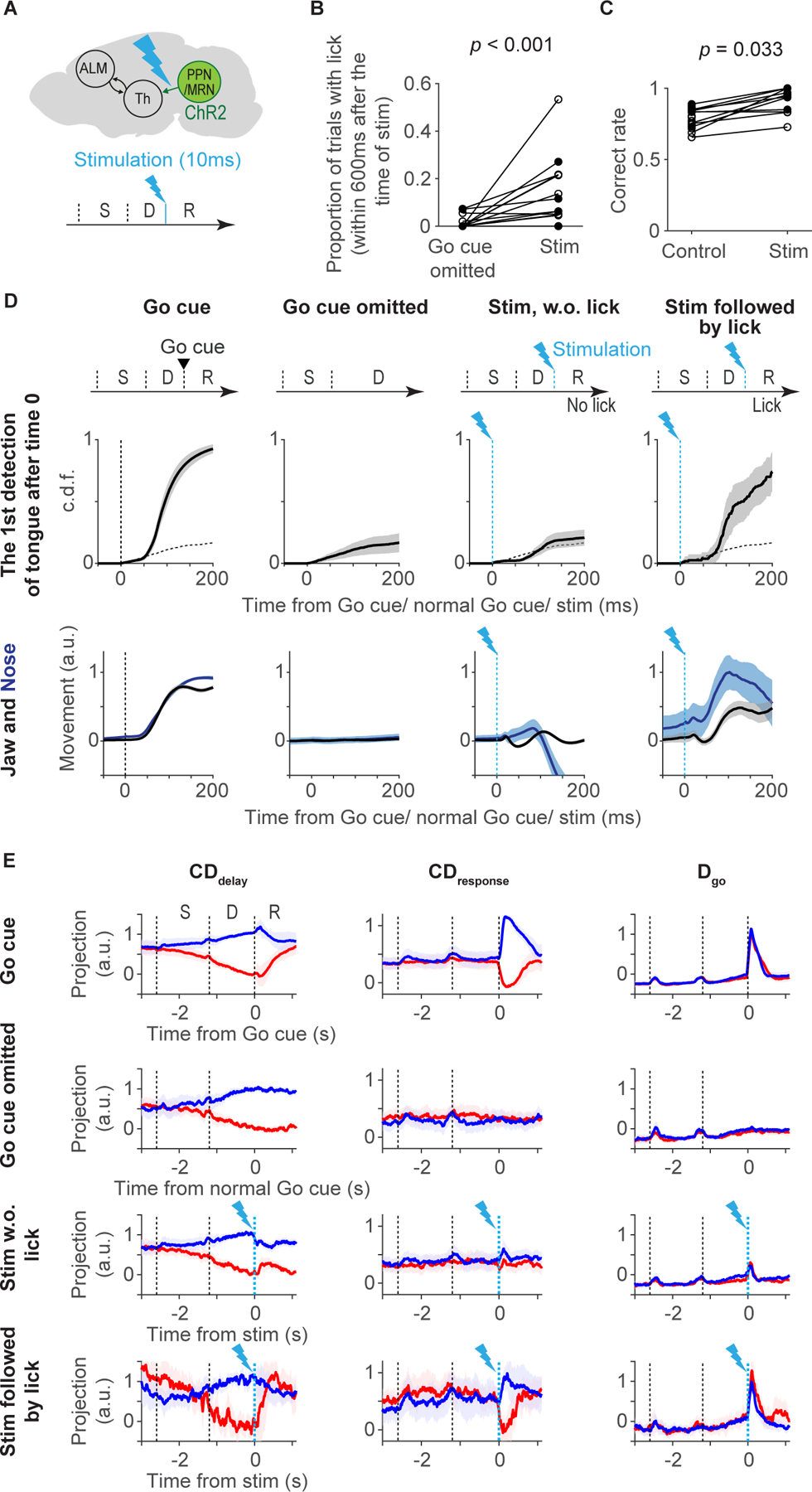
Stimulation of thalamus-projecting PPN/MRN neurons triggers planned movement A. Schema of PPN/MRN_Th_ stimulation experiment. B. Proportion of trials with lick after PPN/MRN_Th_ stimulation. Circle, mouse (n = 20). Filled circle, mice with unilateral virus injection (n = 4). *P-value*, hierarchical bootstrap with a null hypothesis that the proportion of trials with licks in stimulation trials are the same or lower than that in control. C. Same as **B** for the correct rate after the Go cue (control) or stimulation. D. Top, cumulative distribution of the first tongue detection after time 0 (Figure 1B). Dotted line, data in Go cue omitted condition for comparison. Bottom, jaw movement (black), and nose movement (blue). Trials are classified as follows: Go cue, trials with the Go cue; Go cue omitted, trials without the Go cue or stim; Stim w.o. lick, trials with stimulation but without lick; Stim followed by lick, trials with stimulation followed by lick. **E.** Projection of activity along **CD**_**delay**_ (left), **CD**_**response**_ (middle), and **D**_**go**_ (right) across trial types. Cyan dashed line, photo-stimulation. Line, grand median of sessions (n = 21 sessions; 12 mice); shading, S.E.M. (*hierarchical bootstrap*). See also Figure S6.

If PPN/MRN neurons signal the Go cue to ALM via thalamus, phasic optogenetic stimulation of PPN/MRN_Th_ neurons should trigger the effects of the Go cue. To stimulate PPN/MRN_Th_ neurons we injected ChR2-expressing AAV in PPN/MRN (unilaterally or bilaterally, n =20 mice) and placed a fiber optic unilaterally in thal_ALM_ for axonal photostimulation (Figures 6A and S6A–S6C) (Petreanu et al., 2007). Mimicking the phasic Go cue response with brief (5 or 10 ms) photostimulation of PPN/MRN axons increased licking responses (Figures 6B and S6H; *p* < 0.001, bootstrap; differences across animals are partially explained by the location of AAV infection and by the evoked activity patterns; Figures S6D–S6F). Importantly, when mice licked in response to photostimulation, they licked in the correct direction defined by the trial type (Figure 6C). This was the case even in mice with unilateral injection of ChR2-expressing AAV in PPN/MRN and ipsilateral photostimulation in thal_ALM_ (Figure S6G; n = 3 mice; note that PPN/MRN neurons project to ipsilateral Th, and more weakly to contralateral thalamus; Figure S4E). Triggering the movement to the instructed direction is precisely what the Go cue does: it does not carry directional information by itself, but releases planned movements. This property of PPN/MRN stimulation is unusual, as unilateral stimulation of motor-related cortex and midbrain generally drives contralateral movements (Li et al., 2015; Lee et al., 2020; Dacre et al., 2021; Lee and Sabatini, 2021), whereas stimulation of DCN results in ipsilateral movement (Gao et al., 2018).

In addition, photostimulation of PPN/MRN_Th_ neurons induced orofacial movements similar to those triggered by the Go cue (Figures 1B and 6D; Movie S2). Mice did not initiate movement by anticipating the Go cue timing (Figure 6D; Go cue omitted). In trials in which mice did not lick in response to photostimulation, orofacial movements were attenuated (Figure 6D; stim, w.o. lick).

We next compared ALM activity with Go cue-triggered licks and PPN/MRN_Th_ stimulation-triggered licks. The changes in activity produced by the Go cue and photostimulation were remarkably similar. In particular, the photostimuli did not merely excite all ALM neurons by increasing glutamatergic input from PPN/MRN_Th_ neurons. Instead, in trials in which mice licked in response to photostimulation, neural activity in ALM resembled activity triggered by the Go cue at the level of individual cells (Figure S6I) and at the population level (Figures S6J and S6K) by both increasing and decreasing spike rates (Dacre et al., 2021).

To analyze the population activity pattern we projected the population activity along **CD**_**delay**_, **CD**_**response**_, and **D**_**go**_. The activity underlying stimulation-triggered licks was similar to that underlying cue-triggered licks. Namely, we observed a significant change in activity along **D**_**go**_ and **CD**_**response**_ (Figures 6E and S6L). Furthermore, activity along **CD**_**delay**_ collapsed after the photostimulation, similar to changes observed after the Go cue (Figure 6E). These changes happen before the onset of movement (within 50 ms after the onset of stimulation; Figures S6L and S6M), implying that they are not caused by the movement. In trials in which mice did not lick in response to photostimulation, changes in activity were much attenuated, consistent with the reduced orofacial movements (Figures 6D and 6E). No activity change was observed in Go cue omitted trials consistent with the lack of movement.

The amplitude of activity along **D**_**go**_ predicted whether photostimulation triggered licks on a trial-by-trial basis (Figure S6N; *p* < 0.05 in 4 out of 7 sessions). Activity along **CD**_**response**_ is determined by both activity along **D**_**go**_ and **CD**_**delay**_ (Figures S6O and S6P). Altogether, when photostimuli induced sufficiently large activity along **D**_**go**_ they create activity along **CD**_**response**_ in a manner proportional to the activity along **CD**_**delay**_, which results in licking in the planned direction.

### Perturbation of thalamus-projecting PPN/MRN neurons blocks movement initiation

We next tested if PPN/MRN activity is necessary for cue-triggered movement initiation. First, infusion of the GABA_A_ receptor agonist muscimol bilaterally (2.5–5ng) in PPN/MRN prevented mice from licking (Figures S7A and S7B). To test whether PPN/MRN is required for the Go cue response in ALM, we performed extracellular recordings of ALM during the muscimol infusion. Similar to licking, the Go cue response decreased rapidly after infusion (Figures S7C–H). Thus, PPN/MRN is required for the Go cue response in ALM and licking.

**Figure 7:**
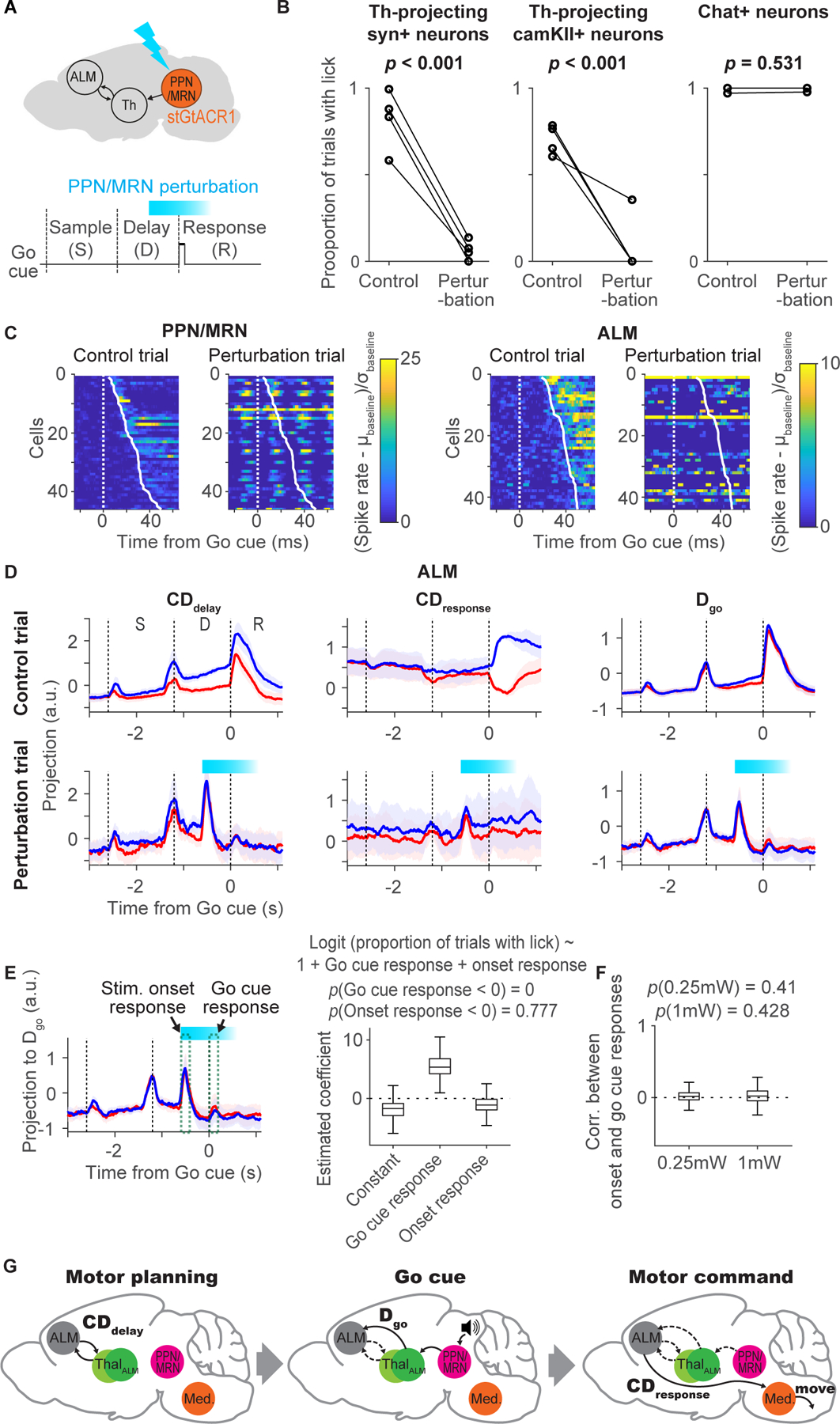
Perturbing thalamus-projecting PPN/MRN neurons blocks planned movement A. Schema of PPN/MRN_Th_ perturbation experiment. B. Behavioral effects of perturbing Th-projecting Syn+ neurons (left; n = 4 mice), Th-projecting CamKII+ neurons (middle; n = 4 mice), and Chat+ neurons (right; n = 2 mice; note Chat+ cells are not necessarily projecting to thalamus) in PPN/MRN. *P-value*, hierarchical bootstrap with a null hypothesis that the proportion of trials with licks in perturbation trials are the same or higher than that in control. C. Go cue response sorted by their latency. Neurons with increase in spike rate (within 50 ms after the Go cue) are shown (45/292 cells in PPN/MRN and 44/635 cells in ALM). Activities of the same neurons in control (left) and perturbation (right) trials. Spike rates are normalized by baseline (spike rate before the Go cue in control trials, 100 ms window). **C**-**F**, results of perturbing Th-projecting CamKII+ neurons. D. Projection of activity along **CD**_**delay**_, **CD**_**response**_, and **D**_**go**_ across trial types. Cyan, laser on. Line, grand median of sessions (n = 17 sessions; 4 mice); shading, S.E.M. (*hierarchical bootstrap*). E. Left, schema of activity analyzed in the regression analysis. Mean activity within the green dotted lines were analyzed (window size, 200 ms). Right, estimated coefficients of logit regression. *P-value*, hierarchical bootstrap (n = 17 sessions; 4 mice). F. Correlation between activity along **D**_**go**_ at the Go cue and at the stim onset. *P-value*, hierarchical bootstrap with a null-hypothesis that coefficient is lower than 0 (n = 17 sessions; 4 mice). G. Multi-regional flow of information underlying the cue-triggered movement initiation. Left, preparatory activity (**CD**_**delay**_) is maintained in a cortico-thalamocortical loop. Middle, the Go cue (speaker) activates PPN/MRN, which then activates neurons in thal_ALM_, which are different from neurons that maintain preparatory activity (green circles). This induces activity along **D**_**go**_ in ALM. Right, the **D**_**go**_ activity then causes a collapse of activity along **CD**_**delay**_ and an emergence of motor command (**CD**_**response**_), which engages medulla (Med.) circuits to initiate planned movements. See also Figure S7 and S8.

To perturb PPN/MRN_Th_ neurons in a temporally precise manner, we expressed stGtACR1 in PPN/MRN_Th_ neurons bilaterally by injecting AAV_retro_-yn-Cre in thalamus and AAV-FLEX-stGtACR1 in PPN/MRN (Figures 7A, S8A and S8B). Optogenetic perturbation during the Go cue presentation blocked cue-triggered licks (Figure 7B; laser was turned on 0.6 s before the Go cue and lasted for 1.2 s; 473 nm 40 Hz sinusoidal). Perturbation of glutamatergic PPN/MRN_Th_ neurons (using AAV_retro_-CamKII-Cre; Figure S4H) resulted in a similar behavioral effect (Figure 7B; in contrast, perturbation of cholinergic neurons did not affect movement initiation). Even after the end of the perturbation, mice showed reduced licks (Figure S8C). Thus, unlike PT_lower_ silencing in which mice licked after silencing (Figure 2B), mice behaved as if there was no Go cue.

To measure the effect of the optogenetic manipulation, we performed extracellular recordings in mice expressing stGtACR1 in CamKII+ PPN/MRN_Th_ neurons. A subset of PPN/MRN neurons showed increases in spike rate time-locked to the sinusoidal laser modulation (23/45 cells; *p* < 0.05, ranksum test; analyzed cells with latency to the Go cue shorter than 50 ms), likely due to residual axonal excitation caused by stGtACR1 (Figures 7C, S2H and S8D) (Mahn et al., 2018; Messier et al., 2018). Indeed, increases in spike rate occurred around and slightly after the peak of the laser power (Figure S8F). Other PPN/MRN neurons (13/45; *p* < 0.05, ranksum test) were silenced (Figures 7C and S8D). In both cases, the Go cue response was abolished in perturbation trials (Figures 7C and S8D).

Next, we performed extracellular recordings in ALM. Like in PPN/MRN, the Go cue response was eliminated in individual ALM neurons during the perturbation (Figures 7C and S8E). The sinusoidal modulation was attenuated in ALM (Figure S8F). ALM neurons showed a loss of Go cue response when Syn+ or CamKII+ PPN/MRN_Th_ neurons were perturbed, but not when Chat+ PPN/MRN neurons were perturbed, consistent with the behavioral effect (Figure S8G).

In activity space, the Go cue response disappeared across all directions (Figures 7D and S8I). In addition to the loss of the Go cue response, the photostimulation onset caused a transient onset response, which was likely induced by stGtACR1-dependent axonal excitation (Figure 7E) (Mahn et al., 2018; Messier et al., 2018). This excitation did not trigger licks, presumably because the temporal patterns of excitation are different (e.g., much briefer) compared to activity after the Go cue. A question is whether this transient excitation precludes ALM from responding to the Go cue. We addressed this question in three ways. First, logistic regression showed that larger attenuation of the Go cue response corresponds to lower probability of licking (Figure 7E). Second, the amplitude of the onset response was not correlated with the loss of the Go cue response (Figures 7F and S8H). Third, presenting a Go cue in the middle of the delay epoch does not preclude mice and ALM activity from responding to another Go cue presented at the end of the delay epoch (Figures S7I–K). Altogether, a parsimonious explanation is that the perturbation of the Go cue response in PPN/MRN results in a loss of Go cue response in ALM and a loss of movement initiation.

## Discussion

ALM activity can be decomposed into several activity modes that capture a large proportion of cortical activity (Figure 1) (Li et al., 2016; Inagaki et al., 2018). During the delay epoch, movement-selective preparatory activity is contained mostly in the **CD**_**delay**_ mode in activity space. After the Go cue, this activity rapidly reorganizes into the non-selective **D**_**go**_ mode and the direction-selective **CD**_**response**_ mode. This progression underlies movement initiation.

We identified a multi-regional neural pathway that is critical for reorganizing ALM activity in response to the Go cue and to initiate planned directional licking (Figure 7G). Ascending glutamatergic neurons in PPN/MRN signal the Go cue information to ALM via thal_ALM_ and thereby cause the reorganization of ALM activity and release planned movements. Our conclusions are based on multiple lines of evidence. First, PPN/MRN contains neurons that specifically respond to the auditory Go cue (and not to other sounds) (Figure 5G). Second, latencies after the Go cue are shorter in PPN/MRN than in thal_ALM_ (Figure 5E). Third, PPN/MRN neurons project to areas of thal_ALM_ that show short-latency Go cue responses (Figure 4). Fourth, brief optogenetic stimulation of PPN/MRN_Th_ neurons triggers rapid changes in ALM activity, similar to those caused by the Go cue itself (Figure 6E). Fifth, optogenetic stimulation of PPN/MRN_Th_ neurons elicited the appropriate directional licking, even after unilateral stimulation of PPN/MRN_Th_ neurons (Figure 6C), unlike other thal_ALM_ projecting areas. Sixth, perturbation of PPN/MRN_Th_ activity resulted in a loss of Go cue response in ALM and abolished behavioral responses (Figures 7, 8 and S7). Our findings are consistent with previous experiments in rats, cats and monkeys, in which lesioning or silencing of PPN/MRN blocks cue-triggered movements (Wilson, 1973; Condé et al., 1998; Florio et al., 1999). In humans, PPN/MRN and downstream thalamic areas show activity in cued movement tasks (Kinomura et al., 1996).

PPN/MRN receives input from NLL (Reese et al., 1995), which itself receives direct auditory input from the cochlear nucleus (Davis et al., 1982). As a result the latency to the Go cue in NLL is shorter than in PPN/MRN (Figure 5). The auditory response in PPN/MRN is specific to the sound associated as the Go cue (Figure 5G and S5G–J). PPN/MRN is likely the site of this association. PPN/MRN also responds to other sensory stimuli (Pan et al., 2005; Okada and Kobayashi, 2009) and may serve to associate sensory stimuli as a “Go” signal.

Most of our understanding of thalamocortical processing is based on sensory thalamus and cortex. Less is known about non-sensory (‘higher-order’) thalamus and its interactions with the frontal cortex. Cortex and higher-order thalamus are coupled in both directions (Bolkan et al., 2017; Guo et al., 2017; Schmitt et al., 2017). Our experiments suggest that midbrain sends simple contextual signals to the cortex via particular thalamic nuclei to modulate cortical activity modes. Different thalamic nuclei contain neurons with different projection patterns (Steriade et al., 1997; Clascá et al., 2012). For example, VM contains neurons that have broad projection patterns to layer 1 (‘matrix’). VAL instead projects in a more focal manner to middle layers (‘core’) (Jones, 1998; Kuramoto et al., 2015). These different thalamocortical projections activate cortical microcircuits in specific ways (Anastasiades et al., 2020). The spatial distribution of thal_ALM_ neurons with short latencies to the Go cue appears to differ from those showing delay selectivity (Figure 3C). It will be interesting to learn how the thalamic nuclei showing different activity patterns modulate cortex through their specific thalamocortical projections. We note that the PPN/MRN input to thal_ALM_ produces short-latency and reliable synaptic currents with paired-pulse depression (Figure S4P and S4Q). Thus, the PPN/MRN_Th_ → thal_ALM_ projection has the hallmarks of a classic driver input (Guillery and Sherman, 2002).

Animal behavior often consists of multiple phases, each corresponding to different computations. Activity modes underlying each phase likely occupy near-orthogonal activity subspaces so that the different computations do not interfere with each other (Mante et al., 2013; Kaufman et al., 2014; Stavisky et al., 2017; Hennig et al., 2018; Rouse and Schieber, 2018). However, the information carried by a mode needs to be transferred to subsequent modes to mediate coherent behavior. For example, information encoded by the preparatory activity must be transferred to the motor command to initiate planned movement. Indeed, we observed correlation between activity along **CD**_**delay**_ during the delay epoch and **CD**_**response**_ after the Go cue (Figures S1L and S6P).

We designed our optogenetic manipulations to test the causal roles of each activity mode. Silencing PT_lower_ neurons resulted in a loss of movements and reduced activity along **CD**_**response**_, without affecting **D**_**go**_ (Figure 2G). Optogenetic stimulation of PPN/MRN_Th_ axons first increased activity along the **D**_**go**_. When **D**_**go**_ activity was sufficiently large it also induced selective activity along **CD**_**response**_ and triggered appropriate behavioral responses (Figures 6E and S6O). Thus, although both **CD**_**response**_ and **D**_**go**_ emerge after the Go cue, they are dissociable (Figures 2 and 6): activity along **D**_**go**_ is not sufficient to trigger movement by itself. Instead, it induces activity along **CD**_**response**_, which then presumably controls the movement via PT_lower_ cells.

Our analysis relies on the millisecond temporal precision provided by electrophysiology and behavioral tracking with high-speed video, as well as a behavioral task with multiple choices. Our results provide a clear demonstration that state space analysis can extract features of population activity that have specific roles in behavior.

Parkinson’s disease (PD) patients experience difficulty in self-initiating movement, clinically described as freezing of gait (FOG). However, they can often perform complex movements in response to sensory cues, such as catching a ball. This phenomenon, known as paradoxical kinesis, is commonly used for rehabilitation (Ginis et al., 2018). Neurodegeneration in PD impacts activity in basal ganglia, a structure important for motor control (DeLong, 1990; Gerfen and Surmeier, 2011; Hikosaka et al., 2000; Klaus et al., 2019; Mink, 1996). PPN/MRN_Th_ → thal_ALM_ → MCx (e.g. ALM) pathway may initiate movement bypassing basal ganglia (Schwab et al., 2020), which could explain why cue-triggered movement is spared in PD patients. In addition, deep brain stimulation (DBS) of PPN has been applied to treat the FOG in PD (Thevathasan and Moro, 2019). The PPN DBS improves simple reaction tasks (Thevathasan and Moro, 2019), raising a possibility that PPN DBS is acting on the cue-triggered movement initiation mechanism. Further investigation on genetically-defined cell types and precise location of PPN/MRN neurons that underlie cue-triggered movement may help to optimize treatment of PD.

### Limitation of the study

Here we emphasized the PPN/MRN_Th_ → thal_ALM_ → ALM circuit. However, PPN/MRN neurons likely exert their role in movement initiation via additional brain regions. For example, PPN/MRN_Th_ neurons also project to the SNr and subthalamic nucleus (STN) (Figure 4A) (Martinez-Gonzalez et al., 2011; Vitale et al., 2019; Ferreira-Pinto et al., 2021). STN in turn projects to SNr, and SNr is known to control premotor circuits in the superior colliculus and medulla (DeLong, 1990; Hikosaka et al., 2000; Klaus et al., 2019). In addition, PPN/MRN_Th_ neurons may locally excite or inhibit PPN/MRN neurons that descend to motor centers. Indeed, PPN/MRN is ideally positioned to coherently control brain-wide circuits for movement initiation.

Our experiments do not exclude contributions from additional subcortical areas to movement initiation, such as DCN (Spidalieri et al., 1983; Gemba and Sasaki, 1987; Gao et al., 2018; Dacre et al., 2021) and basal ganglia (da Silva et al., 2018; Díaz-Hernández et al., 2018). Unilateral optogenetic stimulation of DCN in the delayed-response task biased lick direction but did not trigger licks (Gao et al., 2018). Yet, in a cued reaching task, DCN activity is causal for the movement initiation and activity in MCx (Dacre et al., 2021). It remains to be seen whether DCN can serve a similar role as PPN/MRN in terms of switching modes of cortical activity. PPN/MRN and DCN project to partially overlapping thalamic nuclei. Further investigation is required to test whether PPN/MRN and DCN are redundant, serve as parts of a chain or loop (Hazrati and Parent, 1992; Bostan and Strick, 2018; Judd et al., 2021) or as parallel Go cue pathways for different sectors of MCx, or are recruited differently depending on task requirements.

### RESOURCE AVAILABILITY

#### Lead contact

- Further information and requests for resources and reagents should be directed to and will be fulfilled by the lead contact, Hidehiko K. Inagaki (hidehiko.inagaki@mpfi.org).

#### Materials availability

- This study did not generate new unique reagents.
- This study did not generate new mouse lines.

#### Data and code availability

- Recording data with example codes will be deposited at Dandiarchive.org. Accession numbers are listed in the key resources table.
- This paper does not report the original code.
- Any additional information required to reanalyse the data reported in this work paper is available from the Lead Contact upon request.

### EXPERIMENTAL MODEL AND SUBJECT DETAILS

#### Mice

This study is based on both male and female mice (age > P60, except for acute slice recording). We used eight mouse lines: C57Bl/6J (JAX #000664), VGAT-ChR2-EYFP (JAX #14548) (Zhao et al., 2011), PV-IRES-Cre (JAX #017320) (Hippenmeyer et al., 2005), ChAT-IRES-Cre (JAX #006410) (Rossi et al., 2011), Vglut2-IRES-Cre mice (JAX #028863) (Vong et al., 2011), Sst-IRES-Cre (JAX #013044) (Taniguchi et al., 2011), Rbp4-Cre MMRRC031125 (Gerfen et al., 2013), and Ai32 (JAX #024109) (Madisen et al., 2012). See Supplementary Table 1 and 2 for mice used in each experiment.

All procedures were in accordance with protocols approved by the Janelia Institutional Animal Care, MPFI IACUC committee, and Use Committee or the University of Queensland Animal Ethics Committee. Detailed information on water restriction, surgical procedures, and behavior have been published (Guo et al., 2014a). Mice were housed in a 12:12 reverse light: dark cycle and behaviorally tested during the dark phase. A typical behavioral session lasted between 1 and 2 hours. Mice obtained all of their water in the behavior apparatus (approximately 1 ml per day; 0.3 ml was supplemented if mice drank less than 0.5 ml). Mice were implanted with a titanium headpost and single housed. For cortical photoinhibition, mice were implanted with a clear skull cap (Guo et al., 2014c). Craniotomies for recording were made after behavioral training.

### METHOD DETAILS

#### Virus and tracer injection

We followed published protocols (dx.doi.org/10.17504/protocols.io.bctxiwpn) for virus and tracer injection. See Supplementary Table 1 and 2 for detailed descriptions of viruses and injection coordinates. We used the following tracers: WGA-Alexa555 (WGA-Alexa Fluor® 555; Thermo Fisher Scientific) and Red RetroBeads (Lumafluor). See Supplementary Table 3 for a list of viruses used in this research.

#### Behavior

For the tactile delayed-response task (Guo et al., 2014a) (all experiments but Figures S3A–S3D and S7), at the beginning of each trial, a metal pole (diameter, 0.9 mm) moved within reach of the whiskers (0.2 s travel time) for 1.0 seconds, after which it was retracted (0.2 s retraction time). The sample epoch (1.4 s total) was the time from the onset of pole movement to the completion of pole retraction. The delay epoch lasted for another 1.2 s after the completion of pole retraction. An auditory ‘Go cue’ (pure tone, 3 or 3.4 kHz, 0.1 s) separated the delay and the response epochs.

A two-spout lickport (4.5 mm between spouts) was used to record licking events and deliver water rewards. After the Go cue, licking the correct lickport produced a water reward (approximately 2 μL); licking the incorrect lickport triggered a timeout (0 to 5 s). Licking before the Go cue (‘early lick’ trials) was punished by a timeout (1 s). Trials in which mice did not lick within 1.5 seconds after the Go cue (‘no response’ trials) were rare and typically occurred at the end of behavioral sessions.

For Figure 5G, individual mice were trained to respond to a 3 (or 12) kHz (pure tone, 0.1 s) Go cue, but to ignore another pure tone (12 or 3 kHz), played at the time of normal Go cue (different tone trials). Go cue omitted trials (trials without Go cue) and different tone trials were deployed in 25% randomly selected trials. Licking in Go cue omitted trials and different tone trials was punished with a timeout (1 s).

For the auditory delayed-response task (Figures S3A–D and S7), tones were presented at one of two frequencies: 3 or 12 kHz, during the sample epoch. Each tone was played three times for 150 ms with 100 ms inter-tone intervals. The following delay epoch lasted for another 1.2 seconds. An auditory ‘Go cue’ (carrier frequency 6 kHz, with 360 Hz modulating frequency, 0.1 s) separated the delay and the response epochs. For the task with a fake cue (Figure S7I–K), the Go cue sound was played 0.6s after the onset of the delay epoch. Licks after the fake cue were not rewarded or punished.

#### Optogenetics

Photostimulation was deployed on ~25% trials selected at random. To prevent mice from distinguishing photostimulation trials from control trials using visual cues, a ‘masking flash’ (1 ms pulses at 10 Hz) was delivered using 470 nm LEDs (Luxeon Star) throughout the trial. For both ChR2 and stGtACR1, we used a 473 nm laser (Laser Quantum). The laser power was controlled by an acousto optical modulator (AOM; Quanta Tech) and a shutter (Vincent Associates). See Supplementary Table 1.

The ChR2-assisted photoinihibition of ALM (Figures 3D–3F) was performed through clear-skull cap (beam diameter at the skull: 400 μm at 4 σ) (Guo et al., 2014c). We stimulated parvalbumin-positive interneurons in PV-IRES-Cre mice crossed to Ai32 reporter mice expressing ChR2 (Guo et al., 2017) for 1.6 s starting at the onset of the delay epoch (T_delay_) with 200 ms ramping down (mean laser power: 1.5mW). We silenced ALM ipsilateral to the recorded thalamus.

To silence medulla-projecting ALM neurons (PT_lower_) bilaterally (Figure 2), we photoinhibited for 1 s with 100 ms ramping down, starting at the timing of the Go cue. We photoinhibited four spots on each hemisphere, centered on ALM (AP 2.5 mm; ML 1.5 mm) with 1 mm spacing (in total eight spots bilaterally) using scanning Galvo mirrors through clear-skull cap. We photoinhibited each spot sequentially, at the rate of 5 ms per spot. The laser powers noted in the figures and text indicate the mean laser power per spot.

For PPN/MRN_Th_ axonal photostimulation experiments (Figure 6), we randomly interleaved three trial types: (1) Go cue trials, trials with Go cue at T_delay_ + 1.2 s (i.e. 1.2 s after delay onset; this is the control condition mice were trained with, which constitutes 75–85% of trials during the experiments); (2) Go cue omitted trials, trials without Go cue at T_delay_ + 1.2 s; (3) stimulation trials, trials with axonal excitation of Thalamus-projecting PPN/MRN by 20mW 473nm laser at T_delay_ + 1.2 s for 5 or 10 ms (through N.A. 0.37 fiber optics; see Supplementary Table 1 for coordinates). In both Go cue omitted trials and stimulation trials, a delayed Go cue was presented at T_delay_ + 2.4 s and licks to this Go cue were rewarded in order to maintain behavioral performance.

To prevent mice from associating optogenetic stimulation with water reward (and increasing licks because of this association), we did not provide water reward to stimulation-triggered licks (licks after the stimulation and before the delayed Go cue). Consequently, mice decreased stimulation-triggered licks over trials/sessions, presumably by learning to distinguish stimulation and the actual Go cue (Figure S6H).

For PPN/MRN_Th_ perturbations using stGtACR1 (Figure 7), we delivered photostimuli bilaterally to PPN/MRN_Th_ starting at T_delay_ + 0.6 s lasting 1.2 s duration (with 200 ms ramp up and down to minimize axonal excitation, 40Hz sinusoidal modulation; Go cue was presented at T_delay_ + 1.2 s). We tested 0.25, 1, and 10 mW photostimuli. The strongest photostimulus triggered axonal excitation, and was excluded from the analyses (Li et al., 2019).

#### Muscimol Infusion

Guide cannulas (26 Gauge, P1 Technologies) were implanted bilaterally during the head bar surgery. Internal cannulas (33 Gauge, P1 Technologies) projecting 1.5mm beyond the guide cannula tips were inserted just before infusion in mice performing the auditory delayed-response task (after 257 ± 47 trials, mean ± std). Muscimol hydrobromide (Sigma Aldrich) was dissolved in cortex buffer (NaCl 125 mM, KCl 5 mM, Glucose 10 mM, HEPES 10 mM, CaCl_2_ 2 mM, MgSO_4_ 2 mM, pH 7.4). The control solution was cortex buffer without muscimol. In all conditions, 50 nl solution was infused per hemisphere using Hamilton syringes. Behavior was initiated five minutes after the infusion. We focused our analysis on the first 20 trials (154 ± 26 seconds, mean ± std.) after the infusion (Figure S7B–E) to avoid side effects caused by the lack of licking and diffusion of muscimol. See Supplementary Table 1.

#### In vivo whole-cell recording

All whole-cell recordings were made from the left ALM. Data and detailed procedures have been published (Guo et al., 2017). In brief, we partially compensated for series resistance and injected a ramping current until action potentials disappeared (Anderson et al., 2000; Yu et al., 2016) (767 ± 172 pA for positive current injection, −164 ± 64 pA for negative current injection; mean ± standard deviation).

The principle of this experiment is as following. Neglecting spatial components, the membrane potential of ALM neurons is governed by:

$$C_{m}\frac{dV(t)}{dt}=-g_{L}(V)\left(V(t)-E_{L}\right)-g_{E}(t)\left(V(t)-E_{E}\right)-g_{I}(t)\left(V(t)-E_{I}\right)+I_{inj}(t)$$

g_L_, g_E_, and g_I_ are conductances related to leak, excitatory, and inhibitory currents, respectively. E_L_, E_E_, and E_I_ are the corresponding reversal potentials. g_L_ is a function of membrane potential because of intrinsic voltage-dependent currents.

We assume E_L_ = −50 mV, E_E_ = 0 mV and E_I_ = −70 mV. To selectively expose inhibitory conductances, we depolarized V near 0 mV. The contribution of g_E_ to the membrane potential is reduced because V(t) – E_E_ is near 0. On the other hand, the contributions of g_I_ to the membrane potential become stronger, since V(t) – E_I_ = 70 mV is larger (approximately 3.5 fold) compared to resting conditions (V(t) – E_I_ = 20mV). Increases or decreases in g_I_ result in hyperpolarization or depolarization, respectively. Similarly, for negative current injection experiments, we hyperpolarized V near −70 mV. Under this condition, the contributions of g_E_ to the membrane potential is increased. Increases or decreases in g_E_ result in depolarization or hyperpolarization, respectively.

#### Extracellular electrophysiology

A small craniotomy (diameter, 0.5 – 1 mm) was made over the recording sites one the day prior to the first recording session. Extracellular spikes were recorded using Janelia silicon probes (HH-2) with two shanks (32 channels each, 25 μm interval between channel, 250 μm between shanks), or Neuropixels probes (Jun et al., 2017b; Liu et al., 2021; Steinmetz et al., 2021). For the HH-2 probe, 64 channel voltage signals were multiplexed, recorded on a PCI6133 board (National instrument), and digitized at 400 kHz (14 bit). The signals were demultiplexed into 64 voltage traces sampled at 25 kHz and stored for offline analysis. All recordings were made with open-source software SpikeGLX (http://billkarsh.github.io/SpikeGLX/). During recordings the craniotomy was immersed in cortex buffer. Brain tissue was allowed to settle for at least five minutes before recordings. For ALM, recording depth (between 800 μm to 1100 μm) was inferred from manipulator readings. For subcortical areas, electrode tracks labelled with CM-DiI were used to determine recording locations (Liu et al., 2020) (Figure S3F).

#### Histology

Mice were perfused transcardially with PBS followed by 4% PFA / 0.1 M PB. Brains were post fixed overnight and transferred to 20% sucrose PB before sectioning on a freezing microtome. Coronal 50 μm free-floating sections were processed using standard fluorescent immunohistochemical techniques. All sections were stained with NeuroTrace® 435/455 Blue Fluorescent Nissl Stain (Thermo Fisher Scientific, N21479). The fluorescent label was amplified with immunohistochemical techniques with rabbit anti-RFP (Rockland Immunochemicals, Pottsdown, PA, 600–401-3790) and goat anti-rabbit 555 secondary antibodies (ThermoFisher Scientific, A27039) or chicken anti-GFP (Thermo Fisher Scientific, A10262) and goat anti-chicken 488 (Thermo Fisher Scientific, A11039). Cholinergic neurons were labeled with a mouse monoclonal antibody to ChAT (Sigma, AMAb 91130) and goat anti-mouse 647 secondary antibodies (Thermo Fisher Scientific, A11039). Slide-mounted sections were imaged on a Zeiss microscope with a Ludl motorized stage controlled with Neurolucida software (MBF Bioscience). Imaging was done with a 10× objective and a Hamamatsu Orca Flash 4 camera. The image in Figure 4F was acquired with a spinning-disk confocal system (Marianas; 3I, Inc.) consisting of a Axio Observer Z1 (Carl Zeiss) equipped with a CSU-W1 spinning-disk head (Yokogawa Corporation of America), ORCA-Flash4.0 v2 sCMOS camera (Hamamatsu Photonics), 63× 1.4 NA / Plan-Apochromat / 180μm WD oil objective. Image acquisition was performed using SlideBook 6.0 (3I, Inc).

#### Acute slice recording

AAV5-EF1α-DIO-hChR2 (H134R)-mCherry (University of Pennsylvania Vector Core) or AAV2/8-EF1α-DIO-hChR2 (H134R)-EYFP (Addgene) was injected in Vglut2-IRES-Cre mice at coordinates from Bregma (in mm); AP −4.7, ML 1.2 and −1.2, DV −3.5 in mice (P21-P28). Mice were used for slice recordings 4–8 weeks after the viral surgery. Animals were deeply anesthetized with isoflurane, perfused transcardially with ice-cold cutting solution containing (in mM): 87 NaCl, 50 sucrose, 25 glucose, 25 NaHCO3, 2.5 KCl, 4 MgCl2, 0.5 CaCl2, and 1.2 NaH2PO4, osmolarity 300–310 mOsm/kg) and subsequently decapitated. Brains were rapidly removed, and 300 μm thick coronal brain slices (Leica VT1000S vibratome, Germany) were prepared in chilled cutting solution, after which the slices were transferred to oxygenated (95% O2/5% CO2) artificial cerebrospinal fluid (aCSF in mM; 118 NaCl, 25 NaHCO3, 10 glucose, 2.5 KCl 2.5, 1.2 NaHPO4, 1.3 MgCl2, 2.5 CaCl2). Slices were kept at 33 °C for 30 min and then kept at room temperature for at least 30 minutes prior to recording. For drug application experiments, VM neurons were voltage clamped at −60mV. Tetrodotoxin (TTX 1 μM; Alomone) and 4-aminopyridine (4-AP 200 μM; Sigma) were used to eliminate action potential-dependent EPSCs, while augmenting light-induced, direct depolarization of ChR2-positive PPN/MNR terminals, resulting in the selective elimination of polysynaptic events. The non-NMDA iGluR antagonist 6-cyano-7-nitroquinoxaline-2,3-dione (CNQX 10 μM; Tocris) was used to block AMPA/ kainate iGluR receptors to confirm that the synaptic PPN/MNR to VM connection is glutamatergic.

Acute brain slices were transferred to the recording chamber of an upright microscope (Olympus BX50WI, Japan) fitted with a CCD camera (Michigan City, IN), LED system (Olympus pE-2 CoolLED, Japan) with YFP/RFP filter sets, and a Multiclamp 700B amplifier (Molecular Devices, USA). During the recording of VM neurons, slices were perfused with warmed oxygenated ACSF (32 ± 2 °C). Recording pipettes were pulled to a tip resistance of 4–6 MΩ when filled with an internal solution. The internal solution contained (in mM): 135 KMeSO4, 7 NaCl, 10 HEPES, 2 Mg2ATP, 0.3 Na3GTP, and 0.3% biocytin, pH7.3 adjusted with KOH, osmolarity 280–290 mOsm. In experiments where only voltage-clamp and no current-clamp recordings were made, the internal solution contained (in mM): 135 CsMeSO4, 8 NaCl, 10 HEPES, 7 Na-phosphocreatine, 2 Mg2-ATP, 0.3 Na3-GTP, 10 EGTA, 0.1 spermine, and 0.3% biocytin (pH, 7.3 with CsOH; Osmolarity, 280–290 mOsm). Electrode offset potentials were corrected prior to giga-ohm seal formation. ChR2-expressing axon terminals were light-activated with 5 or 100 ms whole-field LED illumination at blue excitation wavelengths (470 nm) at 1.8 mW (0.09 mW for subthreshold stimulation). Liquid junction potentials were not compensated for. The series resistance was typically between 10–30 MΩ and was monitored during the experiments. For voltage-clamp average responses, neurons were clamped at −60 mV, and at least 10 photostimuli (duration, 5 ms; wavelength, 470 nm) were given at 10 second intervals. Light-evoked response amplitudes were measured from baseline to peak. Response latencies were measured from the onset of the photostimulus, and jitter was defined as the standard deviation of the latencies. Data were acquired using AxoGraph X (AxoGraph, Australia), filtered at 10 kHz, and digitized at 20 kHz using an ITC-16 board (InstruTech, USA). Off-line analysis was performed with AxoGraph X.

### QUANTIFICATION AND STATISTICAL ANALYSIS

#### Behavioral analysis

To calculate the proportion of trials with licks, ‘lick early’ trials were excluded (Figures 2C, 7B, S2C, and S2D). To calculate the correct rate (i.e., the proportion of licks to the correct direction), ‘lick early’ trials and ‘no response’ trials were excluded (Figures 6C, S2C, and S6G). ‘Lick’ was defined as a contact of the tongue with the electrical lick ports. This explains the existence of trials with tongue movement (based on high-speed videography) but without ‘lick’ (Figure 6D, Stim w.o. lick).

For PPN/MRN stimulation experiments (Figure 6B) we plotted the proportion of trials with T_delay_ + 1.2 s < T_lick_ < T_delay_ + 1.8 s (T_lick_ denotes the timing of the first lick). Since stimulation was delivered at T_delay_ + 1.2s, this corresponds to a proportion of trials with licks within 0.6 s after the stimulation. An increase in lick during stim trials (Figures S6D, S6F, and S6H) is the difference in the proportion of trials with licks between stim trials and the Go cue omitted trials. To calculate the correct rate of stim trials (Figures 6C and S6G, right), we considered the first lick direction within T_delay_ + 1.2 s < T_lick_ < T_delay_ + 1.8s. The control (Figures 6C and S6G, left) is the correct rate of Go cue trials. For statistics, we performed hierarchical bootstrapping: first, we randomly selected animals with replacement, second, randomly selected sessions of each animal with replacement, and third randomly selected trials within each session with replacement.

#### Videography analysis

High-speed (400 Hz) videography of orofacial movement (side view) was acquired using a CMOS camera (acA2040–180km, Basler) with IR illumination (940nm LED, Roithner Laser). We used DeepLabCut (Mathis et al., 2018) to track the movement of the tongue, jaw, and nose (Figure 1B; Supplementary movies 1 and 2). For jaw and nose, movements along dorsoventral direction were analyzed. The amplitude of movement was normalized per session so that the mean position at T_delay_ + 1.2 seconds (time 0 in Figure 6D) was 0 and the maximum movement in the Go cue trials was 1. Note that the jaw moves downward after the Go cue, but due to this normalization, the value increases in Figure 6.

To calculate the onset of jaw and nose movements, we performed hierarchical bootstrapping. The mean trace was calculated based on these randomly selected trials. Next, we linearly detrended the mean trace based on its value between T_delay_ + 0.6 s and T_delay_ + 1.2 s (time 0 to −0.6 in Figure 6D). We identified the time point in which movement exceeds three times the standard deviation of the baseline before the Go cue (100 ms window). We repeated this procedure 1,000 times to estimate the mean and S.E.M.

To calculate the onset of tongue movement, we first calculated the cumulative distribution (c.d.f.) of the first time point when the tongue was detected by DeepLabCut after the Go cue (T_delay_ + 1.2 seconds). We subtracted the c.d.f. of a trial type of interest by the c.d.f. of the Go cue omitted trial (Figure 6D, dotted line). Movement onset is the time point at which the difference passes 0.05. We repeated this procedure with hierarchical bootstrapping 1,000 times to estimate the mean and S.E.M.

Following these methods, tongue detection onset in Fig6D: (Go cue) 64.3 (56.0–75.0) ms; mean (2.5–97.5% confidence interval); (stim followed by lick) 75.5 (50.0–118.0) ms; *p* = 0.194 (hierarchical bootstrap). Jaw movement onse in Fig6Dt: (Go cue) 33.2 (20.0–42.5) ms; (stim followed by lick) 69.7 (8.8–102.5) ms; *p* = 0.114 (hierarchical bootstrap). Nose movement onset in Fig6D: (Go cue) 43.0 (32.5–50.0) ms; (stim followed by lick) 61.9 (7.5–117.1) ms; *p* = 0.139 (hierarchical bootstrap). The null hypothesis for *p*-value is that the onset of Go cue trials is shorter than that in stim trials.

#### Extracellular recording analysis

JRClust (Jun et al., 2017a) (https://github.com/JaneliaSciComp/JRCLUST) with manual curation (all data except Figure S3A–S3D) or Kilosort2 (https://github.com/MouseLand/Kilosort2) were used for spike sorting. For Kilosort2, we used a combination of quality metrics (https://github.com/AllenInstitute/ecephys_spike_sorting) to extract potential good units for analysis: amplitude > 100 μV, ISI violation < 0.5, amplitude cutoff < 0.1, SNR > 2.5, spike width < 1.2 ms, and a presence ratio > 0.95 over the course of recording sessions.

During the tactile delayed-response task, we recorded 9472 neurons across 300 behavioral sessions from 53 mice. For ALM, in total, 6030 neurons were recorded across 173 behavioral sessions from 37 mice. For thalamus, in total, 640 neurons were recorded across 23 behavioral sessions from 4 mice. For midbrain, 2808 neurons were recorded across 102 sessions from 18 mice. In addition, we analyzed published data collected from mice trained in the same tactile delayed-response task: 611 thalamus neurons and 116 SNr neurons from (Guo et al., 2017), and 554 DCN neurons from (Gao et al., 2018). Spike widths were computed as the trough-to-peak interval of the mean spike waveform. In ALM, putative pyramidal neurons (units with spike width > 0.5 ms) were analyzed (Guo et al., 2014c). In thalamus and SNr, units with width > 0.35ms and < 0.35ms, respectively, were analyzed following the criteria in (Guo et al., 2017).

In the auditory delayed-response task (Figure S3A–S3D), we obtained 13139 units across 76 sessions from 15 animals. We analyzed 5072, 655, 607, 1145, and 1560 units in ALM, M1, thal_ALM_, SC, and PPN/MRN, respectively.

For Figures 1, 3, and 5, neurons with at least 40 correct lick-right trials and 40 correct lick-left trials were analyzed. Forty trials were randomly subsampled for each correct trial type (lick-right and left) to analyze latency and selectivity. To calculate the latency from the Go cue for each neuron, we assumed that spike generation follows a Poisson process. First, we calculated the baseline spike rate before T_go_ (time of the Go cue; 100 ms window to calculate the baseline). Second, we identified the first time point after T_go_ in which the spike rate becomes higher or lower than a significance level, α= 0.001 (*Poisson* distribution based on the baseline spike rate; we call this time point as T_*p* = 0.001_). We defined the latency as the last time-point in which the spike rate becomes higher or lower than a second significance level, α = 0.05 (*Poisson* distribution) between (T_go_, T_*p*=0.001_]. We took this two steps approach to avoid detecting small amplitude changes.

Delay- and response- selective cells (Figure S5C) are neurons with significant delay or response selectivity (*ranksum* test comparing spike counts in correct lick right vs. correct lick left trials during (T_go_ – 0.6s, T_go_), and (T_go_, T_go_ + 0.6s), respectively; Forty trials were randomly subsampled for each correct trial type; *p* < 0.05.)

For the peri-stimulus time histograms (PSTHs) in Figures 1C, 5B, and S1A, only correct trials were included. In Figures 2F, S2E, S6I, S8D, and S8E, correct, incorrect, and no-lick trials were pooled to compare control vs. perturbation conditions. PSTHs were smoothed with a 100 ms boxcar filter for plots including all epochs (e.g., Figure 1B) or with a 5 ms causal boxcar filter for plots zoomed in around the Go cue (e.g., Figure 5B). In Figures 3A, 5C, 7C, and S5A, spike rates were z-scored.

To plot the density of neurons in Figures 3C and 5A, we applied a 2D Gaussian filter (half-width = 250 μm) to the estimated location of each neuron. For each pixel we calculated the density as (number of neurons with short latency or delay selectivity) / (number of neurons + 0.05). The 0.05 was added to the denominator to prevent pixels with low numbers of neurons from having an extremely high estimated density.

#### Coding direction analysis

To calculate delay coding direction (**CD**_**delay**_) for a population of *n* recorded neurons, we looked for an n × 1 unit vector that maximally distinguished the two trial types in the *n-dimensional* activity space. For each time point *t*, we defined a population selectivity vector: ***w***_*t*_ = ***r***_lick-right, *t*_ − ***r***_lick-left, *t*_, where ***r***_lick-right, *t*_ and ***r***_lick-left, *t*_ are n × 1 vectors of spike rate of individual neurons averaged across correct lick right and left trials without optogenetic manipulations (unperturbed correct trials) respectively. **CD**_**delay**_ is ***w***_***t***_ averaged over the last 600 ms of the delay epoch (T_go_ − 0.6 s < t < T_go_) and normalized it by its norm. The coding direction after the Go cue (**CD**_**response**_) was calculated similarly over the first 400 ms of the response epoch (T_go_ < t < T_go_ + 0.4 s). We then orthogonalized **CD**_**response**_ to **CD**_**delay**_ using the Gram-Schmidt process. To calculate the go direction (**D**_**go**_), we subtracted (***r***_lick-right, *t*_ + ***r***_lick-left, *t*_)/2 after the Go cue (T_go_ < t < T_go_ + 0.1 s) from that before the Go cue (T_go_ − 0.1 s < t < T_go_), followed by normalization. The ramping direction (**D**_**ramp**_; Figure S1I) was defined as a vector maximally distinguishing the mean activity before the trial onset (0.6 s window) and the mean activity before the Go cue (0.1 s window). All directions were orthogonalized to each other using the Gram-Schmidt process in Figures 1F and S1I. The stimulation direction (**D**_**stim**_; Figure S6E and S6F) was defined as a vector maximally distinguishing control (Go cue omitted) and stimulation trials after the stimulation (T_go_ < t < T_go_ + 0.1 s).

In each recording session we randomly selected 50 % of unperturbed correct trials to calculate directions in individual recording sessions (Figures 2, 6, 7, S1H, S1J–S1L, S2, S6, S7 and S8). We then projected the spike rate in the remaining trials to the calculated directions to obtain trajectories. To pool trajectories across sessions, we normalized projections in each session based on the mean trajectories of the unperturbed correct trials. The projection to **CD**_**delay**_ was normalized by the mean activity before the Go cue (T_go_ − 0.1 s < t < T_go_) so that activity in the lick left trials becomes 0 and in lick-right trials 1. The projection to **CD**_**response**_ in each session was normalized by the mean activity after the Go cue (T_go_ < t < T_go_ + 0.4 s) so that activity in lick left trials becomes 0 and in lick right trials 1. **D**_**go**_ in each session was normalized by the mean activity around the Go cue (mean activity difference before and after the Go cue, 100 ms window, to be 1). The plots (Fig. 2, 6, 7, S1,S2, S6, S7 and S8) show grand medians. Hierarchical bootstrapping was used to estimate S.E.M. Neurons with low spike rates (less than 2 spikes per s) were excluded from analysis. Sessions with less than five cells or without significant selectivity before the Go cue (100 ms window, p > 0.05, *ranksum* test) were excluded from session-based analysis because directions could not be well defined (34 /129 sessions). In Figure 6E and S6L–S6P, sessions with stimulation-triggered licks were analyzed (21 sessions).

In Figure 5 we performed latency analysis across sessions and we thus calculated directions by subsampling cells recorded across sessions (i.e., projection is not based on simultaneous recordings) (Figure 1 and S1I were performed similarly for consistency). For each cell, we subsampled 20 unperturbed correct lick right and left trials each to define directions. Then, we projected the spike rate in the other 20 trials to these directions as an inner product to obtain trajectories. We excluded cells with less than 40 correct trials per lick direction. To calculate the “selectivity explained” (Figure 1F), we first calculated the total selectivity as the squared sum of selectivity across neurons (squared sum of n × 1 vector). We then calculated the squared sum of selectivity of the projection along each mode at each time point. To calculate the “activity explained” (Figure S1I), we calculated the squared sum of the spike rate after subtracting the baseline (mean spike rate before the sample onset; 0.6 s window) across neurons. Similarly, we calculated the squared sum of the activity along each direction after subtracting the baseline. In Figures 1G, 5E, and 5F, the mean of correct lick right and left trials is shown for **D**_**go**_, and the difference between correct lick right and left trials is shown for **CD**_**delay**_ and **CD**_**response**_. Projections are boxcar filtered (causal, 10 ms window). In Figures 5E and 5F, projections are standardized by the activity before the Go cue (T_go_ − 0.1 s < t < T_go_). To calculate the latency to the Go cue (Figures 1G, 5E and 5F), we first identified the first time point in which the projection passes five times of the standard deviation after the Go cue (T_std = 5_). We then defined the latency as the last time point in which the projection passes two times of the standard deviation between (T_go_, T_std_ =5]. We subsampled 1,000 cells with replacement in Figures 1E–1G and S1I. In Figure 5, to match the data size across brain areas, we randomly subsampled 226 cells with replacement to calculate the direction and projection (except for SNr and NLL, where we had 116 and 23 cells, respectively; in these areas, we randomly sampled 116 and 23 cells with replacement). We repeated this subsampling 1,000 times.

#### Histology analysis

Each coronal section was made up of 80–200 image tiles merged with Neurolucida software. The whole-brain image stack was registered to the Allen Institute Common Coordinate Framework (CCF) (Wang et al., 2020) of the mouse brain using NeuroInfo software (MBF Bioscience, Williston, VT) or using a Matlab-based script (Mike Economo, Boston University). For cell counting (Figure S4), neurons labeled with AAV_retro_ were detected with a Laplacian of Gaussian algorithm using NeuroInfo software (MBF Bioscience, Williston, VT). Tips of the probe tracks were annotated manually and transformed into the Allen CCF to estimate recording sites (Figure S3F). In Figures 4A and S4D, pixel intensities are normalized: (signal in each pixel – mean signal in cortical areas)/ (99.5 percentile signal in the image – mean signal in cortical areas); since these subcortical areas do not project to the cortex, the cortical signal was used as a baseline for normalization. In Figure 4B, the mean normalized pixel intensities were averaged per nucleus in thal_ALM_. To define thal_ALM_, we injected WGA-Alexa555 in ALM (Guo et al., 2017). After registering to the Allen CCF, 10 μm voxels with retrogradely labeled cells (n = 3 mice) were defined as thal_ALM_.

For fluorescent *in situ* hybridization (Figures S4G and S4H) we used hybridization chain reaction (HCR; Molecular Instruments) (Choi et al., 2018) on 150 μm thick coronal sections, following the protocol described in (Nicovich et al., 2019). We probed for *Chat* (H1), *Gad1* (H2) and, *Slc17a6* (*VGglut2*; H3) with amplifiers conjugated with Alexa546 (for H1), Alexa647 (for H2), and Alexa594 (for H3) (See Supplementary Table 4 for probe sequences). Images were acquired with an LSM880 confocal microscope (Zeiss) using a Plan-Apochromat 40x N.A. 1.3 Oil objective.

#### Statistics

The sample sizes are similar to the sample sizes used in the field. No statistical methods were used to determine sample size. During experiments, trial types were randomly determined by a computer program. During spike sorting, experimenters cannot tell the trial type and therefore were blind to conditions. All comparisons using *signed rank* and *ranksum* tests were two-sided. All bootstrapping was done over 1,000 iterations.

## Supplementary Material

1 Figure S1. Related to Figure 1. Activity underlying motor planning and movement initiation in ALM
A. Example neurons in ALM. Top, spike raster. Bottom, mean spike rate. Blue, correct lick right trials; red, correct lick left trials. Time is aligned to the onset of the Go cue. Dashed lines separate behavioral epochs. S, sample epoch; D, delay epoch; R, response epoch.
B. Pearson’s correlation of the population activity vector is low between time points before and after the Go cue. Dashed lines separate behavioral epochs.
C. Go cue activity (mean spike rate after the Go cue – mean spike rate before the Go cue; 100 ms window) is similar between lick right (x-axis) and left (y-axis) trials. This is consistent with non-selective **D**_**go**_. Circles, individual neurons in ALM (5136 neurons).
D. Selectivity during the delay and response epochs is not consistent. Left, the relationship between delay selectivity (mean selectivity during the last 600 ms of the delay epoch) and response selectivity (mean selectivity during the first 400 ms of the response epoch). Circles, individual neurons in ALM (5136 neurons). Inset, the definition of θ (angle in a polar coordinate). Right, histogram of θ across neurons. θ ~ Ꮐ/4 indicates similar selectivity during the delay and response epoch, whereas θ ~ 0 or π/2 indicates selectivity is strong only in the delay or response epoch, respectively.
E. Increase in conductance of ALM neurons after the Go cue. Membrane potential (Vm) of ALM neurons during the tactile task without (top) or with (bottom) negative current injection in correct lick right trials. Change in Vm (ΔVm) from that before the Go cue (100 ms window) is shown. Thin lines, each neuron. Thick line, mean. Results in correct lick left trials were similar (data not shown). We manipulated the membrane potential during whole-cell recordings in ALM to observe changes in synaptic conductances around the movement initiation. To enhance either excitatory or inhibitory synaptic potentials, we injected negative or positive currents into the neurons to hyperpolarize or depolarize them. Using this method, we confirmed rapid onset increases in both excitatory (**E**) and inhibitory (**F**) conductances after the Go cue (arrowheads). This reflects dramatic reorganization of synaptic input after the Go cue, which presumably underlies the mode switch.
F. Same as **E** for positive current injection. The decrease in Vm with positive current injections indicates an increase in inhibitory current after the Go cue (arrowhead).
G. Projections of activity along **CD**_**response**_ without orthogonalization. The same format as in Figure 1E.
H. Histograms of angles between different modes across recording sessions (n = 129 sessions). Left, angles between $CD_{\mathrm{delay}}=\bar{w_{t}}$ (−0.6 s < t < 0 s; time from Go cue) and $CD_{\mathrm{response}}=\bar{w_{t}}$ (0 s < t < 0.4 s; time from Go cue) are significantly larger than 0 and closer to π/2 without explicit orthogonalization, consistent with the inconsistent selectivity before and after the Go cue shown in **D**. Middle and right, **D**_**go**_ is near orthogonal to **CD**_**delay**_ and **CD**_**response**_.
I. The activity in each trial type can be mostly explained by projections to four modes (Methods).
J. Decoding of trial type. ROC analysis to distinguish correct lick right vs. left trials using activity along each mode. Thin lines, individual sessions (n = 129 sessions; 50 ms bin). Thick line, mean. AUC, area under the curve of the ROC curve.
K. Activity aligned to movement onset indicates that mode switch happens prior to movement, and is thus not due to efference copy or sensory feedback. Projection of activity along **CD**_**delay**_, **CD**_**response,**_ and **D**_**go**_ aligned to the first time point the tongue was detected based on high-speed videography (e.g., time 100 ms in Figure 1B).
L. Trial-by-trial correlation of activity along different modes (mean of 129 sessions; showing correct lick right trials; lick left trials are similar, data not shown). Activity along **CD**_**response**_ after the Go cue (t>0, x-axis) shows high correlation with activity along **CD**_**delay**_ before the Go cue (t < 0, y-axis) (left), but not in trial-shuffled controls (middle) or with activity along **D**_**go**_ (right). High correlation implies that trials with higher activity along **CD**_**delay**_ before the Go cue tend to show high activity along **CD**_**response**_ after the Go cue.

2 Figure S2. Related to Figure 2. Attenuated CD_response_ but intact D_go_ with silencing of ALM output
A. Coronal brain sections showing bilateral expression of stGtACR1 in PT_lower_ cells. Left, ALM. Right, injection sites in the medulla (see Supplementary Table 1 for coordinates). Red, the fluorescence of FusionRed fused to stGtACR1. Blue, DAPI.
B. Schema of PT_lower_ silencing experiments.
C. Calibration of laser power. Behavioral effect of PT_lower_ silencing with different laser intensities. Thin lines, individual animals (n = 4 mice). Thick line, mean. PT_lower_ silencing in ALM decreased the proportion of trials with lick without affecting correct rate (probability to lick the correct direction). Because of the significant behavioral effect in ALM with modest effect in M1, we selected 0.5 mW for the rest of the experiments. *: *p* < 0.05; **; *p* < 0.01 (*Bootstrap* with *Bonferroni* correction for multiple comparisons; null-hypothesis is that the proportion of lick or correct rate in control trials is lower than or equal to those in silencing trials).
D. PT_lower_ silencing resulted in loss of lick within 0.6 s after the Go cue (Figure 2C), whereas the probability to lick within 1.5 s after the Go cue (laser is on for 1s after the Go cue) was less affected (*p* = 0.003, *bootstrap*). This indicates that licking recovers after the laser stimulation (e.g., Figure 2B). Note that mice lick the correct direction (Figure S2C, correct rate). Same n = 3 mice as in Figure 2C.
E. Example putative PT_lower_ cells (cells with a significant decrease in activity during the silencing; left three cells) and PT_lower_-inhibited cells (cells with a significant increase in activity during the silencing; right three cells). Top, raster; middle and bottom, mean spike rates of trials with and without PT_lower_ silencing. Blue, mean of all lick right trials (including correct, incorrect, and no lick trials); red, mean of all lick left trials; cyan bar, laser on.
F. Grand average PSTH of putative PT_lower_ cells (n = 150 cells), and PT_lower_-inhibited cells (n = 129 cells). Note that putative PT_lower_ cells do not have a contralateral bias on average. Line, grand mean of neurons; shading, S.E.M. (*hierarchical bootstrap*); blue, mean of all lick right trials; red, mean of all lick left trials; cyan bar, laser on.
G. Projection of activity along **CD**_**response**_ defined only using putative PT_lower_ cells (left) and all putative pyramidal cells excluding putative PT_lower_ cells (right, non-PT_lower_ cells). Consistent with strong silencing of putative PT_lower_ cells regardless of trial types (Figure S2F), activity along **CD**_**response**_ collapsed in both trial types (left). In contrast, **CD**_**response**_ defined by non-PT_lower_ cells showed a reduction in activity only in lick right trials, indicating that contralateral reduction in **CD**_**response**_ is a network effect. Neurons across sessions were pooled for this analysis (n = 150, 749 cells, respectively). Line, grand mean; shading, S.E.M.; cyan bar, laser on.
H. Lack of stGtACR-mediated axonal excitation of ALM neurons in the PT_lower_ silencing experiments in contrast to PPN/MRN neurons in the PPN/MRN silencing experiment (related to Figure 7). In PPN/MRN, we observed increase in spike rates ~10 ms after the stimulation onset (right). We did not see such short latency increase in spike rate in PT_lower_-inhibited cells (left), although these cells increase spike rate during PT_lower_ silencing on average (**F**). Top, activity at the light onset. Difference in mean PSTH between light-on and control trials are shown. Blue, lick right trial; red, lick left trial; line, mean; shading, S.E.M. Bottom, spike rate of individual neurons before and after the light onset. Filled circles, *p* < 0.05 (signed rank test, significant difference between stimulation vs. control; n = 129 and 158 cells for PT_lower_ and PPN/MRN silencing, respectively). The soma of PT_lower_ cells reside in deep layer 5 (~800 μm) and lack axonal arborization in the cortex (Economo et al., 2018). Considering limited penetration of blue light (473 nm; light intensity attenuates to be less than 20% at 500 μm below the surface) (Li et al., 2019), we are likely silencing the dendritic arbor of PT_lower_ cells, which helps explain the lack of stGtACR1-mediated “axonal” excitation in this experiment.
I. Projection of activity along **CD**_**delay**_, **CD**_**response,**_ and **D**_**go**_ in “no response” trials. Activity along **CD**_**delay**_ during the delay epoch is attenuated in no response trials. Activity along **CD**_**response**_ in lick right trials is attenuated in no response trials. Line, grand median of sessions (n = 24 sessions); shading, S.E.M. (*hierarchical bootstrap*).
J. Quantification of Figures 2G and S2I. CD_delay_ during the delay epoch and the change in activity after the Go cue along each direction (activity after the Go cue – activity before the Go cue; 200 ms window) are shown. Cumulative distribution function (c.d.f.) across hierarchical bootstrap trials (1000 iterations). *P-value*, hierarchical bootstrap with a null-hypothesis that activity changes in control trials are smaller than or equal to those in silencing (or no response) trials.

3 Figure S3. Related to Figure 3. Thalamic and mibrain activity.
Latency to Go cue across brain areas (**B-D**) are similar (latency in PPN/MRN < latency in thal_ALM_ < latency in ALM) in a different task: auditory delayed-response task (**A**). Data in **E** is based on the tactile task (related to Figures 3D–3F).
A. Auditory delayed-response task. Tones (3 or 12 kHz) instead of tactile cues were presented during the sample epoch to instruct lick direction. Go cue is 6 kHz FM sound (Methods).
B. Recording in the thalamus (top) and midbrain (bottom). Each region filled with color indicates different thalamic or midbrain nuclei. White contour, thal_ALM_. Black dots, location of individual recorded neurons in the Allen common coordinate framework (CCF). Green, neurons with < 20 ms (top; in the thalamus) or <15 ms (bottom; in the midbrain) latency to the Go cue.
C. Cumulative distribution (c.d.f.) of latency to the Go cue in ALM and M1. Latency (mean ± S.E.M.; time point in which 1% of recorded cells increase activity): 21.1 ± 0.5 ms (ALM; n = 5072 units) and 20.3 ± 4.9 ms (M1; n = 674 units). *P* = 0.402 (bootstrap with a null hypothesis that the latency in M1 is equal to or faster than ALM).
D. c.d.f. of latency to the Go cue across brain areas. Latency (mean ± S.E.M.; time point in which 1% of recorded cells increase activity): 21.1 ± 0.5 ms (ALM; n = 5072 units); 16.0 ± 1.5 ms (thal_ALM_; n = 607 units); 10.1 ± 0.8 ms (SC; n = 1145 units); and 7.2 ± 0.5 ms (PPN/MRN; n = 1560 units).
E. Distribution of thalamic neurons with decreased delay activity during ALM silencing (left, schema). Note that neurons within thal_ALM_ (white contour) were strongly silenced, consistent with the strong excitatory drive from ALM to thal_ALM_ (Guo et al., 2017). Top, location of individual recorded neurons in the Allen CCF (black). Neurons with more than 50 and 75% reduction in spike rates during ALM silencing (green and red, respectively). Bottom, the density of neurons with more than 50% reduction in spike rates.
F. Identification of recording sites. Probes were painted with CM-DiI, which leave tracks with fluorescent signal. After slicing the brain, each 50 *μ*m thick section was imaged. After annotating each track, the images were registered to Allen CCF (Methods). Left, a raw image of an example section; green circles, tips of the probe. Right, tracks in Allen CCF; red line, estimated probe location in Allen CCF; green dot, estimated tip location; blue dots, other markers placed along the track to estimate the probe location.

4 Figure S4. Related to Figure 4. Neurons projecting to thal_ALM_.
Characterization of thal_ALM_-projecting neurons based on retrograde (**A-C**) and anterograde labeling (**D-F**). In addition, we confirmed that most thalamus-projecting PPN/MRN neurons are glutamatergic using the fluorescent in situ hybridization (FISH; **G**, **H**), immunostaining (**I**, **J**), and acute slice recording (**K-Q**).
A. Quantification of retrogradely labeled cells in an animal with retrobeads injection in thal_ALM_. The total pixel intensities of retrobeads signal in midbrain/hindbrain areas are shown. Blue, contralateral hemisphere; red, ipsilateral hemisphere to the injection site. Original images of this sample are reported in (Guo et al., 2017).
B. Quantification of retrogradely labeled cells in an animal with AAV_retro_ injection in thal_ALM_. The number of labeled cells in midbrain/hindbrain areas are shown. Blue, contralateral hemisphere; red, ipsilateral hemisphere to the injection site. Error bar, standard deviation (n = 2 mice). Some inconsistencies between the retrobeads and AAV_retro_ are caused by known viral tropism (e.g., weak labeling of SNr by AAV_retro_ (Tervo et al., 2016)) and a spread of AAV_retro_ at the injection site beyond thal_ALM_ (Figure S4C).
C. Distribution of retrogradely labeled cells in an animal with AAV_retro_ injection in thal_ALM_. Images are registered to Allen common coordinate framework (CCF). AP, relative to Bregma. Heatmap indicates the number of labeled cells per voxel (size: 10 × 10 × 1000 *μ*m).
D. Anterograde labeling from distinct subcortical areas to thal_ALM_. Images are registered to Allen CCF. AP, relative to Bregma. Unlike PPN/MRN projection (Figure 4A), projections of these structures are more localized.
E. Quantification of anterograde labeling from PPN/MRN to different thalamic nuclei within thal_ALM_. Projection is stronger to the ipsilateral hemisphere.
F. Similarity of axonal projection pattern from each subcortical area (i.e., pixel intensities in Figures 4A and S4D), and the distribution of thal_ALM_ neurons with fast go cue responses (i.e., Figure 3C, second row). When ***P*** = pixel intensity and ***D*** = distribution of fast go cue response, we normalized ***P*** and ***D*** by their own norms and calculated the inner dot product between them. A larger number indicates a higher similarity.
G. Example images of FISH. Left, PPN/MRN_Th_ neurons labeled by AAV_retro_-CamKII-GFP injected in thal_ALM_. Right, FISH of the same section with probes against *vglut2*, *chat*, and *gad1*.
H. Quantification of neurotransmitter type (i.e., *vglut2*, *chat*, and *gad1*) of PPN/MRN_Th_ cells labeled by AAV_retro_-CamKII-GFP (n = 880 cells) or AAV_retro_-CAG-GFP (n = 404 cells) injected in thal_ALM_. Cells not labeled by any neurotransmitter probes were excluded from the analysis. Cells labeled by CamKII promoter were predominantly *vglut2* positive (Roseberry et al., 2016).
I. Anti-ChAT immunostaining (green) of a coronal section with PPN/MRN_Th_ neurons labeled by AAV_retro_-CAG-H2B::TdTomato injected in thal_ALM_ (magenta). Consistent with FISH (Figures S4G **and** S4H), PPN/MRN_Th_ were mostly ChAT-negative. Blue, Nissl staining.
J. Enlarged image of **I**.
K. Schema of the acute slice recording experiments. We expressed ChR2 in Glutamatergic PPN/MRN neurons using AAV-DIO-ChR2-mCherry in Vglut2-IRES-Cre mice.
L. Mean EPSC (bar) and individual responses (dots). Error bar, S.E.M. Inset, example VM neuron voltage-clamped at −60 mV. Gray lines, twenty individual responses to a single 5 ms 470 nm light pulse (blue bar). Black line, mean. 48/74 VM neurons received PPN input. Latency of EPSC: 2.8 ± 0.7 ms (mean ± standard deviation, n =37 cells).
M. Mean EPSC amplitude in ACSF (black bar) and in the presence of TTX (1 mM) and 4-AP (200 mM, gray bar). Currents were not abolished in the presence of TTX and 4-AP demonstrating monosynaptic input from PPN/MRN to VM. Individual experiments in ACSF (black dots), TTX and 4-AP (gray dots). Neurons were voltage-clamped at −60 mV. Error bars represent S.E.M, n = 10.
N. Mean EPSC amplitude before (black bar) and after the application of 10 mM AMPA/ kainate-selective antagonist CNQX (purple bar). Application of CNQX blocked the EPSC, as expected for glutamatergic input. Individual experiments in ACSF (black dots, n = 7) or with TTX and 4-AP (gray dots, n = 4) before and after CNQX application. Neurons were voltage-clamped at −60 mV. Error bars represent S.E.M, n = 11, *p* < 0.05 (two-tailed paired *t*-test).
O. Example of evoked action potential (black trace) in response to a 40 Hz 1.8 mW light pulse train (cyan bars) at the resting membrane potential, overlaid with potential in response to a subthreshold light pulse train (0.09 mW; gray line).
P. Example VM neuron responding to PPN input stimulation with 20, 50, 100, 150, 250, and 400 ms light pulse intervals.
Q. Average paired-pulse ratio of five VM neurons. Neurons were voltage-clamped at −60 mV. Error bars represent S.E.M.

5 Figure S5. Related to Figure 5. Latency to the Go cue across brain areas.
A. Spike rates of neurons sorted by their latency to the Go cue in each brain area. From left to right: neurons with an increase in spike rate (go-up cells) in lick right trials and lick left trials and neurons with a decrease in spike rate (go-down cells) in lick right trials and lick left trials. The top 20% of cells and 10% of cells are shown for go-up and go-down cells, respectively. Spike rates were normalized by the spike rate before the Go cue (100 ms) and shown as a heatmap. See Methods for the number of cells recorded in each area.
B. Grand average PSTH of the go-up and go-down cells. Line, grand average; shading, S.E.M. (*bootstrap*); blue, lick right trial; red, lick left trial.
C. Proportion of neurons with selectivity during the delay (top) or response (bottom) epoch in each area. Blue, lick right trial; red, lick left trial; error bar, S.E.M. (*bootstrap*); dashed line, chance level (*p* = 0.05 as selectivity was defined by ranksum test with α = 0.05).
D. Same as Figure 5D, but a broader time-window is shown. Each color indicates a different brain area (box below **F**). Fraction of Go-up cells (<15 ms latency) is 4.5 % and Go-down cells is 0.15 % in PPN/MRN.
E. Overlay of grand average PSTH of the go-up and go-down cells. The mean spike rate before the Go cue (100 ms window) was subtracted.
F. Proportion of neurons with significant (ranksum test, *p* < 0.01) increase or decrease in activity after the Go cue (compared to before the Go cue; 100 ms window) at each time point (10 ms bin).
G. Grand average PSTH of neurons in PPN/MRN (top) and thal_ALM_ (bottom) in trials with the Go cue (black) or a different tone (green). Related to Figure 5G. In both areas, neurons specifically responded to the Go cue. Both lick right and left trials were pooled.
H. Spectrogram of sound recorded during the task. Note the signals containing a broad frequency spectrum at the start and end of the sample epoch. This hiss was created by a pneumatic valve that moves the tactile stimulus. The Go cue is a pure tone (3kHz). S, sample epoch; D, delay epoch; R, response epoch.
I. Mean PSTH of Go-up cells across brain areas. All brain areas show transient increases in spike rate at the beginning and end of the sample epoch.
J. Ratio of the Go cue response (peak activity after the Go cue; 200 ms window) vs. response to the hiss (peak activity at the end of the sample epoch; 200 ms window). Higher values mean neurons “tuned” to the Go cue. Note that majority of neurons in PPN/MRN and downstream areas have values higher than 0 (i.e., stronger response to the Go cue).

6 Figure S6. Related to Figure 6. Stimulation of thalamus-projecting PPN/MRN triggers licking responses.
A. Anatomical location of fiber optics in the thalamus. Each region filled with a color indicates a different thalamic nucleus. Red, MD; yellow, IL; green, VAL; blue, VM. After recordings, brains were imaged and registered to Allen CCF (n = 20 mice). Black cross, tips of fiber optics in mice without stimulation-triggered lick; red cross, the same in mice with stimulation-triggered licks. Top, sagittal view; bottom, coronal view (AP −1.38 mm from Bregma).
B. Anatomical location of the center of virus injection in PPN/MRN. Each region filled with a color indicates a different midbrain nucleus. Red, MRN; blue, PPN; green, cuneiform nucleus. Same animals as analyzed in **A**. Top, sagittal view; bottom, coronal view (AP −3.92 mm from Bregma).
C. YFP (conjugated to ChR2) signal around the injection site (mean of 3 mice). Signal intensity is shown in the colormap. The injection site has the strongest signal (red). Weaker signals (cyan) are projections. Top, Allen CCF; bottom, coronal view (AP −4.1 mm from Bregma).
D. Anatomical location of viral injection along anterior-posterior (AP) axis and increase in lick in stim trials (probability to lick in stimulation trials – probability to lick in Go cue omitted trials). There is a trend that posterior injection results in a higher probability of stimulation-triggered licks. We see a similar trend with the GtACR experiment as well (Figures S8A–S8B; HI211 and 215 reduced licks with the weaker 0.25mW laser power). CI, confidence interval based on bootstrap.
E. Explanation of cross-animal variability in the probability of stimulation-triggered licks based on ALM activity. We defined a stimulation direction (**D**_**stim**_), which distinguishes activity with or without stimulation in ALM (Methods). Activities in Go cue trials projected along **D**_**stim**_ are different between mice with (top) or without (bottom) stimulation-triggered licks. In mice with stimulation-triggered licks, activity along **D**_**stim**_ increased mostly after the Go cue (green boxes). In mice without stimulation-triggered licks, the activity along **D**_**stim**_ also increased in response to sensory cues during the sample epoch (tactile cue and sound caused by the pole movement; magenta box). Thus, in the latter animals, the stimulation did not induce specific activity patterns similar to that induced by the Go cue, explaining the lack of lick. This may be due to the differences in the location of viral injection and/or fiber optics (Figures S6A–S6D). Line, median. Shading, S.E.M.
F. The difference in activity along **D**_**stim**_ between the post-Go cue (green in Figure S2E) and sample epoch (magenta in Figure S2E) vs. increase in lick rate in PPN/MRN_Th_ stimulation trials. *P-value*, bootstrap with a null hypothesis that correlation is smaller than or equal to 0. Circle, each animal (n = 20 mice).
G. Mice with unilateral virus injection licked correct direction in response to the PPN/MRN_Th_ stimulation. Blue and red, lick right and left trials, respectively (n = 4 mice; one mouse did not lick in response to the stimulation, and the correct rate is not defined).
H. The proportion of trials with stimulation-triggered licks decreased over sessions, presumably because we did not reward stimulation-triggered licks. Lines, individual mice.
I. Activity of example neurons in ALM. Top, spike raster. Bottom, mean spike rate. Time is aligned to the Go cue (or timing of the normal Go cue/stimulation). Mean spike rate is shown for Go cue trials and stim followed by lick trials.
J. Grand average PSTH of neurons with an increase (left; n = 50 cells) or decrease (right; n = 42 cells) in spike rate after the Go cue (by more than two spikes per s), comparing go-cue trials (top) and stim trials with licks (bottom).
K. Pearson’s correlation of population activity vector, (***r***_lick-right *t*_ + ***r***_lick-left *t*_)/2, across trial types. Population activity patterns were similar between trials with stimulation-triggered licks and cue-triggered licks (4_th_ panel). n = 211 cells across sessions were pooled. The same number of trials were subsampled across trial types.
L. Quantification of activity after the Go cue (50 ms, top; 100 ms, bottom) along each direction. Cumulative distribution across bootstrap trials shown in Figure 6E (1000 iterations). *P-value*, from left to right, comparison between Go cue omitted vs. Stim no lick, Go cue omitted vs. Stim followed by lick. The null hypothesis is that the change in activity after the Go cue in Go cue omitted trials is bigger than or equal to that in Stim trials.
M. Activity aligned to movement onset indicates that mode switch happens prior to movement in the stimulation trials. Projection of activity along **CD**_**delay**_, **CD**_**response,**_ and **D**_**go**_ aligned to the first time point the tongue was detected based on high-speed videography. The Go cue trials data (top) is a duplicate of Figure S1K, shown here for comparison. The same n = 21 sessions (12 mice) as analyzed in Figure 6E.
N. Decoding of stimulation-triggered lick based on activity along **D**_**go**_. We performed ROC analysis to test the trial-by-trial relationship between the increase in activity along **D**_**go**_ after the PPN/MRN_Th_ stimulation (100 ms window) and whether the animal licked or not in response to the PPN/MRN_Th_ stimulation. We analyzed sessions with more than five trials of stimulation-triggered licks (n = 7 sessions). X-axis, false-positive rate; y-axis, true-positive rate. *P-value*, bootstrap with a null hypothesis that AUC <= 0.5.
O. Relationship between the increase in activity after the Go cue along **D**_**go**_ and **CD**_**response**_ (50 ms, top; 100 ms, bottom). Dot, individual bootstrap trial (1000 iterations). Activity change along **CD**_**response**_ is correlated with that along **D**_**go**_.
P. Relationship between activity along **CD**_**delay**_ and activity along **CD**_**response**_ (mean activity after the Go cue, time of normal Go cue, or stimulation; 100 ms window). Dot, individual trial (pooled from all sessions with stim-triggered licks; n = 21 sessions); line, decision boundary of Fisher’s linear discriminant analysis (LDA) distinguishing lick right vs. left trials. The vertical line (e.g., in Go cue omitted) indicates no relationship between **CD**_**delay**_ and **CD**_**response**_, whereas negative slopes (e.g., in Go cue trials and stim followed by licks) indicate a strong relationship between **CD**_**delay**_ and **CD**_**response**_.

7 Figure S7. Related to Figure 7. Muscimol-mediated silencing of PPN/MRN
A. Infusion location. One hemisphere is shown, although infusions were bilateral. Crosses, locations of the cannula confirmed by post-hoc histology (n = 8 mice; colors correspond to the animals shown in B).
B. Behavioral effect of bilateral muscimol infusion. All animals with PPN/MRN infusion showed significant reduction in response rate with <= 5 ng muscimol. Muscimol was dissolved in cortex buffer. Control, infusion of cortex buffer without muscimol. **: *p* = 0.00012 (two-tailed paired *t*-test comparing 0 vs 5 ng muscimol, n = 6 animals).
C. Activity in ALM along **D**_**go**_ before and after infusion. The Go cue response was significantly attenuated after bilateral muscimol infusion. The first 20 trials after infusion were analyzed (same in **D** and **E**). Line, mean; shading, S.E.M. n = 73, 250 cells in 6 PPN/MRN infusion mice (control, muscimol, respectively; same in **D, E, G** and **H**).
D. The amplitude of the Go cue response before and after the infusion for single neurons. Circle, individual neuron. Filled circle, cell with significant go cue response before infusion (*p* < 0.05, signrank test).
E. Delay selectivity before and after the infusion. In addition to the loss of Go cue response, delay activity (non-selective ramping activity and selectivity during the delay epoch) in ALM became weaker after muscimol infusion (**E** and **H**). The attenuated delay activity is likely due to lack of water reward during and after the infusion protocol. After an infusion we waited for 5 minutes for muscimol or control cortex buffer to diffuse, during which animals did not receive water reward. Low expected reward and motivational state attenuate delay activity (Roesch and Olson, 2003; Allen et al., 2019). Consistent with this idea, even under control conditions (i.e. no muscimol), delay activity dropped after infusion, and gradually recovered after water consumption during the task (**H**). After bilateral muscimol infusion delay activity did not recover, which can be explained by lack of water consumption. Importantly, despite the transient decrease in delay activity, the Go cue response remained constant after the control infusion, implying the effect of infusions on delay activity and Go cue response are independent (**G** and **H**, first column). Circle, individual neuron. Filled circle, cell with significant delay selectivity before infusion (*p* < 0.05, ranksum test).
F. Time-course of the response rate (proportion of trials mice licked after the Go cue). Causal box car filtering (bin = 3 trials; same in **G** and **H**).
G. Time-course of the absolute Go cue response in ALM.
H. Time-course of the absolute delay activity in ALM.
I. To show that strong excitation prior to the Go cue does not preclude another mode switch and movement initiation after the Go cue, we played an additional “Go cue sound” in the middle of the delay epoch (to mimic the effect of stGtACR-mediated excitation introduced during perturbation). This ‘fake Go cue’ does not result in reward. Schema of two trial types (left) and lick timing in an example session (right). The fake Go cue triggered strong excitation of ALM and the mode switch (increase in **CD**_**response**_, and **D**_**go**_). Yet, it did not interfere with lick initiation and mode switch caused by the Go cue at the end of the delay epoch (**I**-**K**). Thus, strong population activity triggered by stGtACR at light onset by itself does not explain the loss of Go cue response.
J. ALM activity during the fake go cue experiments. Top, control trials. Bottom, fake cue trials. Blue, correct lick right trials (dashed line, activity in control trials). Red, correct lick left trials. Activity along **D**_**go**_ is similar even when we pooled all trials including incorrect trials and no response trials (not shown). n = 180 cells.
K. Comparison of the Go cue response in control trials and fake cue trials. All trials (including incorrect and no response trials) were included to calculate mean Go cue response. n = 180 cells, 3 mice.

8 Figure S8. Related to Figure 7. Perturbation of thalamus-projecting PPN/MRN neurons blocks movement initiation
A. Anatomical location of the tips of fiber optics (cross) in PPN/MRN (n = 4 mice; HI## are animal names). Sagittal view. Each region filled with a color indicates a different midbrain nucleus. All data in Figure S8 except **G** is based on animals injected with AAV_retro_-CamKII-Cre in the thalamus.
B. Coronal view. Same brains as analyzed in **A**. AP −4.34 mm from Bregma.
C. Raster plot of lick timing in all animals. 0.25 and 1 mW indicate laser power used for perturbation. Cyan box, laser on. Behavioral effects were stronger in HI211 and 215.
D. Example neurons recorded in PPN/MRN. Top, spike raster. From top to bottom, lick right control, lick left control, lick right with perturbation, and lick left with perturbation trials. Bottom, mean spike rate. Time is aligned to the timing of the Go cue (dotted line).
E. Same as **D** for ALM neurons.
F. PPN/MRN neurons were modulated by sinusoidal modulation of the laser power. Top, laser intensity in one sinusoidal cycle (25 ms, 40 Hz, mean power: 1 mW). Middle, phase and amplitude of activity of PPM/MRN neurons at 40 Hz (by fast-Fourier transformation, of mean spike activity during the perturbation; 45 cells analyzed in Figure 7C). Circles, individual cells; black, control trials without perturbation; green, perturbation trials. Bottom, the same for ALM neurons (44 cells analyzed in Figure 7C).
G. Grand average PSTH of ALM neurons in animals expressing GtACR1 in thalamus-projecting Syn+ PPN/MRN cells (left; from 4 mice), thalamus-projecting CamKII+ PPN/MRN cells (middle; from 4 mice), Chat+ PPN/MRN cells (right; from 2 mice). Top, control trials; bottom, perturbation trials; cyan bar, laser on.
H. Example sessions with small increases in activity along **D**_**go**_ at the laser onset. Top, control trials; bottom, perturbation trials. Cyan bar, laser on. Note that an increase in activity after the Go cue is lost in the perturbation trials.
I. Quantification of change in activity after the Go cue (50 ms, top; 100 ms, bottom) along each direction. Cumulative distribution across 1000 bootstrap trials shown in Figure 7D. *P-value*, bootstrap with a null hypothesis that activity change in control trials is smaller than or equal to that in perturbation trials (left *p*-value, lick left trials; right *p*-value, lick right trials).

9 Movie S1. Related to Figure 1. Example trial with the Go cue
Top, side view of a mouse with tracking of the jaw (red), nose (blue), and tongue (green). Bottom, movement of jaw and tongue along the dorsoventral axis (a.u.). 400 Hz.

10 Movie S2. Related to Figure 6. Example trial with thalamus-projecting PPN/MRN stimulation
Same format as in Movie S1 for a trial with thalamus-projecting PPN/MRN stimulation at time 0.

11 Table S1. List of mice and conditions used for optogenetic/behavioral experiments (Related to Figures 2,3,6,8 and S8)

12 Table S2. List of mice used for anatomical experiments (Related to Figures 4 and S4)

13 Table S3. List of viruses used in this paper (related to STAR Methods)

14 Table S4. Probe sequences used for HCR. Probes were designed for CDS of *chat*, *slc17a6* and *gad1* (Related to STAR Methods)

## Figures and Tables

**KEY RESOURCES TABLE:** 

REAGENT or RESOURCE	SOURCE	IDENTIFIER
Antibodies		
Rabbit anti-RFP	Rockland Immunochemicals, Pottsdown, PA	600-401-3790
Goat anti-rabbit 555	ThermoFisher Scientific	A27039
Chicken anti-GFP	Thermo Fisher Scientific	A10262
Goat anti-chicken 488	Thermo Fisher Scientific	A11039
Mouse monoclonal anti-ChAT	Sigma	AMAb 91130
Goat anti-mouse 647	Thermo Fisher Scientific	A11039
Bacterial and Virus Strains		
AAV_retro_-Syn-iCre	Janelia viral core	Addgene #122518
AAV_retro_ -CamKII-iCre	Janelia viral core	N/A
AAV_retro_ -CamKII-GFP	Janelia viral core	N/A
AAV_retro_ -CAG-GFP	Janelia viral core	Addgene #28014
AAV_retro_-CAG-H2B::TdTomato	Janelia viral core	Addgene #116870
AAV2-hsyn-ChR2(H134R)-EYFP-WPRE	UNC vector core	N/A
AAV2/5-CamKII-hChR2(H134R)-EYFP-WPRE	UNC vector core	N/A
AAV_retro_ -CamKII-stGtACR1-FusionRed	Janelia viral core	Addgene #105679
AAV2/5-hsyn-SIO-stGtACR1-FusionRed	Janelia viral core	Addgene #105678
AAV2/1-hsyn-FLEX-ReachR-Cit	Janelia viral core	Addgene #50955
AAV2/5-EF1α-DIO-hChR2 (H134R)-mCherry	University of Pennsylvania Vector Core	N/A
Chemicals, Peptides, and Recombinant Proteins		
Muscimol-HBr	Sigma-Aldrich	G019
Wheat Germ Agglutinin, Alexa Fluor™ 555 Conjugate	Invitrogen	W32464
Red RetroBeads^TM^	Lumafluor	N/A
4-Aminopyridine (4AP)	Sigma-Aldrich	275875
CNQX DISODIUM SALT HYDRATE	Sigma-Aldrich	C239
Tetrodotoxin citrate (TTX)	Alomone Labs	T-550
Deposited Data		
Thalamus and SNr neurons data	“Maintenance of persistent activity in a frontal thalamocortical loop” *Nature***volume 545**, pages181–186 (2017)	https://doi.org/10.1038/nature22324
DCN neurons data	“A cortico-cerebellar loop for motor planning” *Nature***volume 563**, pages113–116 (2018)	http://dx.doi.org/10.6080/K0NS0S26
ALM and PPN data	This paper	Dandiarchive.org, ID:000221
Experimental Models: Organisms/Strains		
C57Bl/6J	Jackson Laboratory	JAX #000664
PV-IRES-Cre	Jackson Laboratory	JAX #017320
Ai32	Jackson Laboratory	JAX #024109
Chat-IRES-Cre	Jackson Laboratory	JAX #006410
Vglut2-IRES-Cre mice	Jackson Laboratory	JAX #028863
Sst-IRES-Cre	Jackson Laboratory	JAX #013044
VGAT-ChR2-EYFP	Jackson Laboratory	JAX #14548
Rbp4-Cre	from Charles Gerfen lab	MMRRC031125
Oligonucleotides		
Probes for HCR	This paper	N/A
Software and Algorithms		
Neurolucida software	MBF Bioscience	https://www.mbfbioscience.com/neurolucida
NeuroInfo software	MBF Bioscience, Williston, VT	https://www.mbfbioscience.com/neuroinfo
Matlab_R2020b	MathWorks	https://www.mathworks.com/products/matlab.html
SpikeGLX	Janelia Research Campus	http://billkarsh.github.io/SpikeGLX/
JRClust	James Jun and Janelia Scientific Computation	https://github.com/JaneliaSciComp/JRCLUST
KiloSort2	Marius Pachitariu	https://github.com/jamesjun/Kilosort2
DeepLabCut	Mathis et al 2018: 10.1038/s41593-018-0209-y	https://github.com/DeepLabCut/DeepLabCut

## Box

- Distinct motor cortex activity patterns underlie planning and movement initiation
- A contextual cue reorganizes motor cortex activity to initiate planned movements
- Midbrain neurons transmit information to cortex via thalamus to release movements
- Midbrain activity is causal for reorganizing cortical activity and releasing movements
