# Supplementary figures and images for: A midbrain-thalamus-cortex circuit reorganizes cortical dynamics to initiate movement
Source: Cell. Author manuscript; Available in PMC 2023 Mar 17. (PMC8990337; doi:10.1016/j.cell.2022.02.006)

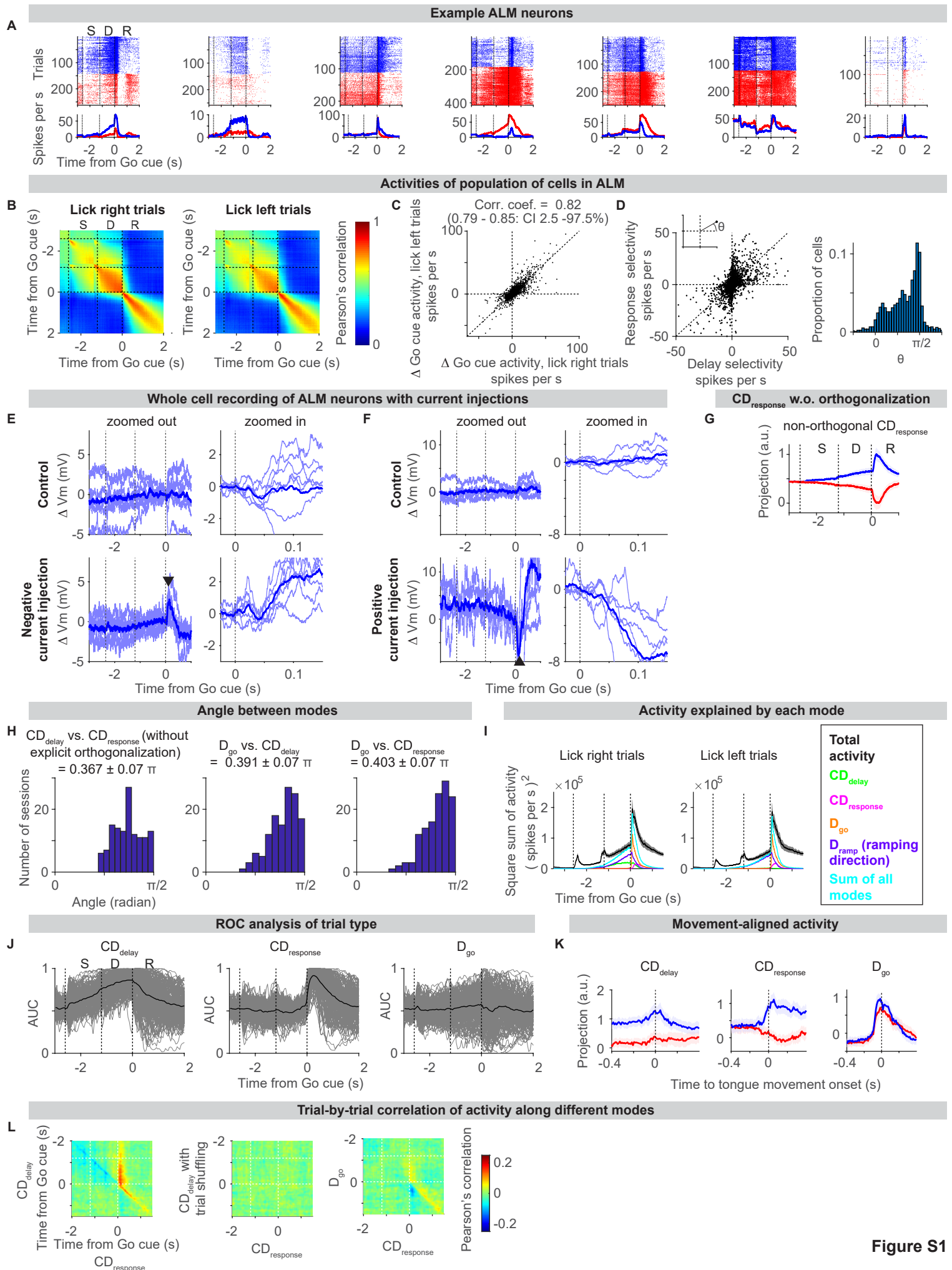

**Figure S1**

Supplement: 1 — Figure S1. Related to Figure 1. Activity underlying motor planning and movement initiation in ALM A. Example neurons in ALM. Top, spike raster. Bottom, mean spike rate. Blue, correct lick right trials; red, correct lick left trials. Time is aligned to the onset of the Go cue. Dashed lines separate behavioral epochs. S, sample epoch; D, delay epoch; R, response epoch. B. Pearson’s correlation of the population activity vector is low between time points before and after the Go cue. Dashed lines separate behavioral epochs. C. Go cue activity (mean spike rate after the Go cue – mean spike rate before the Go cue; 100 ms window) is similar between lick right (x-axis) and left (y-axis) trials. This is consistent with non-selective Dgo. Circles, individual neurons in ALM (5136 neurons). D. Selectivity during the delay and response epochs is not consistent. Left, the relationship between delay selectivity (mean selectivity during the last 600 ms of the delay epoch) and response selectivity (mean selectivity during the first 400 ms of the response epoch). Circles, individual neurons in ALM (5136 neurons). Inset, the definition of θ (angle in a polar coordinate). Right, histogram of θ across neurons. θ ~ Ꮐ/4 indicates similar selectivity during the delay and response epoch, whereas θ ~ 0 or π/2 indicates selectivity is strong only in the delay or response epoch, respectively. E. Increase in conductance of ALM neurons after the Go cue. Membrane potential (Vm) of ALM neurons during the tactile task without (top) or with (bottom) negative current injection in correct lick right trials. Change in Vm (ΔVm) from that before the Go cue (100 ms window) is shown. Thin lines, each neuron. Thick line, mean. Results in correct lick left trials were similar (data not shown). We manipulated the membrane potential during whole-cell recordings in ALM to observe changes in synaptic conductances around the movement initiation. To enhance either excitatory or inhibitory synaptic potentials, we i [file NIHMS1784450-supplement-1.pdf]

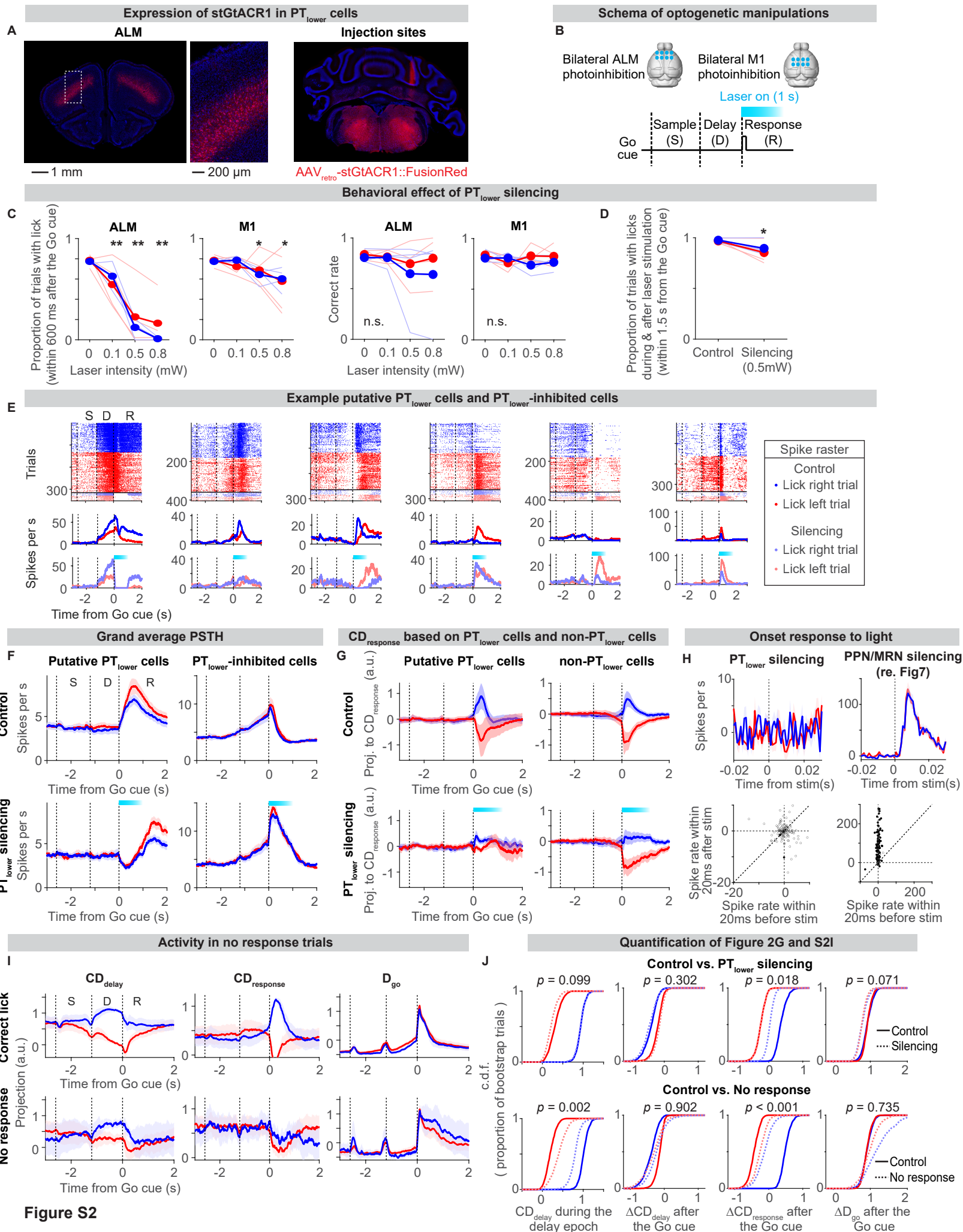

**Figure S2**

Supplement: 2 — Figure S2. Related to Figure 2. Attenuated CDresponse but intact Dgo with silencing of ALM output A. Coronal brain sections showing bilateral expression of stGtACR1 in PTlower cells. Left, ALM. Right, injection sites in the medulla (see Supplementary Table 1 for coordinates). Red, the fluorescence of FusionRed fused to stGtACR1. Blue, DAPI. B. Schema of PTlower silencing experiments. C. Calibration of laser power. Behavioral effect of PTlower silencing with different laser intensities. Thin lines, individual animals (n = 4 mice). Thick line, mean. PTlower silencing in ALM decreased the proportion of trials with lick without affecting correct rate (probability to lick the correct direction). Because of the significant behavioral effect in ALM with modest effect in M1, we selected 0.5 mW for the rest of the experiments. *: p < 0.05; **; p < 0.01 (Bootstrap with Bonferroni correction for multiple comparisons; null-hypothesis is that the proportion of lick or correct rate in control trials is lower than or equal to those in silencing trials). D. PTlower silencing resulted in loss of lick within 0.6 s after the Go cue (Figure 2C), whereas the probability to lick within 1.5 s after the Go cue (laser is on for 1s after the Go cue) was less affected (p = 0.003, bootstrap). This indicates that licking recovers after the laser stimulation (e.g., Figure 2B). Note that mice lick the correct direction (Figure S2C, correct rate). Same n = 3 mice as in Figure 2C. E. Example putative PTlower cells (cells with a significant decrease in activity during the silencing; left three cells) and PTlower-inhibited cells (cells with a significant increase in activity during the silencing; right three cells). Top, raster; middle and bottom, mean spike rates of trials with and without PTlower silencing. Blue, mean of all lick right trials (including correct, incorrect, and no lick trials); red, mean of all lick left trials; cyan bar, laser on. F. Grand average PSTH of putative PTlower cells (n [file NIHMS1784450-supplement-2.pdf]

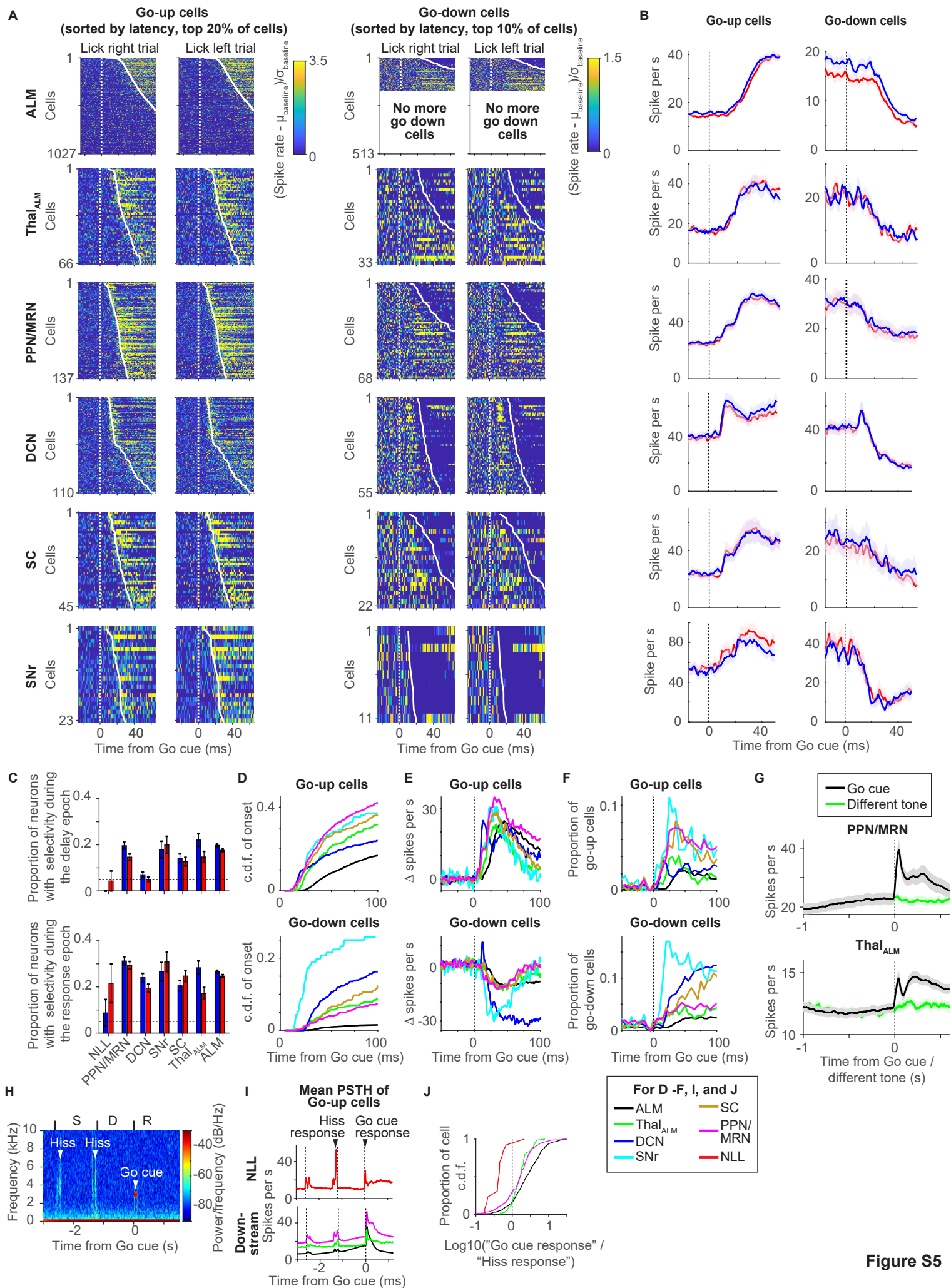

**Figure S5**

Supplement: 5 — Figure S5. Related to Figure 5. Latency to the Go cue across brain areas. A. Spike rates of neurons sorted by their latency to the Go cue in each brain area. From left to right: neurons with an increase in spike rate (go-up cells) in lick right trials and lick left trials and neurons with a decrease in spike rate (go-down cells) in lick right trials and lick left trials. The top 20% of cells and 10% of cells are shown for go-up and go-down cells, respectively. Spike rates were normalized by the spike rate before the Go cue (100 ms) and shown as a heatmap. See Methods for the number of cells recorded in each area. B. Grand average PSTH of the go-up and go-down cells. Line, grand average; shading, S.E.M. (bootstrap); blue, lick right trial; red, lick left trial. C. Proportion of neurons with selectivity during the delay (top) or response (bottom) epoch in each area. Blue, lick right trial; red, lick left trial; error bar, S.E.M. (bootstrap); dashed line, chance level (p = 0.05 as selectivity was defined by ranksum test with α = 0.05). D. Same as Figure 5D, but a broader time-window is shown. Each color indicates a different brain area (box below F). Fraction of Go-up cells (<15 ms latency) is 4.5 % and Go-down cells is 0.15 % in PPN/MRN. E. Overlay of grand average PSTH of the go-up and go-down cells. The mean spike rate before the Go cue (100 ms window) was subtracted. F. Proportion of neurons with significant (ranksum test, p < 0.01) increase or decrease in activity after the Go cue (compared to before the Go cue; 100 ms window) at each time point (10 ms bin). G. Grand average PSTH of neurons in PPN/MRN (top) and thalALM (bottom) in trials with the Go cue (black) or a different tone (green). Related to Figure 5G. In both areas, neurons specifically responded to the Go cue. Both lick right and left trials were pooled. H. Spectrogram of sound recorded during the task. Note the signals containing a broad frequency spectrum at the start and end of the sample epoch. This [file NIHMS1784450-supplement-5.pdf]

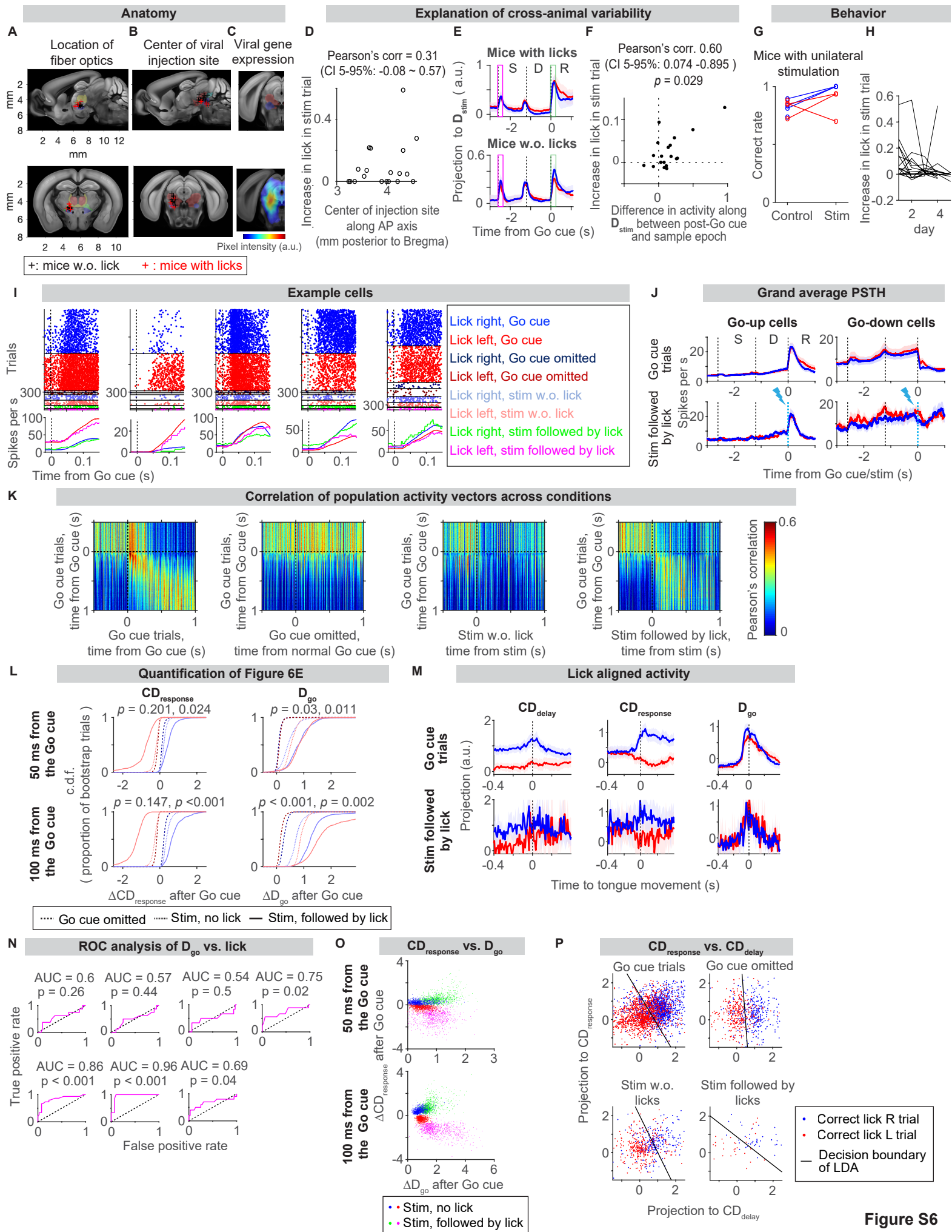

**Figure S6**

Supplement: 6 — Figure S6. Related to Figure 6. Stimulation of thalamus-projecting PPN/MRN triggers licking responses. A. Anatomical location of fiber optics in the thalamus. Each region filled with a color indicates a different thalamic nucleus. Red, MD; yellow, IL; green, VAL; blue, VM. After recordings, brains were imaged and registered to Allen CCF (n = 20 mice). Black cross, tips of fiber optics in mice without stimulation-triggered lick; red cross, the same in mice with stimulation-triggered licks. Top, sagittal view; bottom, coronal view (AP −1.38 mm from Bregma). B. Anatomical location of the center of virus injection in PPN/MRN. Each region filled with a color indicates a different midbrain nucleus. Red, MRN; blue, PPN; green, cuneiform nucleus. Same animals as analyzed in A. Top, sagittal view; bottom, coronal view (AP −3.92 mm from Bregma). C. YFP (conjugated to ChR2) signal around the injection site (mean of 3 mice). Signal intensity is shown in the colormap. The injection site has the strongest signal (red). Weaker signals (cyan) are projections. Top, Allen CCF; bottom, coronal view (AP −4.1 mm from Bregma). D. Anatomical location of viral injection along anterior-posterior (AP) axis and increase in lick in stim trials (probability to lick in stimulation trials – probability to lick in Go cue omitted trials). There is a trend that posterior injection results in a higher probability of stimulation-triggered licks. We see a similar trend with the GtACR experiment as well (Figures S8A–S8B; HI211 and 215 reduced licks with the weaker 0.25mW laser power). CI, confidence interval based on bootstrap. E. Explanation of cross-animal variability in the probability of stimulation-triggered licks based on ALM activity. We defined a stimulation direction (Dstim), which distinguishes activity with or without stimulation in ALM (Methods). Activities in Go cue trials projected along Dstim are different between mice with (top) or without (bottom) stimulation-triggered licks. In mice wi [file NIHMS1784450-supplement-6.pdf]

# Muscimol infusion in PPN/MRN

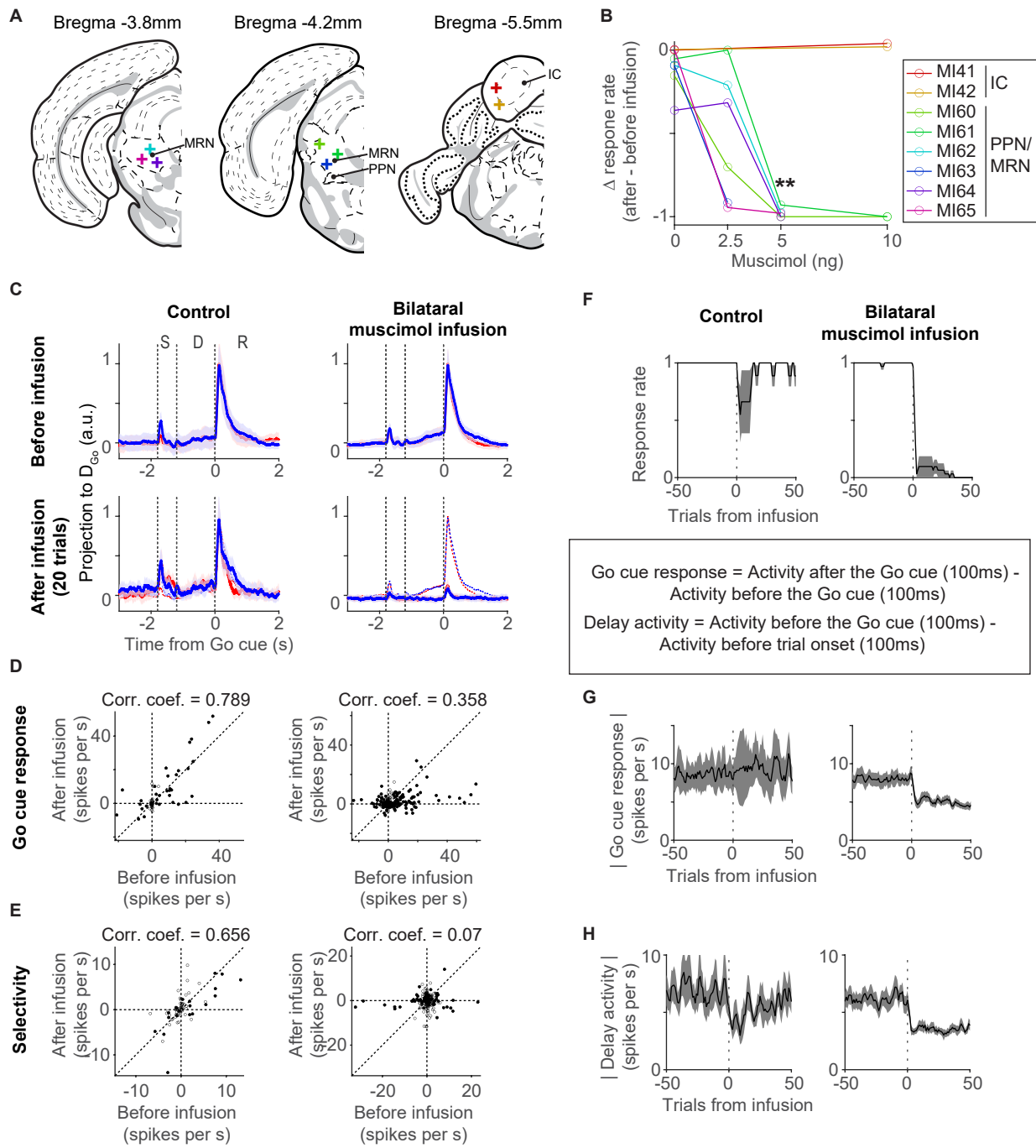

# Fake Go cue experiment

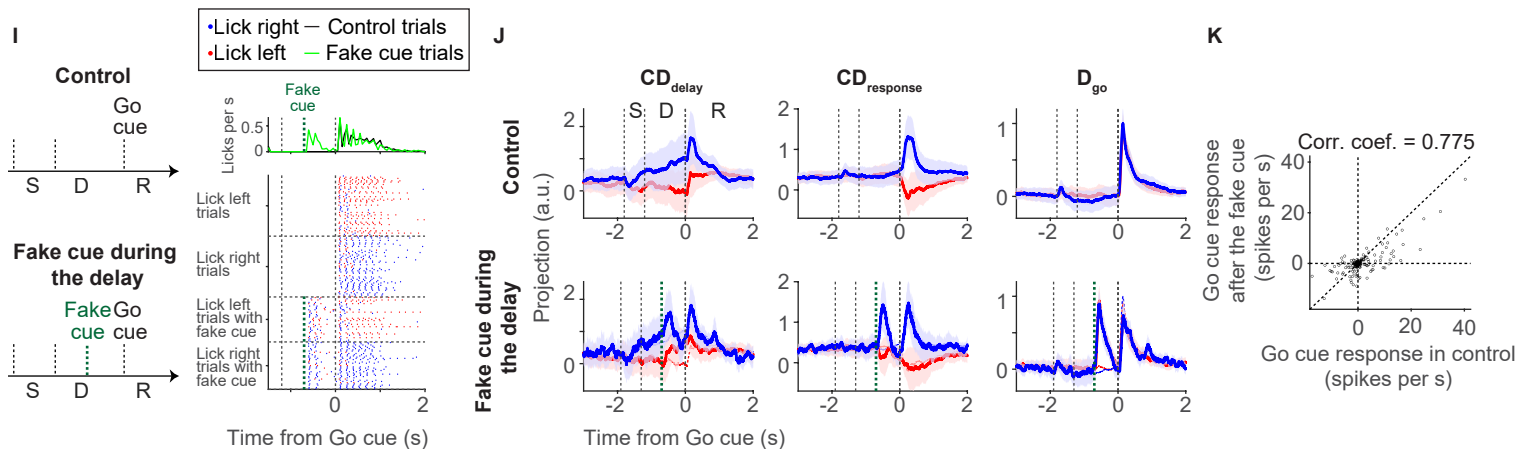

Figure S7

Supplement: 7 — Figure S7. Related to Figure 7. Muscimol-mediated silencing of PPN/MRN A. Infusion location. One hemisphere is shown, although infusions were bilateral. Crosses, locations of the cannula confirmed by post-hoc histology (n = 8 mice; colors correspond to the animals shown in B). B. Behavioral effect of bilateral muscimol infusion. All animals with PPN/MRN infusion showed significant reduction in response rate with <= 5 ng muscimol. Muscimol was dissolved in cortex buffer. Control, infusion of cortex buffer without muscimol. **: p = 0.00012 (two-tailed paired t-test comparing 0 vs 5 ng muscimol, n = 6 animals). C. Activity in ALM along Dgo before and after infusion. The Go cue response was significantly attenuated after bilateral muscimol infusion. The first 20 trials after infusion were analyzed (same in D and E). Line, mean; shading, S.E.M. n = 73, 250 cells in 6 PPN/MRN infusion mice (control, muscimol, respectively; same in D, E, G and H). D. The amplitude of the Go cue response before and after the infusion for single neurons. Circle, individual neuron. Filled circle, cell with significant go cue response before infusion (p < 0.05, signrank test). E. Delay selectivity before and after the infusion. In addition to the loss of Go cue response, delay activity (non-selective ramping activity and selectivity during the delay epoch) in ALM became weaker after muscimol infusion (E and H). The attenuated delay activity is likely due to lack of water reward during and after the infusion protocol. After an infusion we waited for 5 minutes for muscimol or control cortex buffer to diffuse, during which animals did not receive water reward. Low expected reward and motivational state attenuate delay activity (Roesch and Olson, 2003; Allen et al., 2019). Consistent with this idea, even under control conditions (i.e. no muscimol), delay activity dropped after infusion, and gradually recovered after water consumption during the task (H). After bilateral muscimol infusion delay act [file NIHMS1784450-supplement-7.pdf]
