# Supplementary material for: A midbrain-thalamus-cortex circuit reorganizes cortical dynamics to initiate movement
Source: Cell. Author manuscript; Available in PMC 2023 Mar 17. (PMC8990337; doi:10.1016/j.cell.2022.02.006)
Supplement: 3 — Figure S3. Related to Figure 3. Thalamic and mibrain activity. Latency to Go cue across brain areas (B-D) are similar (latency in PPN/MRN < latency in thalALM < latency in ALM) in a different task: auditory delayed-response task (A). Data in E is based on the tactile task (related to Figures 3D–3F). A. Auditory delayed-response task. Tones (3 or 12 kHz) instead of tactile cues were presented during the sample epoch to instruct lick direction. Go cue is 6 kHz FM sound (Methods). B. Recording in the thalamus (top) and midbrain (bottom). Each region filled with color indicates different thalamic or midbrain nuclei. White contour, thalALM. Black dots, location of individual recorded neurons in the Allen common coordinate framework (CCF). Green, neurons with < 20 ms (top; in the thalamus) or <15 ms (bottom; in the midbrain) latency to the Go cue. C. Cumulative distribution (c.d.f.) of latency to the Go cue in ALM and M1. Latency (mean ± S.E.M.; time point in which 1% of recorded cells increase activity): 21.1 ± 0.5 ms (ALM; n = 5072 units) and 20.3 ± 4.9 ms (M1; n = 674 units). P = 0.402 (bootstrap with a null hypothesis that the latency in M1 is equal to or faster than ALM). D. c.d.f. of latency to the Go cue across brain areas. Latency (mean ± S.E.M.; time point in which 1% of recorded cells increase activity): 21.1 ± 0.5 ms (ALM; n = 5072 units); 16.0 ± 1.5 ms (thalALM; n = 607 units); 10.1 ± 0.8 ms (SC; n = 1145 units); and 7.2 ± 0.5 ms (PPN/MRN; n = 1560 units). E. Distribution of thalamic neurons with decreased delay activity during ALM silencing (left, schema). Note that neurons within thalALM (white contour) were strongly silenced, consistent with the strong excitatory drive from ALM to thalALM (Guo et al., 2017). Top, location of individual recorded neurons in the Allen CCF (black). Neurons with more than 50 and 75% reduction in spike rates during ALM silencing (green and red, respectively). Bottom, the density of neurons with more than 50% reduction in spike ra [file NIHMS1784450-supplement-3.pdf]

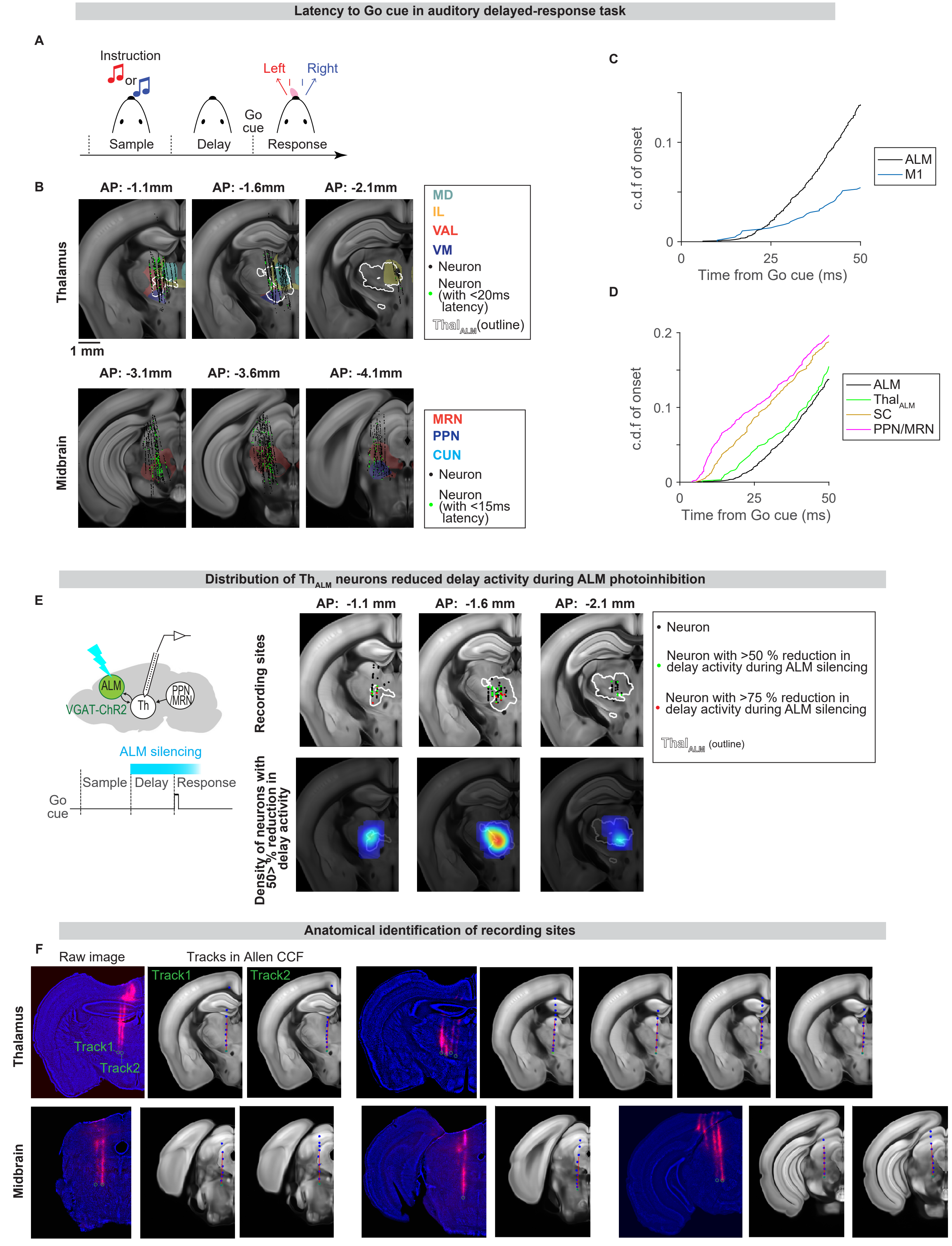

Latency to Go cue in auditory delayed-response task
A
Instruction
or
Left
Right
Go cue
Sample
Delay
Response
B
AP: -1.1mm
AP: -1.6mm
AP: -2.1mm
Thalamus
MD
IL
VAL
VM
Neuron
Neuron (with <20ms latency)
Thal ALM (outline)
1 mm
AP: -3.1mm
AP: -3.6mm
AP: -4.1mm
Midbrain
MRN
PPN
CUN
Neuron
Neuron (with <15ms latency)
C
c.d.f of onset
0.1
0
0
25
50
Time from Go cue (ms)
ALM
M1
D
c.d.f of onset
0.2
0.1
0
0
25
50
Time from Go cue (ms)
ALM
Thal ALM
SC
PPN/MRN
Distribution of Th ALM neurons reduced delay activity during ALM photoinhibition
E
ALM
VGAT-ChR2
Th
PPN /MRN
ALM silencing
Sample
Delay
Response
Go cue
Recording sites
AP: -1.1 mm
AP: -1.6 mm
AP: -2.1 mm
Neuron
Neuron with >50 % reduction in delay activity during ALM silencing
Neuron with >75 % reduction in delay activity during ALM silencing
Thal ALM (outline)
Density of neurons with 50>% reduction in delay activity
Anatomical identification of recording sites
F
Raw image
Tracks in Allen CCF
Thalamus
Track1
Track2
Midbrain

Figure S3
