# Supplementary material for: A midbrain-thalamus-cortex circuit reorganizes cortical dynamics to initiate movement
Source: Cell. Author manuscript; Available in PMC 2023 Mar 17. (PMC8990337; doi:10.1016/j.cell.2022.02.006)
Supplement: 4 — Figure S4. Related to Figure 4. Neurons projecting to thalALM. Characterization of thalALM-projecting neurons based on retrograde (A-C) and anterograde labeling (D-F). In addition, we confirmed that most thalamus-projecting PPN/MRN neurons are glutamatergic using the fluorescent in situ hybridization (FISH; G, H), immunostaining (I, J), and acute slice recording (K-Q). A. Quantification of retrogradely labeled cells in an animal with retrobeads injection in thalALM. The total pixel intensities of retrobeads signal in midbrain/hindbrain areas are shown. Blue, contralateral hemisphere; red, ipsilateral hemisphere to the injection site. Original images of this sample are reported in (Guo et al., 2017). B. Quantification of retrogradely labeled cells in an animal with AAVretro injection in thalALM. The number of labeled cells in midbrain/hindbrain areas are shown. Blue, contralateral hemisphere; red, ipsilateral hemisphere to the injection site. Error bar, standard deviation (n = 2 mice). Some inconsistencies between the retrobeads and AAVretro are caused by known viral tropism (e.g., weak labeling of SNr by AAVretro (Tervo et al., 2016)) and a spread of AAVretro at the injection site beyond thalALM (Figure S4C). C. Distribution of retrogradely labeled cells in an animal with AAVretro injection in thalALM. Images are registered to Allen common coordinate framework (CCF). AP, relative to Bregma. Heatmap indicates the number of labeled cells per voxel (size: 10 × 10 × 1000 μm). D. Anterograde labeling from distinct subcortical areas to thalALM. Images are registered to Allen CCF. AP, relative to Bregma. Unlike PPN/MRN projection (Figure 4A), projections of these structures are more localized. E. Quantification of anterograde labeling from PPN/MRN to different thalamic nuclei within thalALM. Projection is stronger to the ipsilateral hemisphere. F. Similarity of axonal projection pattern from each subcortical area (i.e., pixel intensities in Figures 4A and S4D), and the dis [file NIHMS1784450-supplement-4.pdf]

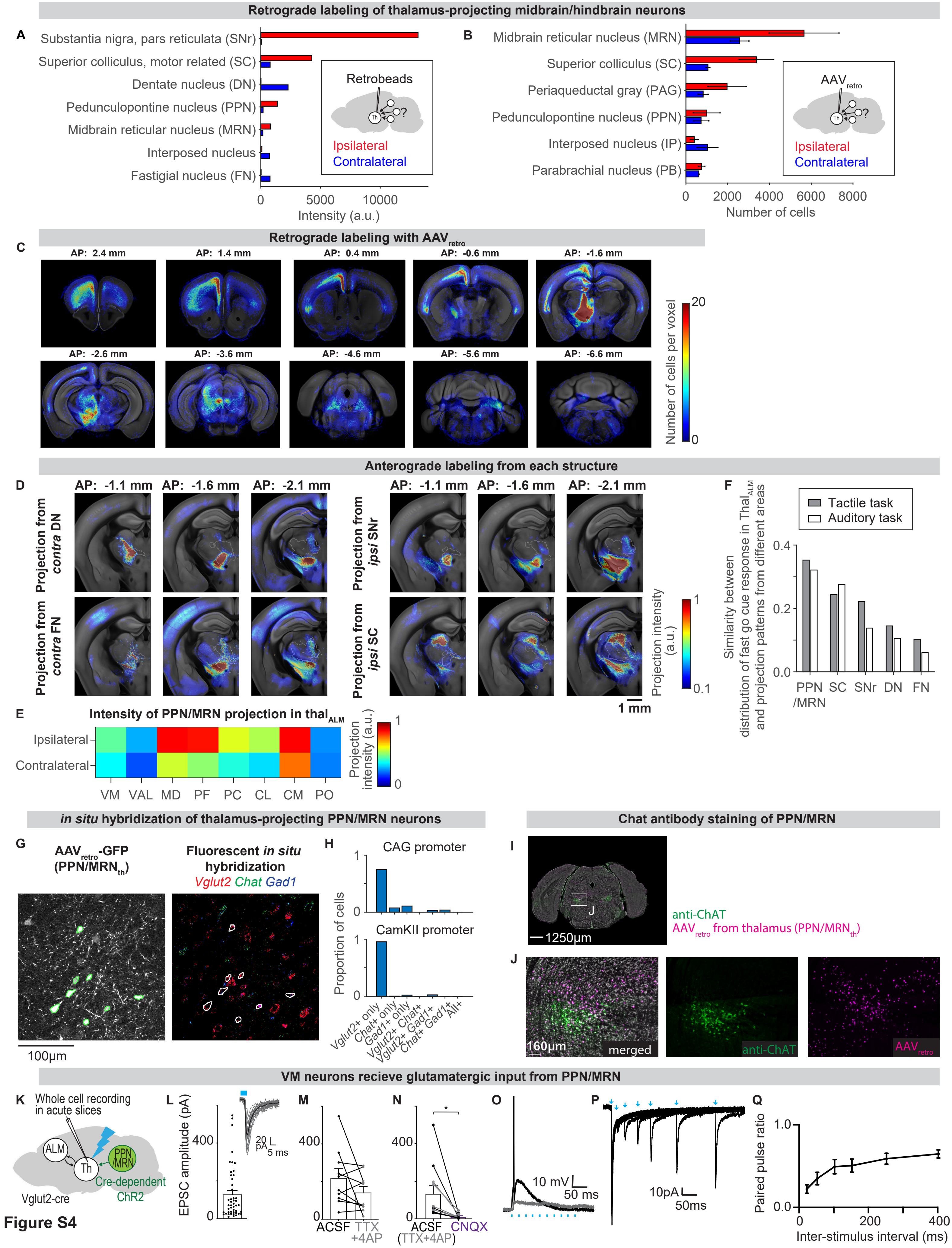

Retrograde labeling of thalamus-projecting midbrain/hindbrain neurons
A
Substantia nigra, pars reticulata (SNr)
Superior colliculus, motor related (SC)
Dentate nucleus (DN)
Pedunculopontine nucleus (PPN)
Midbrain reticular nucleus (MRN)
Interposed nucleus
Fastigial nucleus (FN)
0 5000 10000
Intensity (a.u.)
Retrobeads
Th
Ipsilateral
Contralateral
B
Midbrain reticular nucleus (MRN)
Superior colliculus (SC)
Periaqueductal gray (PAG)
Pedunculopontine nucleus (PPN)
Interposed nucleus (IP)
Parabrachial nucleus (PB)
0 2000 4000 6000 8000
Number of cells
AAVretro
Ipsilateral
Contralateral
Retrograde labeling with AAVretro
C
AP: 2.4 mm
AP: 1.4 mm
AP: 0.4 mm
AP: -0.6 mm
AP: -1.6 mm
AP: -2.6 mm
AP: -3.6 mm
AP: -4.6 mm
AP: -5.6 mm
AP: -6.6 mm
Number of cells per voxel
20
0
Anterograde labeling from each structure
D
AP: -1.1 mm
AP: -1.6 mm
AP: -2.1 mm
Projection from contra DN
Projection from contra FN
Projection from ipsi SNr
Projection from ipsi SC
Projection intensity (a.u.)
1
0.1
1 mm
E
Intensity of PPN/MRN projection in thalALM
Ipsilateral
Contralateral
VM VAL MD PF PC CL CM PO
Projection intensity (a.u.)
1
0
F
Tactile task
Auditory task
Similarity between distribution of fast go cue response in ThalALM and projection patterns from different areas
0.4
0.2
0.0
PPN /MRN SC SNr DN FN
in situ hybridization of thalamus-projecting PPN/MRN neurons
G
AAVretro-GFP (PPN/MRNth)
Fluorescent in situ hybridization
Vglut2 Chat Gad1
100µm
H
CAG promoter
CamKII promoter
Proportion of cells
1
0
Vglut2+ only
Chat+ only
Gad1+ only
Vglut2+ Chat+
Vglut2+ Gad1+
Chat+ Gad1+
All+
Chat antibody staining of PPN/MRN
I
J
1250µm
anti-ChAT
AAVretro from thalamus (PPN/MRNth)
160µm
merged
anti-ChAT
AAVretro
VM neurons recieve glutamatergic input from PPN/MRN
K
Whole cell recording in acute slices
ALM
Th
PPN /MRN
Cre-dependent ChR2
Vglut2-cre
L
EPSC amplitude (pA)
400
0
20 pA
5 ms
M
400
0
ACSF
TTX +4AP
N
*
400
0
ACSF (TTX+4AP)
CNQX
O
10 mV
50 ms
P
10pA
50ms
Q
Paired pulse ratio
1.0
0.0
0 200 400
Inter-stimulus interval (ms)

Figure S4
