# Supplementary material for: A midbrain-thalamus-cortex circuit reorganizes cortical dynamics to initiate movement
Source: Cell. Author manuscript; Available in PMC 2023 Mar 17. (PMC8990337; doi:10.1016/j.cell.2022.02.006)
Supplement: 8 — Figure S8. Related to Figure 7. Perturbation of thalamus-projecting PPN/MRN neurons blocks movement initiation A. Anatomical location of the tips of fiber optics (cross) in PPN/MRN (n = 4 mice; HI## are animal names). Sagittal view. Each region filled with a color indicates a different midbrain nucleus. All data in Figure S8 except G is based on animals injected with AAVretro-CamKII-Cre in the thalamus. B. Coronal view. Same brains as analyzed in A. AP −4.34 mm from Bregma. C. Raster plot of lick timing in all animals. 0.25 and 1 mW indicate laser power used for perturbation. Cyan box, laser on. Behavioral effects were stronger in HI211 and 215. D. Example neurons recorded in PPN/MRN. Top, spike raster. From top to bottom, lick right control, lick left control, lick right with perturbation, and lick left with perturbation trials. Bottom, mean spike rate. Time is aligned to the timing of the Go cue (dotted line). E. Same as D for ALM neurons. F. PPN/MRN neurons were modulated by sinusoidal modulation of the laser power. Top, laser intensity in one sinusoidal cycle (25 ms, 40 Hz, mean power: 1 mW). Middle, phase and amplitude of activity of PPM/MRN neurons at 40 Hz (by fast-Fourier transformation, of mean spike activity during the perturbation; 45 cells analyzed in Figure 7C). Circles, individual cells; black, control trials without perturbation; green, perturbation trials. Bottom, the same for ALM neurons (44 cells analyzed in Figure 7C). G. Grand average PSTH of ALM neurons in animals expressing GtACR1 in thalamus-projecting Syn+ PPN/MRN cells (left; from 4 mice), thalamus-projecting CamKII+ PPN/MRN cells (middle; from 4 mice), Chat+ PPN/MRN cells (right; from 2 mice). Top, control trials; bottom, perturbation trials; cyan bar, laser on. H. Example sessions with small increases in activity along Dgo at the laser onset. Top, control trials; bottom, perturbation trials. Cyan bar, laser on. Note that an increase in activity after the Go cue is lost in the perturbation [file NIHMS1784450-supplement-8.pdf]

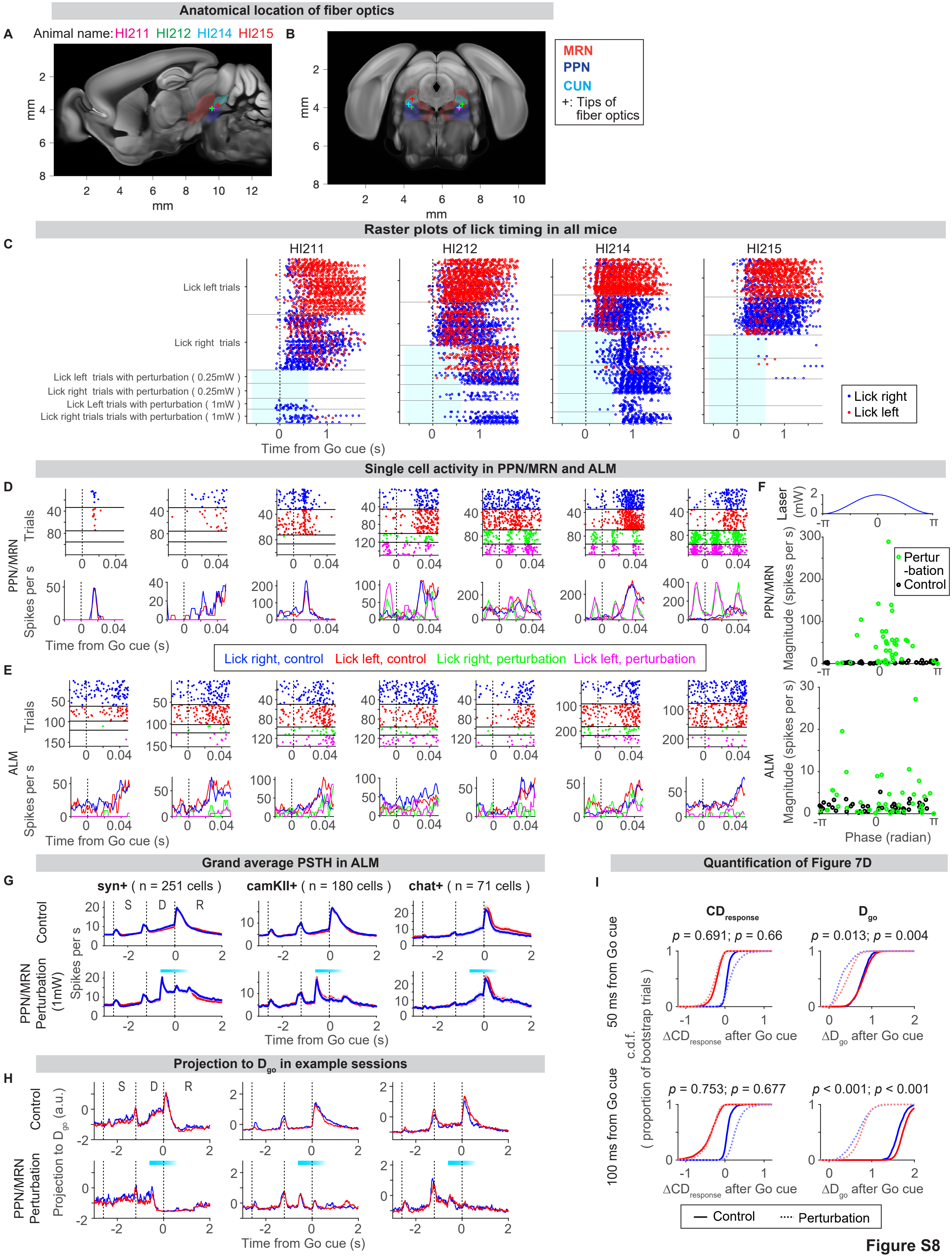

Anatomical location of fiber optics
A
Animal name: HI211 HI212 HI214 HI215
B
MRN
PPN
CUN
+: Tips of fiber optics
mm
Raster plots of lick timing in all mice
C
HI211 HI212 HI214 HI215
Lick left trials
Lick right trials
Lick left trials with perturbation (0.25mW)
Lick right trials with perturbation (0.25mW)
Lick Left trials with perturbation (1mW)
Lick right trials trials with perturbation (1mW)
Time from Go cue (s)
Lick right
Lick left
Single cell activity in PPN/MRN and ALM
D
PPN/MRN
Trials
Spikes per s
Time from Go cue (s)
Lick right, control Lick left, control Lick right, perturbation Lick left, perturbation
E
ALM
F
Laser (mW)
Pertur -bation
Control
PPN/MRN Magnitude (spikes per s)
ALM Magnitude (spikes per s)
Phase (radian)
Grand average PSTH in ALM
G
syn+ (n = 251 cells)
camKII+ (n = 180 cells)
chat+ (n = 71 cells)
S D R
Control
PPN/MRN Perturbation (1mW)
Spikes per s
Time from Go cue (s)
Projection to D_go in example sessions
H
Control
PPN/MRN Perturbation
Projection to D_go (a.u.)
Time from Go cue (s)
Quantification of Figure 7D
I
CD_response
D_go
p = 0.691; p = 0.66
p = 0.013; p = 0.004
p = 0.753; p = 0.677
p < 0.001; p < 0.001
50 ms from Go cue
100 ms from Go cue
c.d.f. (proportion of bootstrap trials)
ΔCD_response after Go cue
ΔD_go after Go cue
Control
Perturbation

Figure S8
