# Supplementary material for: A midbrain-thalamus-cortex circuit reorganizes cortical dynamics to initiate movement
Source: Cell. Author manuscript; Available in PMC 2023 Mar 17. (PMC8990337; doi:10.1016/j.cell.2022.02.006)
Supplement: 11 — Table S1. List of mice and conditions used for optogenetic/behavioral experiments (Related to Figures 2,3,6,8 and S8) [file NIHMS1784450-supplement-11.pdf]

Figure	Purpose	Genotype, number of mice	Viral injection or cannula sites	Photoinhibition method
Figure 2	PT_{lower} silencing	C57Bl/6J<br>4 mice	AAV_{retro}-CamKII-stGtACR1<br>(**Bilateral** injection, coordinate: Bregma AP -6.65mm, ML +/-1.25mm , DV 4.5mm, 200 nl)	Clear skull prep<br>Photoinhibition 0.5 mW (Bilateral ALM, 8 spots)
Figure 3D-3F	Silencing of ALM during Th recording	PV-IRES-Cre x Ai32<br>2 mice	N/A	Clear skull prep<br>Photoinhibition 1.5mW (Unilateral ALM)
Figure 6	Stimulation of hsyn+ Th-projecting PPN/MRN neurons	C57Bl/6J<br>4 mice	AAV2-hsyn-ChR2-EYFP<br>(**Unilateral** injection, coordinate: Lambda AP +0.2 ~ -0.37mm, ML 1.25mm, DV 2.5mm and/or 3.0mm, 100nl each)	Fiber optics coordinate: Bregma AP -1.5 ~ -1.8mm, ML 0.9mm (**left only**), DV 3.7mm<br><br>Doric lenses: TFC-200/245-0.37_5mm_TS2.C45
		C57Bl/6J<br>16 mice	AAV2-hsyn-ChR2-EYFP<br>(**Bilateral** injection, coordinate: Lambda AP +0.2 ~ -0.37mm, ML +/-1.25mm, DV 2.5mm and/or 3.0mm, 100nl each)	
Figure 7	Perturbation of hsyn+ Th-projecting PPN/MRN neurons	C57Bl/6J<br>4 mice	AAV_{retro}-Syn-iCre<br>(**Bilateral** injection, coordinate: Bregma AP -1.8mm, ML +/-0.9mm, DV 3.9mm, 100nl)<br><br>+ AAV2/5-hsyn-SIO-stGtACR1-FusionRed<br>(**Bilateral** injection, coordinate: Lambda AP 0.3mm, ML +/-1.25mm, DV 3.0mm, 200nl)	Fiber optics coordinate: Lambda AP 0.325mm, ML +/-1.3mm (**bilateral**), DV 2.7mm<br><br>Doric lenses: TFC-200/245-0.37_5mm_TS2.C45
Figure 7B	Perturbation of CamKII+ Th-projecting PPN/MRN neurons	C57Bl/6J<br>4 mice	AAV_{retro}-CamKII-iCre<br>(**Bilateral** injection, coordinate: Bregma AP -1.8mm, ML +/-0.9mm, DV 3.9mm, 100nl)<br><br>+ AAV2/5-hsyn-SIO-stGtACR1-FusionRed<br>(**Bilateral** injection, coordinate: Lambda AP 0.3mm, ML +/-1.25mm, DV 3.0mm, 200nl)	Fiber optics coordinate: Lambda AP 0.325mm, ML +/-1.3mm (**bilateral**), DV 2.7mm<br><br>Doric lenses: TFC-200/245-0.37_5mm_TS2.C45
Figure 7B	Perturbation of Chat+ PPN/MRN neurons	Chat-IRES-Cre<br>2 mice	AAV2/5-hsyn-SIO-stGtACR1-FusionRed<br>(**Bilateral** injection, coordinate: Lambda AP 0.3mm, ML +/-1.25mm, DV 3.0mm, 200nl)	Fiber optics coordinate: Lambda AP 0.325mm, ML +/-1.3mm (**bilateral**), DV 2.7mm<br><br>Doric lenses: TFC-200/245-0.37_5mm_TS2.C45
Figure S8A-H	PPN/MRN silencing	C57Bl/6J<br>8 mice	Guide cannula:<br>Lambda AP 0.6mm, ML +/-1mm, DV 2.0mm<br><br>Internal cannula:<br>1.5mm protrusion from the guide cannula (target depth: DV3.5mm)	

Table S1. List of mice and conditions used for optogenetic/behavioral experiments (Related to Figures 2,3,6,8 and S8)
