# Supplementary material for: A midbrain-thalamus-cortex circuit reorganizes cortical dynamics to initiate movement
Source: Cell. Author manuscript; Available in PMC 2023 Mar 17. (PMC8990337; doi:10.1016/j.cell.2022.02.006)
Supplement: 12 — Table S2. List of mice used for anatomical experiments (Related to Figures 4 and S4) [file NIHMS1784450-supplement-12.pdf]

Figure	Purpose	Genotype, number of mice	virus or tracer injections
Figure S4A	Retrograde labeling from Th using Retrobeads	C57Bl/6J<br>2 mice	Red RetroBeads<br>(VM; Bregma AP -1.5, ML 0.85, DV 4.1 mm, 50 nl)<br>Data from Guo et al. 2017
Figure S4B and S4C	Retrograde labeling from Th using AAV_{retro}	C57Bl/6J<br>2 mice	AAV_{retro}-CAG-H2B::TdTomato<br>(VM left, bregma AP -1.8, ML 0.9, DV 3.9 mm, 100 nl)
Figure 4A	Anterograde labeling from PPN/MRN	C57Bl/6J<br>4 mice	AAV2-hsyn-ChR2-EYFP<br>(**Unilateral** injection, coordinate: Lambda AP +0.2 ~ -0.37mm, ML 1.25mm, DV 2.5mm and/or 3.0mm, 100nl each)
Figure S4D	Anterograde labeling from other thalamic projecting structures	C57Bl/6J	Data from Gao et al.
Figure 4C *1	Labeling of PPN/MRN neurons	C57Bl/6J<br>2 mice	AAV2/1-CamKII-hChR2::;EYFP<br>(PPN left, lambda AP 0.37, ML 1.2, DV 2.5 and 3.0 mm, 100 nl each)
Figure 4D *2	Labeling of Th-projecting PPN/MRN neurons and $thal_{ALM}$	C57Bl/6J<br>2 mice	AAV_{retro}-CamKII-iCre<br>(VM left, bregma AP -1.5, ML 0.9, DV 4.1 mm, 100 nl)<br><br>+ AAV2/1-hsyn-FLEX-ReaChR::Citrine<br>(PPN left, lambda AP 0.37, ML 1.2, DV 2.5 and 3.0 mm, 100 nl each)<br><br>+ WGA-Alexa555<br>(ALM left, bregma AP 2.5, L 1.5, DV 0.4 and 0.8mm 50nl each)
Figure 4F	Labeling of Th-projecting PPN/MRN neurons and $thal_{ALM}$	C57Bl/6J	AAV_{retro}-CAG-Cre<br>(VM left, bregma AP -1.5, ML 0.8, DV 4.2 mm, 100 nl)<br><br>+ AAV2/8-EF1α-DIO-hChR2(H134R)-mCherry<br>(PPN left, bregma AP -4.7, ML 1.2, DV 3.5, 100 nl)<br><br>+ AAV_{retro}-CAG-EGFP<br>(ALM left, bregma AP 2.5, L 1.8, DV 0.5 and 0.8mm 60nl each)
Figure S4G and S4H	HCR	C57Bl/6J<br>2 mice	AAV_{retro}-CamKII-GFP or<br>AAV_{retro}-CAG-GFP<br>(VM left,bregma AP -1.8, ML 0.9, DV 3.9 mm, 100 nl)

Table S2. List of mice used for anatomical experiments (Related to Figures 4 and S4)
All reagents were introduced into the left hemisphere. After virus injections, expression was allowed for more than two weeks before perfusion.

*1: AAV2/2-hsyn-ChR2-EYFP resulted in similar results (i.e., strong projection both in thalamus and medulla; data not shown)
*2: AAV_{retro}-hsyn-Cre resulted in similar results (i.e., projection in thalamus without projection in medulla; data not shown).
