# Supplementary material for: A midbrain-thalamus-cortex circuit reorganizes cortical dynamics to initiate movement
Source: Cell. Author manuscript; Available in PMC 2023 Mar 17. (PMC8990337; doi:10.1016/j.cell.2022.02.006)
Supplement: 13 — Table S3. List of viruses used in this paper (related to STAR Methods) [file NIHMS1784450-supplement-13.pdf]

ID	Virus name	Titer (vg/ml)	Source
Slab174	AAV_{retro}-Syn-iCre	1.4e13	Janelia viral core (addgene #122518)
Slab218	AAV_{retro} -CamKII-iCre	2.6e13	Janelia viral core
Slab228	AAV_{retro} -CamKII-GFP	1.1e14	Janelia viral core
Slab185	AAV_{retro} -CAG-GFP	7.7e12	Janelia viral core (addgene #28014)
Slab220	AAVretro-CAG-H2B::TdTomato	6.8e12	Janelia viral core (addgene #116870)
Slab200	AAV2-hsyn-ChR2(H134R)-EYFP-WPRE	5.7e12	UNC vector core
Slab219	AAV2/5-CamKII-hChR2(H134R)-EYFP-WPRE	4.6e12	UNC vector core
Slab213	AAV_{retro} -CamKII-stGtACR1-FusionRed	8.9e12	Janelia viral core, based on a plasmid from Mathias Mahn (addgene #105679)
Slab232	AAV2/5-hsyn-SIO-stGtACR1-FusionRed	7.3e12	Janelia viral core, based on a plasmid from Mathias Mahn (addgene #105678)
Slab143	AAV2/1-hsyn-FLEX-ReachR-Cit	unknown	Janelia viral core (addgene #50955)

Table S3. List of viruses used in this paper (related to STAR Methods)
