# Supplementary material for: A midbrain-thalamus-cortex circuit reorganizes cortical dynamics to initiate movement
Source: Cell. Author manuscript; Available in PMC 2023 Mar 17. (PMC8990337; doi:10.1016/j.cell.2022.02.006)
Supplement: 14 — Table S4. Probe sequences used for HCR. Probes were designed for CDS of chat, slc17a6 and gad1 (Related to STAR Methods) [file NIHMS1784450-supplement-14.pdf]

Chat_CDS1_P1	TTTGGGGGGACCTTTTCCAGGATAG
Chat_CDS1_P2	TCACAGCTAGAAGCTTGTACAGGCA
Chat_CDS2_P1	CTTTTCCTGCAGGGTCTCACCCAGG
Chat_CDS2_P2	GGCTGTCTTCTCCTGTCTCTCCAAG
Chat_CDS3_P1	GTACTGCTTCATACAGAGAGGCTGC
Chat_CDS3_P2	AAGCCGGTATGATGAGAAGAGTCTG
Chat_CDS4_P1	CAAGACAAAGAACTGGTTGCAGCAG
Chat_CDS4_P2	GAGACGGCGGAAATTAATGACAACA
Chat_CDS5_P1	GGAGTCTTTTAAGAGGACCGTCCTG
Chat_CDS5_P2	GATCATGTCCAGGGAGTCCCGGTTG
Chat_CDS6_P1	CATTCAAGCTGCAGCCTCCACCATG
Chat_CDS6_P2	GGGACTTGTCATACCAACGATTCGC
Chat_CDS7_P1	ATATGTTTCAGCAGGTGCTCCGTGC
Chat_CDS7_P2	CGGACGAGCTTCTTGTTGCCTGTCA
Chat_CDS8_P1	TCAGGAGTGGCCGATCTGATGTTGT
Chat_CDS8_P2	GTCATGGCTTGCACAAAAGCCAGAG
Chat_CDS9_P1	CTCTGCAGCAGCTGCAGTTTCTCAG
Chat_CDS9_P2	GTGTACTCAGTTTGGGCCTGGATGG
Chat_CDS10_P1	CATAACAGCAGAACATCTCCATGGT
Chat_CDS10_P2	CTCCATACCCATTGGGTACCACAGG
Chat_CDS11_P1	TTTGCAGCCGTGAAAGCTGGAGATG
Chat_CDS11_P2	TTCTGCAAACTCCACAGATGAGGTC
Chat_CDS12_P1	TGGTTGGGCCTCTAGCTCTTTCCTT
Chat_CDS12_P2	AGTGGGAGTAGTCAAGATTGCTTGG
Slc17a6_CDS1_P1	CTCCAGCACCCTGTAGATCTGTCCG
Slc17a6_CDS1_P2	GATGGTCTCTCGGTTGTCCTGCTTC
Slc17a6_CDS2_P1	AGTGCTGTTGTTGACCATGTCCACG
Slc17a6_CDS2_P2	CTTGATAACTTTGCCTCCGCGGTGG
Slc17a6_CDS3_P1	AATCTGGGTGATGATATAGCCCCAG
Slc17a6_CDS3_P2	CAGCCGCGATGCGATATATCCTCCT
Slc17a6_CDS4_P1	TATCGCAGCCCCAAAGACCCGGTTA
Slc17a6_CDS4_P2	CAGCATATTGAGGGTAGAGGTGAGC
Slc17a6_CDS5_P1	TGTACTGCACAAGGATACCAGCTAA
Slc17a6_CDS5_P2	ACACATAAAATACTGACGACCATCC
Slc17a6_CDS6_P1	ATGTACCAGACCATGCCAAAGCTTC
Slc17a6_CDS6_P2	CTCTCATAAGACACCAGAAGCCAGA
Slc17a6_CDS7_P1	TGAAAAACTTCCTCCATGGGGTCTT
Slc17a6_CDS7_P2	CAATTATCGCGTAGACGGGCATGGA
Slc17a6_CDS8_P1	AAAGTCCAGCTCCTGCAGAAGTTGG
Slc17a6_CDS8_P2	GCTGGCTGACTGATGAGCAGTAAAT
Slc17a6_CDS9_P1	AGACAACATGCCAACCTTGCTGATT
Slc17a6_CDS9_P2	GATTGTCATGACAAGGTGAGGGACT
Slc17a6_CDS10_P1	AAGCAGCGTGGCTTCCATGCCAAAA
Slc17a6_CDS10_P2	TCTAGTATGAGAGTAGCCAACAACC

Slc17a6_CDS11_P1	TAAGATACTGGCATATCTTGGAGCA
Slc17a6_CDS11_P2	CGTGCCAACGCCATTTGAAATGCCC
Slc17a6_CDS12_P1	AGACATACTGCCATTCTTCACGGGA
Slc17a6_CDS12_P2	CATAGTGGACGAGTGCAGCAATGAG
Slc17a6_CDS13_P1	TCTCCTGAGGCAAATAGTGCATAAA
Slc17a6_CDS13_P2	TCCTCAGGGTCTGCCCAAGGTTGTT
Slc17a6_CDS14_P1	TAGCACCGTAAGATTTGGTGGTACC
Slc17a6_CDS14_P2	TAGGCCAGCCTCCATTCTCCTGTGA
Gad1_CDS1_P1	GCTACGCCACACCAAGTATCATACG
Gad1_CDS1_P2	AGGCCCAGTTTTCTGGTGCATCCAT
Gad1_CDS2_P1	GGTCCTTTGTAAGAAGCCACAGATC
Gad1_CDS2_P2	AAGACGACTCTTCTCTTCCAGGCTA
Gad1_CDS3_P1	ACTGCGCAGTTTGCTCCTCCCCGTT
Gad1_CDS3_P2	GGAGTATGTCTACCACTTCCAGCAA
Gad1_CDS4_P1	GTTGAAAAATCGAGGGTGACCTGTG
Gad1_CDS4_P2	AATGATATCCAAACCAGTAGAGAGC
Gad1_CDS5_P1	AGGAGAAAATATCCCATCACCATCT
Gad1_CDS5_P2	GCTGTACATATTGGATATGGCTCCC
Gad1_CDS6_P1	TGACTGTGTTCTGAGGTGAAGAGGA
Gad1_CDS6_P2	GCAGCCCCGGCTTTCTTTATGGAAT
Gad1_CDS7_P1	GGGGAACATAGCCCTTTTGTTTGGC
Gad1_CDS7_P2	TCGTGCCTGCGGTTGCATTGACATA
Gad1_CDS8_P1	CTGAATCGCCTTGTCCCCGGTGTCA
Gad1_CDS8_P2	CTTGAAGATGTCCACATGGCGGCCA
Gad1_CDS9_P1	TCAAATCCCACGGTGCCCTTTGCTT
Gad1_CDS9_P2	AGCTCCAGGCATTTGTTGATCTGGT
Gad1_CDS10_P1	GTGTGCTCAGGCTCACCATCGAAAA
Gad1_CDS10_P2	TGTGGAATGTACCAGAAACAGACAT
Gad1_CDS11_P1	CTTCTGGGAAGTACTTGTAACGAGC
Gad1_CDS11_P2	GCACAGCCGCCATGCCTTTTGTCTT
Gad1_CDS12_P1	TTTGTGGCGGTGCTTCCGGGACATG
Gad1_CDS12_P2	TGAATTGGCCCTTTCTATGCCGCTG

Table S4. Probe sequences used for HCR. Probes were designed for CDS of *chat*, *slc17a6* and *gad1* (Related to STAR Methods)
